# Sulfur-ligated iron(iv)-imido and iron(iv)-oxo complexes, which one is more reactive?

**DOI:** 10.1039/d5sc07586f

**Published:** 2025-11-17

**Authors:** Jagnyesh Kumar Satpathy, Rolly Yadav, Limashree Sahoo, Jens Uhlig, Ebbe Nordlander, Chivukula V. Sastri, Sam P. de Visser

**Affiliations:** a Department of Chemistry, Indian Institute of Technology Guwahati Assam 781039 India sastricv@iitg.ac.in; b Chemical Physics, Department of Chemistry, University of Lund Lund Sweden; c Manchester Institute of Biotechnology, The University of Manchester 131 Princess Street Manchester M1 7DN UK sam.devisser@manchester.ac.uk; d Department of Chemical Engineering, The University of Manchester Oxford Road Manchester M13 9PL UK

## Abstract

Mononuclear iron(iv)-oxo, iron(iv)-imido and iron(iv)-nitrido complexes are common catalytic cycle intermediates in enzymes, where the metal is typically linked to the protein through cysteinate or histidine sidechains. Enzymatic high-valent iron(iv)-imido and -nitrido intermediates have never been trapped and characterized; hence, there is uncertainty regarding their structure and function. Using biomimetic models, we have synthesized a novel N_4_S ligated iron(iv)-imido species as a faithful mimic of a corresponding intermediate in nitrogenase. The complex was characterized with a range of techniques, including UV-vis absorption spectroscopy, electrospray ionization mass spectrometry, resonance Raman spectroscopy and XANES and EXAFS methodologies. A comprehensive investigation combining reactivity studies and computational analysis compares the oxidative reactivity and chemical properties of the iron(iv)-imido complex with those of its oxo-analogue. Although the iron(iv)-oxo species is, in general, more reactive than its iron(iv)-tosylimido counterpart, the reverse trend is observed for the oxidation of specific *para*-substituted thioanisole substrates. Furthermore, an equatorial sulfur group in the ligand framework is seen to enhance the reactivity of thioanisole sulfoxidation. These studies show that high-valent metal-imido groups can be as powerful oxidants as iron(iv)-oxo entities in atom/group transfer reactions.

## Abstract 2

This work highlights the differences in structure, spectroscopic properties and reactivity of sulfur ligated iron(iv)-imido and iron(iv)-oxo complexes and how imido complexes can be as powerful oxidants as oxo complexes.

## Graphical abstract

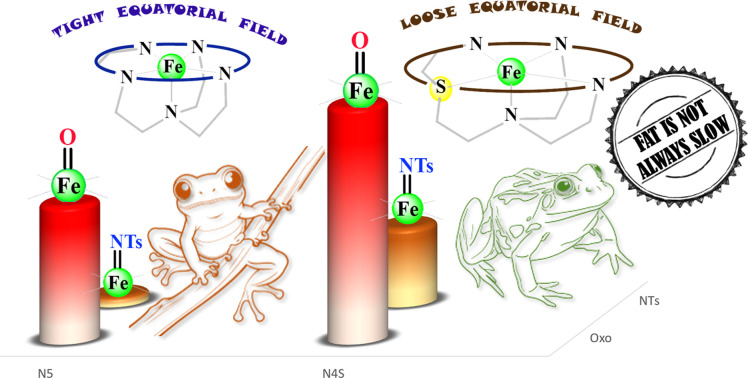

## Introduction

Mononuclear high-valent iron(iv)-oxo compounds are key catalytic cycle intermediates in heme and nonheme iron enzymes and have been trapped and characterized in several mono- or dioxygenases.^1–19^ Such enzymatic intermediates are versatile catalysts that react with substrates through C–H hydroxylation, C=C epoxidation, and heteroatom oxidation as well as the desaturation of aliphatic C–C bonds. In the human body, such biotransformations include vital processes such as the detoxification of xenobiotics in the liver and the biosynthesis of natural products, including signalling molecules and hormones. These high-valent iron(iv)-oxo species are formed during a catalytic cycle and are often short-lived. For instance, in nonheme iron dioxygenases dioxygen binds to an iron(ii) ion and reacts with a co-substrate, typically α-ketoglutarate or biopterin, to form the catalytically active iron(iv)-oxo species.^20–23^ The latter reacts with the substrate in a subsequent reaction through hydrogen atom abstraction or oxygen atom transfer;^7–23^ however, desaturation and ring-closure reactions have also been observed.^24–27^

Interestingly, some of these iron-containing enzymes can also form the corresponding iron(iv)-imido or iron(v)-nitrido entities, which have been characterized or anticipated as intermediates in cytochromes P450 as well as nitrogenases.^28–31^ Metal-imido and/or metal-nitrido intermediates have also been proposed in the catalytic cycle of nitrogenases that bind N_2_ at the FeMo co-factor and reduce it to two ammonia molecules.^31^ The FeMoco is a complex containing seven iron atoms and a molybdenum ion bridged by sulfur groups. As the substrate-binding pocket in nitrogenase is small, there is no evidence of reactivity of the posited nitrogenase iron(iv)-nitrido and iron(iv)-imido species with other substrates. However, studies on the cytochromes P450 have provided experimental evidence that they can catalyze substrate aziridination, presumably through the formation of a high-valent iron(iv)-nitrido or iron(iv)-imido complex.^28–30^ This is an unusual reaction for the P450 class of enzymes as they normally use dioxygen, and with protons from the solvent and electrons from redox partners form an iron(iv)-oxo heme cation radical intermediate that reacts through hydrogen atom and oxygen atom transfer.^1–5^

As enzymatic high-valent metal-oxo, metal-nitrene and metal-imido complexes are short-lived, biomimetic model complexes have been created that contain the metal and their first coordination sphere that are analogous to the enzymatic intermediates but without the protein environment.^32–38^ Several nitrene or imido ligand-based iron–porphyrin model systems have been investigated, exhibiting reactivity towards effective olefin aziridination.^39–43^ In addition, several studies focused on trapping and spectroscopically characterizing nonheme iron(v)-nitrido (Fe^V^≡N) and iron(iv)-imido (Fe^IV^=NR) complexes. Specifically, some of us investigated the comparison of iron(iv)-imido with iron(iv)-oxo complexes and found distinct differences in reactivity between the two.^44,45^ These differences were shown to be triggered by a much larger reduction potential of the iron(iv)-tosylimido group compared to the iron(iv)-oxo species. High-valent metal-nitrido/imido complexes are believed to be able to catalyze isolobal amination processes due to their strong oxidative capability. Several groups have researched the chemistry of metal-catalyzed aminidation of aliphatic C–H bonds and aziridination of alkenes utilizing iminoiodane reagents over the past twenty years.^46–53^ Understanding the basic chemical characteristics of the hypothesised metal–nitrogen multiple bound species has advanced significantly in recent years thanks to their isolation and spectroscopic characterization.^54–62^ However, little is known about their relative reactivities in comparison with the well-known iron(iv)-oxo species, and there is no information on their potential as a nitrogen atom transfer agent.

**Fig. 1:**
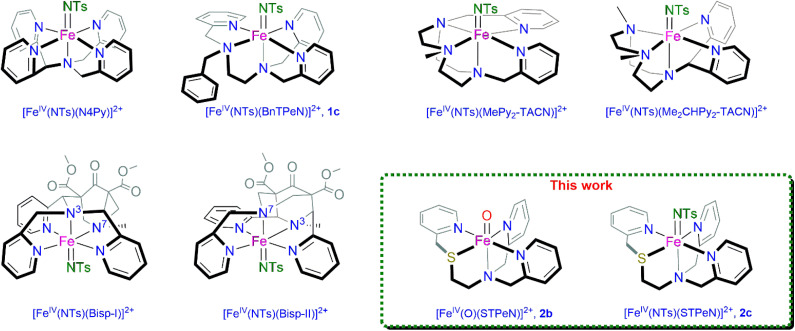
Non-heme iron(iv)-tosylimido [Fe^IV^(NTs)] complexes reported in the literature and structures investigated in this work, namely iron complexes with the BnTPeN (structures 1) and STPeN (structures 2) framework. The definition includes the additional label a for iron(ii) complexes, b for iron(iv)-oxo and c for iron(iv)-tosylimido.

Previously, a comparative study between iron(iv)-oxo and iron(iv)-imido oxidants was performed using the pentadentate N4Py ligand (with N4Py = *N*,*N*-bis(2-pyridylmethyl)-*N*-bis(2 pyridyl)methylamine) as the basic ligand skeleton (see the structure in Fig. 1).^44,45,63^ This ligand was the first example that was shown to support both the high valent iron(iv)-oxo and -imido species.^61–68^ Subsequently, the reactivity of [Fe^IV^(O)(N4Py)]^2+^*versus* [Fe^IV^(NTs)(N4Py)]^2+^ (NTs = tosylimido) with selected substrates was explored and the results showed that the iron(iv)-oxo and iron(iv)-tosylimido complexes react *via* different reaction mechanisms.^44,45^ In particular, the iron(iv)-oxo complex reacted with thioanisole through a concerted and direct oxygen atom transfer reaction, whereas the corresponding iron(iv)-imido complex reacted *via* an initial electron transfer instead. Latour *et al.* reported the synthesis and characterization of two octahedral iron(iv)-imido complexes with MePy_2_TACN and Me_2_(CHPy_2_)TACN ligand frameworks (MePy_2_TACN = *N*-methyl-*N*,*N*-bis(2-picolyl)-1,4,7-triazacyclononane; Me_2_(CHPy_2_)TACN = 1-(di(2-pyridyl)methyl)-4,7-dimethyl-1,4,7-triazacyclononane; Fig. 1 right-hand-side).^68^ Recently, Comba and co-workers reported an extensive study on the comparative oxidative reactivity between iron(iv)-oxo and iron(iv)-tosylimido complexes bearing two similar bispidine ligand frameworks designated Bisp-I and Bisp-II, Fig. 1 bottom left.^69,70^ They observed different reactivity patterns for the two ligand systems, whereby the iron(iv)-tosylimido complex with Bisp-I ligand was more reactive with thioanisole as compared to the analogous complex with Bisp-II ligand. By contrast, the iron(iv)-oxo complex was more reactive with the Bisp-II ligand framework. Our groups have also studied the comparative reactivity of iron(iv)-oxo and iron(iv)-imido complexes with the BnTPeN ligand framework (Fig. 1, top row).^63^ With the BnTPeN ligand system, the iron(iv)-imido complex produced sluggish reactivity for *S*-oxygenation and C–H activation reactions. As such, these iron(iv)-tosylimido complexes show highly versatile reactivity that appears to be sensitive to the ligand environment to which the metal is bound. All of these studies considered an *N*_5_-pentadentate ligand framework, where the metal is covalently bound to five nitrogen atoms of a single ligand system. In biological nonheme iron systems there are major differences in reactivity seen between the cytochromes P450 with a symmetric ligand framework and the nonheme iron dioxygenases that lack this. Furthermore, recent computational studies on biomimetic iron(iv)-oxo complexes showed that incorporation of a sulfur atom into the ligand framework enhanced the reactivity due to lowering of the HOMO–LUMO energy gap.^71–73^ As such we created novel ligands that disrupt the symmetry of the *N*_5_ framework by integrating a sulfide group in the equatorial position. We, therefore, synthesized iron complexes of a series of novel *N*_4_*S*-pentadentate ligand systems designated STPeN (Fig. 1, bottom right) and studied their spectroscopy and reactivity with model substrates. These newly developed complexes should give better links with biological systems and describe the first-coordination sphere interactions of iron(iv)-oxo and iron(iv)-imido entities more realistically when compared to nonheme iron dioxygenases as well as the nitrogenase cofactor. Thus, the present work gives an in-depth discussion on the reactivity of [Fe^IV^(NTs)(STPeN)]^2+^ (2c) and [Fe^IV^(O)(STPeN)]^2+^ (2b) with respect to *S*-oxidation and C–H activation. We report here a sulfur ligated iron(iv)-imido complex that is stable under ambient reaction conditions. The new complexes were characterized using a variety of spectroscopic tools including Extended X-ray Absorption Fine Structure (EXAFS) and resonance Raman spectroscopy approaches, and their oxidative reactivities were explored with model substrates. A detailed structural and reactivity analysis of the iron(iv)-imido complex with its iron(iv)-oxo analogue is reported and shows reaction conditions where the imido complex reacts more rapidly than the oxo analogue.

## Experimental section

### Materials and methods

All chemicals were purchased from Sigma Aldrich Chemical Co. and were of the best available purity, and were used without further purification unless otherwise stated. Solvents were dried according to published procedures and freshly distilled under argon gas prior to use.^74^ The pentadentate ligands BnTPeN and STPeN (see Fig. 1) were synthesized with slight modifications from the reported procedures in the literature.^62–65,75^ The iron(ii) complexes [Fe^ii^(BnTPeN)(CH_3_CN)](OTf)_2_ (1a) and [Fe^ii^(STPeN)(CH_3_CN)](OTf)_2_ (2a) were synthesized using dry acetonitrile as the solvent inside a Jacomex glove box filled with argon gas. The iron(iv)-oxo (2b) and iron(iv)-tosylimido (2c) complexes with the STPeN ligand framework were synthesized in acetonitrile under ambient conditions. Deuterated ethylbenzene, deuterated toluene and deuterated benzyl-alcohol, namely ethylbenzene-[*D*_10_], toluene-[*D*_8_] and benzyl-alcohol-[*D*_7_], were procured from Cambridge Isotope Laboratories. Deuterated fluorene, namely fluorene-[*D*_2_], was synthesized using an earlier reported procedure.^76^

### Instrumentation

UV-vis spectra and kinetic experiments were recorded on a Hewlett-Packard 8453 spectrophotometer equipped with either a constant temperature circulating water bath or a liquid nitrogen cryostat (Unisoku) with a temperature controller. NMR spectra (^1^H, and ^13^C) were obtained with a Bruker Avance III HD 400 MHz or 600 MHz NMR spectrometer using tetramethylsilane as the internal standard. High resolution electrospray ionization-mass spectrometry (ESI-MS) spectra of the iron(ii) and iron(iii) complexes were recorded on an Agilent G6546A series (UHPLC-QTOF-HRMS) mass spectrometer at 298 K with a 2 kV spray voltage and 80 °C capillary temperature. The cyclic voltammetry experiments were carried out at room temperature using a CH Instruments Electrochemical Analyzer CHI1120B series. A three-electrode system was used with a glassy carbon electrode as the working electrode, Pt wire as the auxiliary electrode, and aqueous Ag/AgCl as the reference electrode. The solutions used were 1 mM of 1a and 2a, and 100 mM supporting electrolyte *tetra-n*-butylammonium hexafluorophosphate (TBAPF_6_) in acetonitrile. The resonance Raman spectra of 2c were obtained at 561 nm (80 mW, Cobolt lasers, HÜBNER Photonics) excitation wavelength using a Kymera 328i motorized Czerny–Turner Spectrograph (Andor Technology) equipped with a DU 420A-BEX2-DD camera (iDus 420 CCD, Andor Technology). The CCD camera was cooled to −80 °C. The spectral slit width of the instrument was set to 120 µm. Single crystal XRD measurements were done at room temperature using a single source Super Nova CCD System instrument from Agilent Technologies equipped with a fine focus 1.75 kW sealed tube with Mo Kα radiation. The data were reduced using CrysAlis RED36. The structure solution and refinements were performed using the SHELXL97 and Olex2 1.5 programs. The checkCIF was submitted to the repository of the CCDC (under number 2310147).

The XANES/EXAFS data were collected at the Balder endstation of the MaxIV laboratory. The data were collected in transmission mode using ionization chambers as detectors. The data were calibrated to 7112 eV using iron foil. The samples were chemically oxidized using 1–1.5 equivalents of ^s^PhIO (^s^PhIO = 2-(tmklert-butylsulfonyl)iodosylbenzene) as the oxidizing agent to a 10 mM iron(ii) complex in acetonitrile. The samples were prepared freshly and loaded in a small PEEK cavity with 3 mm pathlength. The cavity is closed with 50 µm thick Kapton tape on both sides and measured within 2 min after preparation. During longer measurements some of the sample did leak out of the cell, which was however directly visible due to the strong change in absorption. No sample degradation was detectable in the XANES taken at the beginning and end of each measurement. The sample thickness variations due to foil bending or bubbling were not detectable during each measurement. The structural refinement was achieved using the Artemis software package and followed the standard procedures.^77,78^

### Reactivity studies

All the reactions were run in a 10 mm path length quartz cuvette by monitoring the UV-vis spectral changes of the reaction solutions as a function of time. The formation of the intermediate was monitored from the increase in absorbance at its characteristic wavelength in the UV-vis spectrum as a function of time. The rate constants were determined under pseudo first-order conditions with excess substrate concentration with respect to the oxidant. The decrease in absorbance of the characteristic peak was monitored as a function of time to obtain the pseudo first-order rate constant (*k*_obs_) which was further plotted against substrate concentration to get the second-order rate constant (*k*_2_) for a particular reaction. All the reactions were run in triplicate and averaged to ensure standard deviation of less than 10% of the obtained values.

### Synthesis of [Fe^IV^(O)(STPeN)](OTf)_2_

The [Fe^IV^(O)(STPeN)]^2+^ complex was generated *in situ* by reacting the ferrous precursor complex in acetonitrile with 1.5 equivalent of ^s^PhIO in trifluoroethanol using a UV-vis spectrophotometer. Within ∼20 s the color of the mixture changed to light green, and we observed a d–d transition band at 756 nm with a molar absorption coefficient of 380 M^−1^ cm^−1^.

### Synthesis of [Fe^IV^(NTs)(STPeN)](OTf)_2_

The [Fe^IV^(NTs)(STPeN)]^2+^ complex was generated *in situ* by reacting the ferrous precursor complex in acetonitrile with 1.5 equivalent of ^*s*^PhINTs (^*s*^PhINTs = 2-(tert-butylsulfonyl)(*p*-toylsulfonyliminoiodo)benzene) in dichloromethane using a UV-vis spectrophotometer. Within ∼20 s the color of the mixture changed to light green and a d–d transition band at 730 nm with a molar absorption coefficient of 250 M^−1^ cm^−1^ was observed.

### Computational methodology

All computations were performed using density functional theory (DFT) methods in the Gaussian-09 software package.^79^ The unrestricted B3LYP density functional method was used for all calculations.^80,81^ Previous work showed that this method reproduced experimental product distributions and rate constants well.^82–84^ In particular, test calculations using the B3LYP method with a solvent model included reproduced experimentally determined free energies of activation for reactions of an iron(iv)-oxo species with thioanisole.^85^ Geometry optimizations, analytical frequencies and constraint geometry scans were performed with basis set BS1 which employs LANL2DZ with a core potential on iron and 6-31G* on the remaining atoms.^86,87^ The calculations were conducted in an implicit solvent model with the dielectric constant resembling acetonitrile (*ε* = 35.688) and utilizing a continuous polarizable continuum model (CPCM).^88^ Frequency calculations were performed to determine whether the structures were local minima or transition states and an analysis of the imaginary frequency confirmed the transition. Single-point computations used the LACV3P+ basis set with triple-ζ-effective core potential on iron and 6-311G* on other atoms: basis set BS2. Test calculations on the spin state ordering of complexes ^3,5^1b, ^3,5^1c, ^3,5^2b and ^3,5^2c were done through full geometry optimizations at the UB3LYP-GD3BJ/BS2, OLYP/BS2 and PBE0/BS2 level of theory with a solvent model that mimics acetonitrile, see the SI.^80,81,86–91^ The UB3LYP-GD3BJ results give the same spin state ordering as and similar relative energies to those obtained with the OLYP method, whereas the PBE0 approach stabilizes the high-spin states stronger in disagreement with experiment. Therefore, the B3LYP approach was selected here.

## Results and discussion

**Fig. 2:**
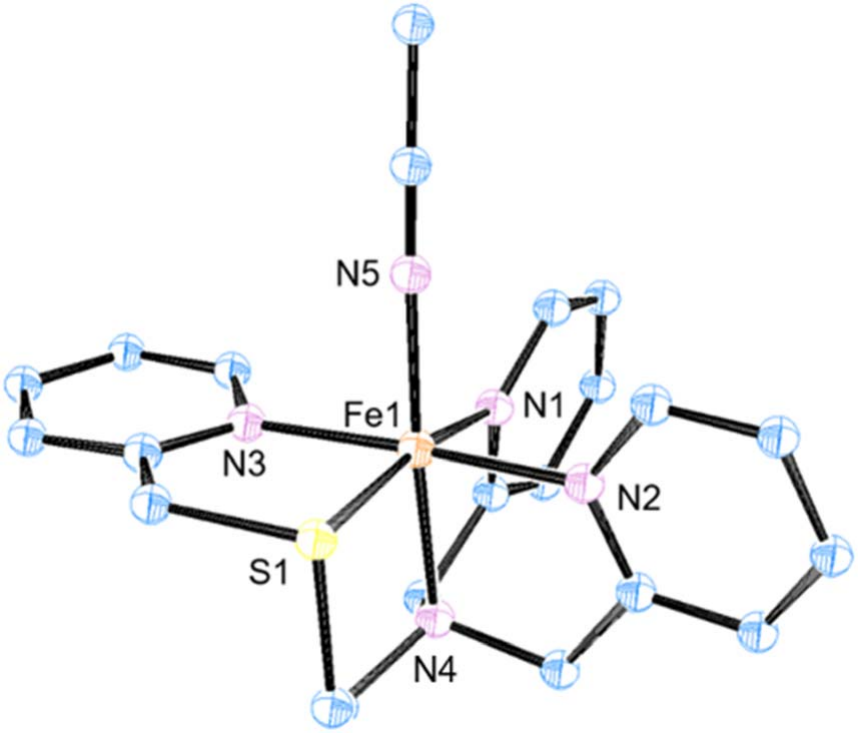
ORTEP image for [Fe(STPeN)(OTf)]^+^ (2a). Hydrogen atoms and counterions have been omitted for better clarity; thermal ellipsoids are plotted with 30% probability.

The ligands BnTPeN, *i.e.*, *N*^1^-benzyl-*N*^1^,*N*^2^,*N*^2^-tris(pyridine-2-ylmethyl) ethane-1,2-diamine, and STPeN, *i.e.*, 2-(2-pyridinyl)-methylthio-*N*,*N*-bis[(2-pyridinyl)methyl]ethanamine, were synthesized according to previously reported procedures and characterized by ^1^H and ^13^C nuclear magnetic resonance (NMR) spectroscopy (see Fig. S1–S4, SI).^75^ Subsequently, the BnTPeN and STPeN ligands were reacted with [Fe^II^(CH_3_CN)_2_(OTf)_2_] (OTf = triflate), in acetonitrile under an inert atmosphere inside a glovebox, which formed the corresponding iron(ii) complexes [Fe^II^(BnTPeN)(CH_3_CN)](OTf)_2_ (1a) and [Fe^II^(STPeN)(CH_3_CN)](OTf)_2_ (2a). These iron(ii) complexes were characterized by UV-vis absorption spectroscopy, cyclic voltammetry (CV), electrospray ionization mass spectrometry (ESI-MS) and X-ray crystallography (see Fig. 2 and S5–S7, SI).

The characterization data match the previous work on iron(ii) complexes with the BnTPeN ligand framework.^62–66,92^ The UV-vis spectra of both iron(ii) complexes show a ligand to metal charge-transfer band with notable differences in the molar extinction coefficient (*ε*) and *λ*_max_ values. For 1a we obtained *ε*_1a_ = 8046 M^−1^ cm^−1^ (*λ*_max,__1a_ = 386 nm), whereas the corresponding value for complex 2a is *ε*_2a_ = 8469 M^−1^ cm^−1^ (*λ*_max,__2a_ = 372 nm), see Fig. S5 in the SI. Thus, a hypsochromic shift (by 14 nm) with increased molar absorption coefficient is obtained through insertion of a sulfur atom in the equatorial plane of the complex. This shift is smaller when compared to an earlier study on iron(ii) complexes with N_5_ and N_4_S coordination, where a hypsochromic shift of 60 nm was observed.^93^ Moreover, cyclic voltammetry studies of the iron(ii) complexes 1a and 2a exhibited a reversible/quasi-reversible Fe^II^/Fe^III^ couple with a difference of 140 mV in the redox potentials of the two complexes, namely values of *E*_1/2_ of 870 mV for 1a and *E*_1/2_ of 1010 mV for 2a (SI, Fig. S6).

The crystal structure shown in Fig. 2 of 2a illustrates its geometry to be pseudo-octahedral. The sulfur atom is found in an equatorial position with respect to the acetonitrile molecule that is bound to the iron center in the distal position (Fig. 2), where we define the site *trans* to the vacant site as the axial position. The three pyridine rings are in the equatorial plane with two of these on opposing sites of the metal with the π-system in the same plane. The amine nitrogen atom is in the axial position to iron. The average Fe–N and Fe–S bond distances and the corresponding bond angles are listed in Table S1, SI. These distances match those of analogous complexes reported previously.^62–66^ The ESI-MS spectra of 1a and 2a give peaks at *m/z* = 628.06 and 555.04 corresponding to the [Fe^II^(BnTPeN)OTf]^+^ and [Fe^II^(STPeN)OTf]^+^ fragment ions, respectively (Fig. S7, SI). The isotopic distribution pattern for the complexes confirms their assignments.

**Fig. 3:**
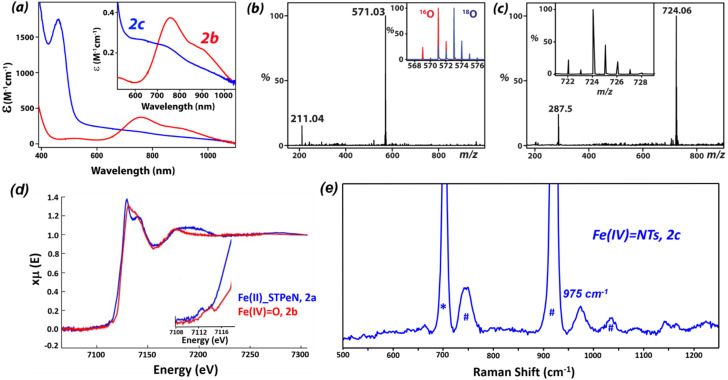
(a) UV-vis spectra of 2b (red) and 2c (blue) in CH_3_CN at 298 K. (b) Electrospray ionization mass spectrum of 2b in CH_3_CN at 298 K. The inset shows expanded isotopic distribution patterns of [Fe^IV^(^16^O)(STPeN)(OTf)]^+^ (in red) and [Fe^IV^(^18^O)(STPeN)(OTf)]^+^ (in blue). (c) Electrospray ionization mass spectrum of 2c in CH_3_CN at 298 K. The inset shows expanded isotopic distribution patterns of [Fe^IV^(NTs)(STPeN)(OTf)]^+^. (d) Normalized Fe K-edge X-ray absorption spectra of 2a (blue) and 2b (red) in CH_3_CN, inset shows the pre-edge region for the complexes. (e) Resonance Raman spectrum of 2c at 298 K at 561 nm excitation wavelength. The symbols * and # indicate the solvent peaks.

The Fe^IV^=O and Fe^IV^=NTs complexes with the BnTPeN ligand framework, *i.e.*, [Fe^IV^(O)(BnTPeN)]^2+^ (1b) and [Fe^IV^(NTs)(BnTPeN)]^2+^ (1c) were studied previously.^63^ We were unable to synthesize the corresponding complexes with the STPeN framework using the same procedures. Therefore, an alternative procedure was developed through treatment of [Fe^II^(STPeN)(CH_3_CN)](OTf)_2_ with 1.5 equivalents of 2-*tert*-butylsulfonyliodosylbenzene (^s^PhIO) and 2-*tert*-butylsulfonyl*-N*-tosyliminophenyliodinane (^s^PhINTs) in acetonitrile under ambient conditions, which generated the corresponding iron(iv) complexes: [Fe^IV^(O)(STPeN)]^2+^ (2b) and [Fe^IV^(NTs)(STPeN)]^2+^ (2c). The complexes were initially characterized by UV-vis spectroscopy and 2b had absorption values of *λ*_max_ = 756 nm and *ε* = 380 M^−1^ cm^−1^, whereas for 2c they were *λ*_max_ = 730 nm and *ε* = 250 M^−1^ cm^−1^ (Fig. 3a). Table 1 summarizes the characterization of various iron(iv)-oxo and iron(iv)-tosylimido complexes reported previously and compares the data with the current results on the system with the STPeN ligand.^44,45,68–70^ Thus, the substitution of the N–CH_3_ moiety in BnTPeN with a sulfur atom to form STPeN results in a red shift of the d–d transition band by 17 nm for the iron(iv)-oxo and 80 nm for the iron(iv)-tosylimido complexes. On the other hand, structure 2c gives a *λ*_max_ value that matches the corresponding bispidine complexes well, although the *λ*_max_ value appears to be very different when compared to the related N4Py ligated structures. As such, the three complexes possess different excited state profiles resulting in absorption changes that may further result in reactivity differences with substrates. Complex 2b has a half-life of *t*_1/2_ = 60 min at RT, whereas it is 45 min at RT for 2c. The ferryl-oxo and ferryl–tosylimido complexes were further characterized by ESI-MS spectrometry, resonance Raman spectroscopy and also X-ray absorption spectroscopy, see Fig. 3.

**Table 1:** Spectroscopic parameters (UV-vis-NIR, resonance Raman (rR) and EXAFS data) of the Fe^IV^(O) and Fe^IV^(NTs) complexes

	N4Py^44,45,62^	BnTPeN (1)^63^	Me_2_CHPy_2_TACN^68^	Bisp-I^70^	Bisp-II^70^	STPeN (2)
**UV-vis-NIR, *λ***_**max**_ **(nm)**						
Fe^IV^=O	695	739	740	730	730	756
Fe^IV^=NTs	660	650	740	735	730	730

**rR, stretching vibration (cm** ^**−1**^ **)**						
Fe^IV^=O	841	835	839	840	825	NA
Fe^IV^=NTs	998	984	1016	NA	NA	975

**EXAFS, (Å)**						
Fe^IV^=O	1.64	1.67	1.63	1.62	1.64	1.68
Fe^IV^=NTs	1.73	1.76^a^	1.72	1.76^a^	1.77^a^	1.76^a^

a Computed value for Fe=N bond lengths.

The ESI-MS spectrum of 2b shows prominent peaks at *m*/*z* 571.03 and *m*/*z* 211.04 corresponding to the [Fe^IV^(O)(STPeN)(OTf)]^+^ and [Fe^IV^(O)(STPeN)]^2+^ ions, respectively (Fig. 3b). The isotopic distribution patterns for the two species confirm their assignments. The formation of the [Fe^IV^(O)(STPeN)(OTf)]^+^ species was further established by an isotopic labeling experiment using H_2_^18^O, which leads to oxygen atom exchange with the oxo group. The experiment with H_2_^18^O moves the peak in the ESI-MS spectrum for [Fe^IV^(O)(STPeN)(OTf)]^+^ from *m*/*z* 549.14 to *m*/*z* 551.14, while the peak representing the [Fe^IV^(O)(STPeN)]^2+^ ion shifts by one unit. Hence the isotopic labelling experiment confirms that one oxygen atom is incorporated into the metal complex in the form of an iron(iv)-oxo species. Similarly, the ESI-MS spectrum of 2c shows prominent peaks at *m*/*z* 724.06 and *m*/*z* 287.50 corresponding to the [Fe^IV^(NTs)(STPeN)(OTf)]^+^ and [Fe^IV^(NTs)(STPeN)]^2+^ ions, respectively (Fig. 3c). The isotopic distribution patterns for the two complexes confirm their assignments. ^1^H NMR analysis of both ferryl complexes, [Fe^IV^(O)(STPeN)]^2+^ (2b) and [Fe^IV^(NTs)(STPeN)]^2+^ (2c), gives characteristic shifts in the NMR spectra of the S = 1 Fe centres in CD_3_CN (Fig. S12 and S13, SI).^94^ The overall NMR spectra for 2b and 2c are similar to those of the 1b and 1c complexes with the BnTPeN ligand framework.^94^ As such, we are confident that no isomeric structures of the complex are stable as shown by crystallography and DFT calculations on the iron(iv)-oxo species with the BnTPeN ligand system.

The resonance Raman (rR) spectra of 2c obtained at 561 nm excitation wavelength feature a resonantly enhanced band at 975 cm^−1^, which was absent in the starting iron(ii) complex and also not present in the spectrum of the solvent. In analogy with the previously reported Fe^IV^=NTs complexes,^62,68^ we have assigned this band as that originating from the Fe=N (975 cm^−1^) stretching vibration, see Fig. 3e. The Fe^IV^=N stretching vibration for the corresponding parent [Fe^IV^(NTs)(BnTPeN)(OTf)]^+^ complex is located at 984 cm^−1^. Hence, the Fe^IV^=N stretch in 2c is red shifted by 9 cm^−1^ upon the introduction of a sulfur atom in the ligand framework. We were unable to obtain a resonance Raman spectrum for complex 2b, due to the conversion of Fe^IV^=O to Fe^II^ upon laser excitation at 405 nm.

The presence of the Fe=O and Fe–S bonds in complex 2b was confirmed through EXAFS analysis, which yielded a best-fit plot (Fig. 3d and S15 and S16, SI) with the O/N scattered at 1.68 Å (assigned to the Fe=O/Fe=NTs unit). In addition, the EXAFS data shows a further shell of five O/N scattered at 2.13 Å (corresponding to the *N* donors of the STPeN ligand) while the Fe–S distance scatters at 2.43 Å. The Fe=O bond length observed for [Fe^IV^(O)(BnTPeN)]^2+^ is 1.67 Å, while it was 1.64 Å for [Fe^IV^(O)(N4Py)]^2+^.^62,95^ Thus, upon the introduction of a sulfur atom into the ligand framework of BnTPeN to obtain STPeN there is little change in the Fe=O bond length. The Fe K-edge X-ray absorption spectrum of 2b (Fig. 3d) revealed an edge energy of 7126.8 eV (*vs.* 7123.7 eV for [Fe^IV^(O)(BnTPeN)]^2+^) and a broad pre-edge peak assigned to 1s–3d transitions at 7114.6 eV as compared to its iron(ii) precursor 2a, which has the pre-edge peak at 7112.4 eV. This chemical shift is a strong indication of the changes in the oxidation state.

Next, the reactivities of 2b and 2c were evaluated through investigations of their performances in two-electron heteroatom oxidation reactions as well as through the activation of aliphatic C–H bonds. In particular, thioanisole was employed as the substrate to assess the heteroatom transfer, *i.e.*, oxygen atom transfer (OAT) or tosylimido transfer reactivity, while a range of substrates with varying C–H bond strengths were tested for hydrogen atom transfer (HAT) pathways. The addition of substrates to a solution containing 2b or 2c in acetonitrile led to the decay of the characteristic d–d transition band in the UV-vis spectrum, which enabled us to obtain their pseudo-first-order rate constants. The second-order rate constants (*k*_2_) were subsequently determined by plotting the pseudo-first-order rate constants (*k*_obs_) as a function of the relative increment of the substrate concentration, which gave a linear correlation.

**Fig. 4:**
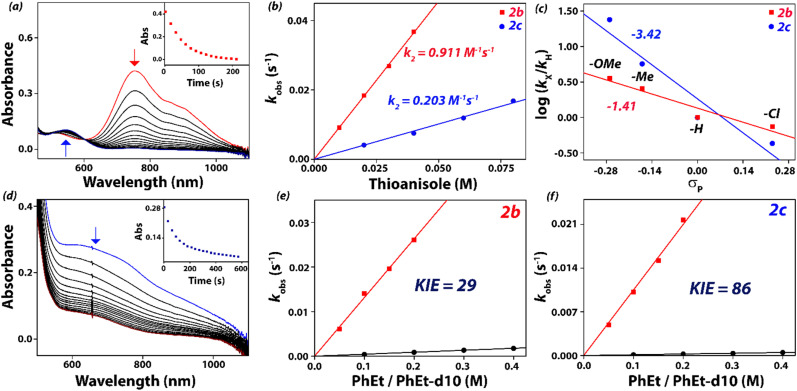
(a) UV-vis spectral changes of 2b upon addition of 20 equiv. of thioanisole in CH_3_CN at 263 K. Inset shows the decay profile of the 756 nm band. (b) The second-order rate constant determined for the reaction of 2b (1 mM) and 2c (1 mM) with thioanisole at 263 K. (c) plot of log(*k*_X_/*k*_H_) against Hammett parameter (*σ*_p_) for the reactions of *para*-X-thioanisole with 2b and 2c at 263 K in CH_3_CN, where *k*_X_ and *k*_H_ are the pseudo first-order rate constants of *para*-X-thioanisole and thioanisole, respectively. (d) UV-vis spectral changes of 2c upon addition of 100 equiv. of ethylbenzene in CH_3_CN at 263 K. Inset shows the decay profile of the 660 nm band. (e) Plots of the pseudo-first order rate constants *k*_obs_*vs.* the substrate concentrations for ethylbenzene and ethylbenzene-[D_10_] and the obtained kinetic isotopic effect (KIE) for the reaction with 2b with ethylbenzene in CH_3_CN at 298 K. (f) Plots of the pseudo-first order rate constants *k*_obs_*vs.* substrate concentration for ethylbenzene and ethylbenzene-[D_10_] and the obtained KIE for the reaction of 2c with ethylbenzene in CH_3_CN at 298 K.

The addition of thioanisole to 2b at 263 K led to the decay of the iron(iv)-oxo characteristic band at 756 nm in the UV-vis spectrum (Fig. 4a), thereby producing methylphenylsulfoxide as the major product. We measured the change in absorbance from the UV-vis spectra at 756 nm and plotted it as a function of time, which enabled us to determine the pseudo-first-order rate constant (*k*_obs_) for the reaction. Subsequently, these observed rate constants were converted into second-order rate constants by plotting *k*_obs_ as a function of substrate concentration. Generally, sulfoxidation reactions proceed *via* a concerted oxygen atom transfer to the sulfur atom of the organic substrate,^93,95^ resulting in the formation of sulfoxides as the oxidized products. The second-order rate constant (*k*_2_) for the reaction of thioanisole with 2b was evaluated to be 0.911 M^−1^ s^−1^ at 263 K. By contrast, the reaction of thioanisole with 2c under the same reaction conditions provided a second-order rate constant of 0.203 M^−1^ s^−1^. Thus, under the same experimental conditions, the reactivity of 2b with thioanisole is faster than that of 2c by a factor of almost four (Fig. 4b). To put these values into perspective, we summarize the thioanisole sulfoxidation and sulfimidation rate constants reported for the iron(iv) systems with N4Py, BnTPeN, Me_2_CHPy_2_TACN, Bisp-I, Bisp-II and STPeN ligand frameworks in Table 2. As some measurements were reported at different temperatures, we converted the reaction rates to 298 K values by using the approximation by the van ’t Hoff rule. The observed reactivity trend for 2b and 2c is consistent with the pattern of reactivity for 1b and 1c. Therefore, under similar reaction conditions, the reactivity of thioanisole with 2b is nearly three times faster as compared to that of 1b. On the other hand, [Fe^IV^(NTs)(BnTPeN)]^2+^, complex 1c, was shown to have sluggish reactivity as compared to [Fe^IV^(O)(BnTPeN)]^2+^, 1b.^63^ By contrast, the reactivity difference between [Fe^IV^(NTs)(STPeN)]^2+^ (2c) and [Fe^IV^(NTs)(BnTPeN)]^2+^ (1c) is by a factor of around 90 in favour of 2c. Therefore, introduction of a sulfur atom in the equatorial position of the ligand framework drastically enhances the oxidative reactivity for the Fe^IV^=NTs complex. The reaction rates measured for 2b and 2c are also significantly faster than the corresponding ligand complexes with N4Py or Me_2_CHPy_2_TACN ligands.

**Table 2:** Second-order rate constants for the reactions of [Fe^IV^=O]^2+^ and [Fe^IV^=NTs]^2+^ complexes with thioanisole at 298 K^a^

	Fe^IV^=O complexes		Fe^IV^=NTs complexes	
	*k*_2_ (M^−1^ s^−1^) for thioanisole		*k*_2_ (M^−1^ s^−1^) for thioanisole	
N4Py	0.49	(0.05)^b^	2.6	(0.26)^b^
BnTPeN (1b/1c)	8.42	(0.33)^c^	0.054	
Me_2_CHPy_2_TACN	0.14	(0.091)^d^	0.012	(0.0079)^d^
Bisp-I	5.65		9.77	
Bisp-II	480		117	
STPeN (2b/2c)	**17.81**	(**0.911)^c^**	**4.76**	(**0.203)^c^**

a Values in Columns 3 and 5 recalculated for a standard temperature and approximated with the van ’t Hoff rule using a correction factor of 2.5 per 10 K.

b *T* = 273 K.

c *T* = 263 K.

d *T* = 293 K.

To gain insight into the mechanistic details of the reaction pathway, we studied the reactions of 2b and 2c with various *para*-X-substituted thioanisole substrates (X = OCH_3_, CH_3_, H, Cl) and measured their reaction rates (Fig. 4c). These *para*-substituents influence the charge distribution and thereby give a measure of the strength of electron transfer in the rate-determining transition state that is measured from the slope of the plot of the natural logarithm of the rate constant *versus* the Hammett *σ*_p_ parameter.^96^ Second-order rate constants were measured for each substrate (SI, Table S3) and plotted as the logarithm of the rate constant ratio (*k*_X_/*k*_H_) as a function of the *σ*_p_ Hammett values (Fig. 3c). It is known that a significant slope in the Hammett plot is indicative of an early electron transfer pathway, whereas a smaller Hammett slope typically indicates an oxygen atom transfer pathway.^45,97^ A plot of the logarithm of the rate constant ratio (*k*_X_/*k*_H_) as a function of *σ*_p_ values of various *par*a-X-substituted thioanisole substrates gives a linear correlation with a slope of *ρ* = −1.41 and *ρ* = −3.42 for 2b and 2c, respectively (Fig. 4c). The larger Hammett slope is due to the additional stabilization of positive charge in the transition state by electron donating groups in the *para*-position of thioanisole and thus enhances the overall rate of oxidation. Similarly, when the rates are plotted against the one electron oxidation potential *E*^0^_ox_ values for various *para*-X-substituted thioanisole substrates, it produces a linear correlation with a slope of −2.88 and −7.12 for 2b and 2c, respectively. From the slopes of the Hammett plots, therefore, it follows that thioanisole reacts with 2b and 2c through a different reaction mechanism. Furthermore, the Hammett plots indicate that thioanisole sulfoxidation by 2b has a rate-determining oxygen atom transfer step through an electrophilic reaction mechanism, whereas the reaction by 2c proceeds through an electron-transfer pathway. This is in line with our previous work on [Fe^IV^(O)(N4Py)]^2+^*versus* [Fe^IV^(NTs)(N4Py)]^2+^ which showed that the tosylimido complex is more easily reduced by one and two electrons and shows early electron transfer steps in a reaction with the substrate.^44,45^

We then studied the C–H activation ability of 2b and 2c with a selection of substrates with known C–H bond strengths. Earlier studies demonstrated that reactions involving a rate-determining hydrogen atom abstraction exhibit a linear relationship between the natural logarithm of the rate constant and the C–H bond dissociation energy (BDE_C–H_).^98–101^ We investigated the hydrogen atom abstraction reactions of 2b and 2c with substrates, including triphenylmethane, cumene, ethylbenzene, and toluene. These substrates span a range of C–H bond dissociation energies (BDE_C–H_) ranging from 81 to 90 kcal mol^−1^. In reactions with substrates containing C–H bonds including triphenylmethane, cumene, ethylbenzene, and toluene, 2b reacts at least 2–4 times faster than 2c. In order to gain insight into the reaction pathway involved during the C–H bond activation reaction, we investigated the reaction of 2b and 2c with various *para*-X-substituted ethylbenzene substrates (X = –OMe, –Me, –H, –Br) and measured their reaction rates. Similar to the reaction with various *para*-substituted thioanisole derivatives, the *para*-substituted ethylbenzene compounds reveal the influence of charge distribution and thereby give a measure of the strength of electron transfer in the rate-determining transition state that is determined from the slope of the plot of the rate constant *versus* the Hammett *σ*_p_ parameter.^96,102–105^ Second-order rate constants were measured for the reaction of 1b and 2b with each substrate (SI, Table S4) and plotted as the logarithm of the rate constant ratio (*k*_X_/*k*_H_) as a function of the *σ*_p_ Hammett values.^96,102–105^ A plot of the logarithm of the rate constant ratio (*k*_X_/*k*_H_) as a function of *σ*_p_ values of various *para*-X-substituted ethylbenzene substrates gives a linear correlation with a slope of *ρ* = −2.27 and *ρ* = −3.89 for 2b and 2c, respectively (Fig. S23, SI). The larger Hammett slope for 2c may be due to the additional stabilization of positive charge in the transition state by electron donating groups in the *para*-position of ethylbenzene and resulting in the enhancement of the overall rate of oxidation. Furthermore, a detailed kinetic isotope effect study was explored with ethylbenzene and ethylbenzene-[*D*_10_] as substrates. In both experiments, the second-order rate constant is considerably lower when ethylbenzene-[*D*_10_] was employed leading to a kinetic isotope effect (KIE, *k*_H_/*k*_D_) of KIE_2b_ = 29 for the reaction of ethylbenzene with 2b and a KIE_2c_ = 86 for the reaction of ethylbenzene with 2c as an oxidant (Fig. 4e and f). Generally, a lower KIE (<10) is expected for Fe(iv)=NTs complexes, but, in the case of 2c, a larger KIE value of 86 indicates that nonclassical events take place such as quantum mechanical tunnelling that will *de facto* reduce the hydrogen transfer barrier significantly in the reaction of 2c with ethylbenzene. Moreover, these KIE values imply that both oxidants react through an initial and rate-determining hydrogen atom abstraction pathway.

To find evidence of the rate-determining initial hydrogen atom transfer reaction, we studied the KIE of both 2b and 2c with other substrates, including fluorene, toluene and benzyl alcohol as model substrates (see the SI, Fig. S22–S27 and Table S6). The KIE values observed for 2b with fluorene, toluene and benzyl alcohol are 23, 10 and 28, which fall in the same region as that of earlier reported model Fe(iv)=O complexes. However, interestingly, the KIE values observed for 2c are much lower, namely KIE = 3, 4 and 7 for fluorene, toluene, and benzyl alcohol, respectively. This observation suggests a change in reaction pathway from an initial hydrogen atom transfer to an initial hydride transfer, as seen in our previous studies where a lowering of KIE was seen with xanthene.^93^ To get a clearer mechanistic insight into the reactivity in C–H activation by 2b and 2c, we have also employed DFT studies with model substrates.

### Computational studies

**Fig. 5:**
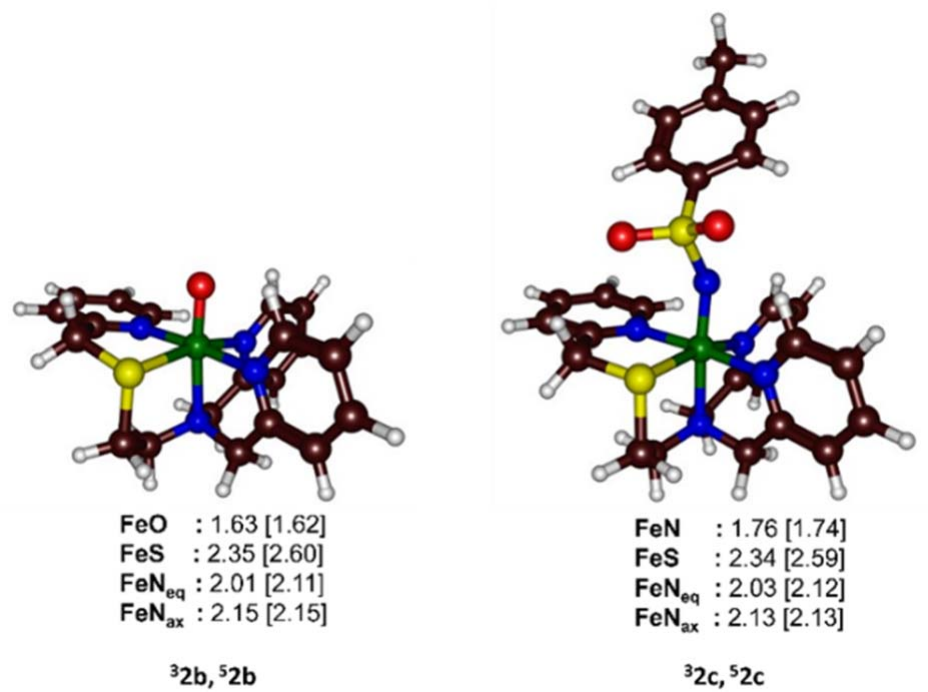
UB3LYP/BS1 optimized geometries in a dielectric constant mimicking acetonitrile of 2b and 2c with bond lengths in Å. FeN_eq._ is the average value of the three equatorial Fe–N bonds and FeN_ax._ is the axial Fe–N bond length.

To gain deeper insights into the differences in reactivity between 2b and 2c, we explored heteroatom transfer and hydrogen atom abstraction processes using density functional theory (DFT) methods. The reaction mechanism for heteroatom transfer was investigated using dimethylsulfide (DMS) as the model substrate, while 1,4-cyclohexadiene (CHD) was used to study hydrogen abstraction and substrate desaturation reactions. The optimized geometries of [Fe^IV^(O)(STPeN)]^2+^ (2b) and [Fe^IV^(NTs)(STPeN)]^2+^ (2c) are shown in Fig. 5 and depict the two lowest-lying spin state structures: triplet and quintet. For both systems we also considered structural isomers of the ligated pentadentate ligands, see the SI; however, similar to the early computational work of Que *et al.* on BnTPeN,^94^ these alternative isomers are quite high in energy also for the STPeN complexes. Thereafter, the triplet and quintet spin state structures of the lowest energy isomer were considered for exploring the underlying mechanism with the substrate. The bond lengths of the metal with the STPeN ligand atoms in the two complexes are very similar to analogous iron(iv)-oxo and iron(iv)-tosylimido systems.^44,45,93^ The Fe–O distance is short (1.63 Å for ^3^2b), while the Fe−NTs distance is much longer: 1.76 Å in ^3^2c. The calculated Fe–O distance of ^3^2b is in good agreement with the value obtained from EXAFS spectroscopy and reported above in Fig. 3d. Typically, iron(iv)-oxo complexes have Fe–O bond lengths ranging from 1.60 to 1.67 Å.^106–121^ In contrast, the Fe–N bonds in iron-tosylimido structures are longer as indeed observed here.^44,45,68–70^ Interestingly, the equatorial and axial ligand distances from the metal are virtually the same for ^3^2b/^3^2c and ^5^2b/^5^2c. For both oxidants, the triplet spin state is the ground state. The triplet state for complex 2b is approximately 5 kcal mol^−1^ lower in energy than its nearest quintet state, while for complex 2c, it is about 3 kcal mol^−1^ lower. The triplet–quintet gap (Δ*E*_TQ_) is lower for Fe^IV^=NTs complexes, which may affect electron affinities and reactivities. The electronic configuration of the triplet spin state for both oxidants is 
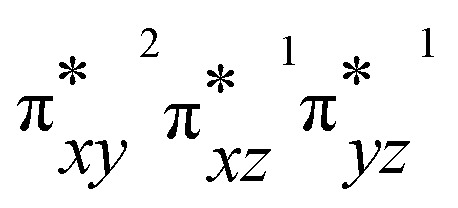
 with four electrons being involved in the interactions of the 3d orbitals with 2p orbitals on oxygen. However, the NTs and oxo groups interact differently with the metal, which alters the orbital energies and some of the interactions. The differences in orbital shapes and energies are displayed in Fig. S28 (SI) for the two complexes. However, the general pictures of the valence orbitals remain the same and hence only minor differences in reactivity are expected. The detailed reaction mechanisms with DMS and CHD are discussed below in separate sections.

### Calculated reaction mechanisms

**Fig. 6:**
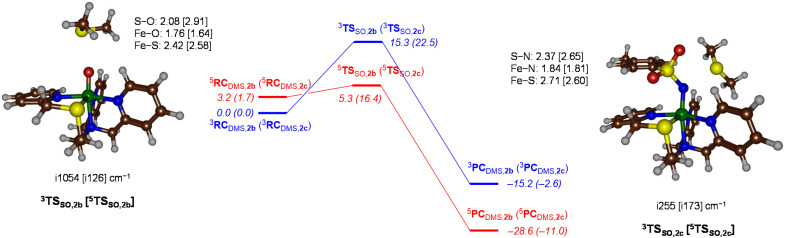
UB3LYP/BS1//UB3LYP/BS2 calculated free energy landscape for DMS sulfoxidation/sulfimidation by 2b and 2c (data for 2c in parenthesis). Free energies (in kcal mol^−1^) calculated at 298 K with zero-point, thermal and entropic corrections at UB3LYP/BS1 level of theory. Optimized transition state structures give distances in Å and the imaginary frequency in cm^−1^.

A detailed investigation into substrate sulfoxidation and sulfimidation reactions was conducted using DMS, with complexes 2b and 2c, respectively. The corresponding potential energy profiles are shown in Fig. 6. Since both sulfoxidation and sulfimidation are single-step reactions, they proceed through the formation of a reactant complex (RC) followed by the transfer of the heteroatom in the transition state (TS), leading to the formation of the product complex (PC). For both systems the substrate reacts concertedly with the oxidant to incorporate an oxygen or nitrogen atom into the sulfide. The reactant complexes exhibit a triplet ground state, but during the reaction pathway, a spin crossover occurs, causing the reaction to shift to a quintet spin state, which presents a lower energy barrier. For the sulfoxidation reaction using iron(iv)-oxo complexes, a low barrier of Δ*G*^‡^ = 5.3 kcal mol^−1^ on the quintet spin surface was found. In contrast, for the sulfimidation reaction with the NTs system, the barrier observed was Δ*G*^‡^ = 16.4 kcal mol^−1^. The higher absolute values of Gibbs free energy for the sulfimidation reaction suggest that DMS will react more quickly with [Fe^IV^(O)(STPeN)]^2+^ (2b) than with [Fe^IV^(NTs)(STPeN)]^2+^ (2c). These findings are consistent with the experimental results, which also demonstrate a faster reactivity of 2b compared to 2c in heteroatom transfer reactions. The transition state structures shown in Fig. 6 exhibit a longer bond distance of 2.91 Å for the substrate oxo interaction (S–O distance) in ^5^TS1_SO,2b_ as compared to a distance of 2.65 Å for the S–N distance between the substrate and the tosylimido group in ^5^TS1_SO,2c_. The imaginary frequency for the transition state ^5^TS_SO,2b_ was found to be i126 cm^−1^, while for ^5^TS_SO,2c_, it was i173 cm^−1^. Therefore, despite the differences in optimized geometries, their imaginary frequencies display a similar mode and magnitude. These imaginary frequencies and geometries of the transition state structure are consistent with previously reported results on substrate sulfoxidation and sulfimidation.^44,45,122,123^ Additionally, these findings align with the DFT results previously obtained on the [Fe(O)(BnTPeN)]^2+^ and [Fe(NTs)(BnTPeN)]^2+^ systems, where NTs was shown to be a less effective oxidant compared to its oxo counterpart.^45,63,92^

**Fig. 7:**
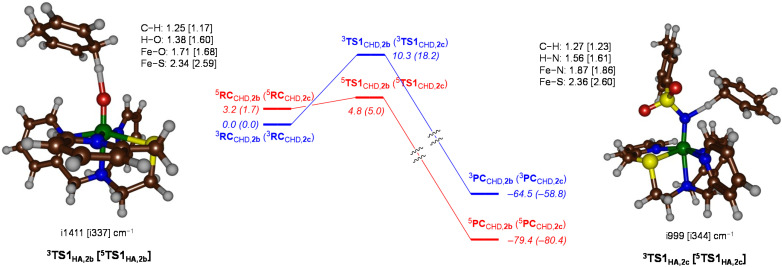
UB3LYP/BS1//UB3LYP/BS2 calculated free energy landscape for CHD dehydrogenation by 2b and 2c (data for 2c in parenthesis). Free energies (in kcal mol^−1^) calculated at 298 K and contain zero-point, thermal and entropic corrections at the UB3LYP/BS1 level of theory. Optimized transition state structures give distances in Å and the imaginary frequency in cm^−1^.

The dehydrogenation reaction mechanism of CHD using ^3,5^2b and ^3,5^2c was subsequently investigated, and the differences in reactivity explored are shown in Fig. 7. The reaction of ^3,5^2b occurs stepwise with an initial hydrogen atom transfer through TS1_H1,2b_ to form a radical intermediate INT_2b_. This is followed by the second hydrogen abstraction *via*TS2_H1,2b_, resulting in the formation of benzene as the dehydrogenated product. The abstraction of the first hydrogen atom by the complex 2b is the rate-determining step of the reaction, with a relative Gibbs free energy (Δ*G*) of 10.3 kcal mol^−1^ on the triplet spin surface and 4.8 kcal mol^−1^ on the quintet spin surface, as illustrated in Fig. 7. This indicates that a spin crossover occurs during the transition state. The transition states for both spin states have a radical character reminiscent of the typical H-abstraction mechanism. The formation of an intermediate (^3^INT_2b_, ^5^INT_2b_) is exothermic for both spin states, with exothermicities of −18.3 and −30.7 kcal mol^−1^ on the triplet and quintet spin states, respectively. The large energy difference between these two radical intermediates is the result of a hydrogen atom transfer happening in the triplet spin state, whereas in the quintet spin state a hydride transfer occurs. The second hydrogen atom abstraction *via*^3^TS2_H1,2b_ occurs with a marginal barrier of ∼3 kcal mol^−1^ for the triplet spin state. In contrast, facile abstraction of a proton is observed for the quintet spin state to generate benzene as the product. The product formation is exothermic by nearly −80 kcal mol^−1^ for the quintet spin state. This suggests that the reaction for the oxo complexes occurs predominantly on the quintet spin surface after a spin crossover just before the hydrogen abstraction transition state.

The imaginary frequencies for the hydrogen atom abstraction transition state are i999 and i344 cm^−1^ for the triplet and quintet spin states of the TS1_HA,2c_ complexes, respectively, and represent a C–H–O stretching vibration. Similar to 2b, the spin crossover in 2c occurs close to the first transition state. The difference in Gibbs free energy between ^5^TS1_HA,2b_ and ^5^TS1_HA,2c_ is only 0.2 kcal mol^−1^, which indicates that both complexes should show similar activity for C–H abstraction processes. Indeed, experimental results show that the 2b and 2c complexes exhibit comparable reactivity with CHD, in good agreement with the calculated energy landscape. The intermediate formation is exothermic for intermediates ^3,5^INT_2c_ with energies −17.0 (triplet) and −43.4 kcal mol^−1^ (quintet). The intermediate on the quintet spin state surface is highly stable compared to the triplet. To understand the origin of the high stability of ^5^INT_2c_ the group spin densities of ^3,5^INT_2c_ were examined and compared with those of the ^3,5^INT_2b_ complexes. The intermediates ^3^INT_2b,2c_ are radical intermediates with one unpaired electron on the substrate due to single electron transfer from the substrate to the metal centre. Thus, the hydrogen atom transfer (HAT) mechanism occurs in the triplet spin state for both 2b and 2c. In the quintet spin state of ^5^INT_2b__,_ the substrate exhibits a spin density of −0.88, consistent with the electronic configuration of 
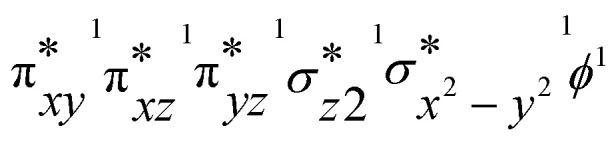
. In contrast, the substrate moiety in ^5^INT_2c_ has a total spin of 0.0, indicating a hydride transfer, which involves the transfer of a proton and two electrons. This intermediate has an electronic configuration that resembles the product complex, featuring four metal-based orbitals that are singly occupied. The configuration is represented as 
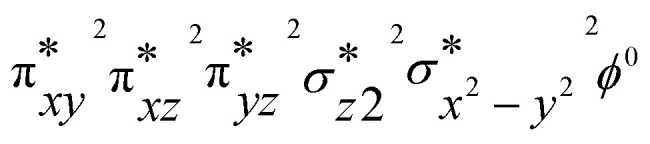
. Hence, the intermediate ^5^INT_2c_ is highly stable due to a product-like electronic configuration. The second hydrogen abstraction occurs concertedly on a quintet spin state surface, resulting in product formation with high exothermicity. It is evident from the results that the mechanism of the reaction between CHD and 2b differs from that of 2c. Therefore, for 2b the dehydrogenation reaction occurs through a HAT-type mechanism, whereas the corresponding reaction for 2c involves hydride transfer followed by proton transfer. These results align well with the experimental Hammett values, indicating that the iron(iv)-imido system follows an electron transfer mechanism. This result is similar to our earlier investigation of the N_5_ and N_4_S systems of the TACN scaffold, where a switch in the mechanism was observed for the N_4_S iron(iv)-oxo complex.^93^ The reaction of DMS is slower with iron(iv)-imido as compared to the reaction with iron(iv)-oxo, but has a lower free energy of activation than [Fe^IV^(O)(BnTPeN)]^2+^ by nearly 3 kal mol^−1^, but comparable reactivity is observed between [Fe^IV^(O)(STPeN)]^2+^ and [Fe^IV^(NTS)(STPeN)]^2+^ for dehydrogenation. Hence, the STPeN system demonstrates enhanced reactivity for both iron(iv)-oxo and iron(iv)-imido complexes compared to BnTPeN analogues, as also observed experimentally.

**Fig. 8:**
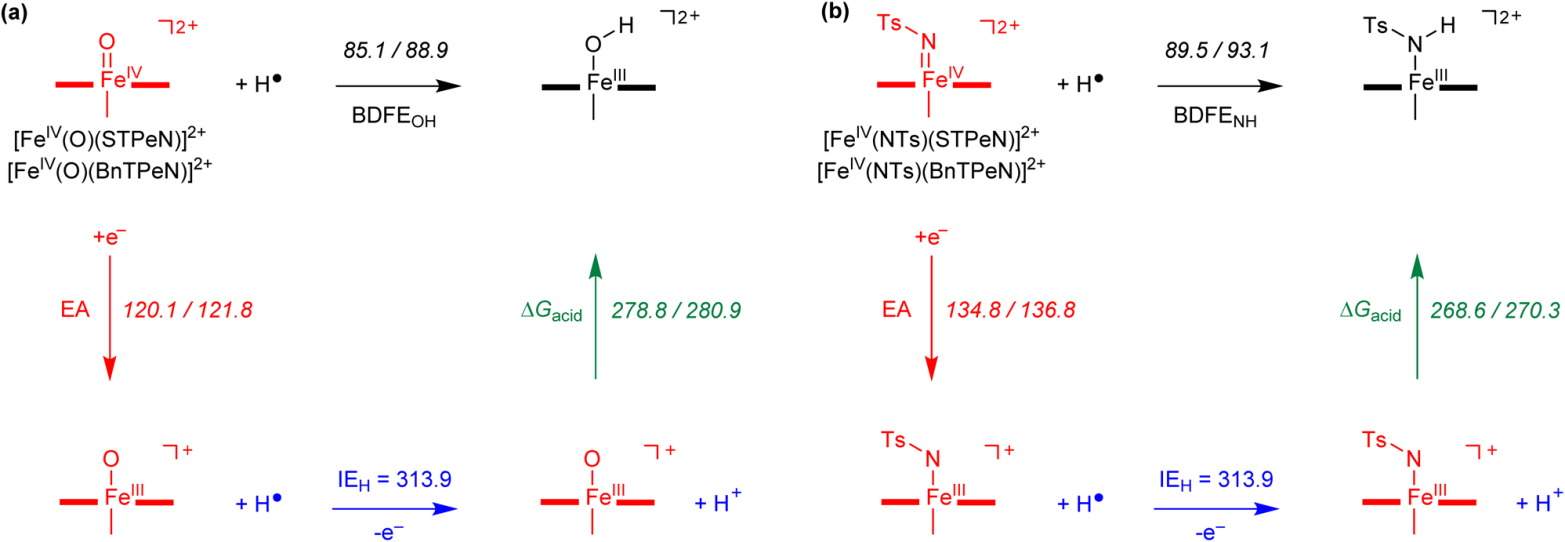
UB3LYP-GD3BJ/BS2 calculated thermochemical variables for adiabatic transitions. Data represent free energies (in kcal mol^−1^) calculated at 298 K and contain zero-point, thermal, solvent and entropic corrections. (a) Data for iron(iv)-oxo complexes [Fe^IV^(O)(STPeN)]^2+^ (2b)/[Fe^IV^(O)(BnTPeN)]^2+^ (1b). (b) Data for iron(iv)-tosylimido complexes [Fe^IV^(NTs)(STPeN)]^2+^ (2c)/[Fe^IV^(NTs)(BnTPeN)]^2+^ (1c).

To understand the reactivity differences of the various iron(iv)-oxo and iron(iv)-tosylimido complexes with BnTPeN and STPeN ligand frameworks, we decided to calculate a series of thermochemical properties. Thus, previous work showed that often reactivities follow trends in the strength of the formation of the O–H bond after hydrogen atom abstraction, *i.e.*, the bond dissociation free energy (BDFE_OH_).^123,124^ However, work on [Fe^IV^(O)(N4Py)]^2+^*versus* [Fe^IV^(NTs)(N4Py)]^2+^ showed that the latter had a much larger electron affinity (EA) for the one-electron reduction of the complex that caused the enhanced reactivity with sulfides.^44,45^ The BDFE and EA values were calculated for complexes 1b, 1c, 2b and 2c and the results are shown in Fig. 8. The electron affinity represents the adiabatic value between the lowest energy spin state of the reactant complex and the one-electron reduced conformer. For the BDFE values the energy difference between the reactant complex, an isolated hydrogen atom and the iron(iii)-hydroxo or iron(iii)–N(H)Ts complexes is taken into consideration. Using the calculated BDFE and EA values together with the experimental value of the ionization energy of a hydrogen atom from the NIST database,^125^ we evaluated the gas-phase acidity of the complexes (Δ*G*_acid_). In general, the iron(iv)-tosylimido complexes have an electron affinity that is higher by almost 15 kcal mol^−1^ with respect to the iron(iv)-oxo species for both the STPeN and BnTPeN-ligated systems, which matches the trends reported previously for the N4Py-ligated systems.^45^ A larger electron affinity indicates a more exothermic electron transfer, which agrees with the experimental and computational mechanistic results which elucidate that electron transfer happens fast and early. Interestingly, both the iron(iv)-oxo and iron(iv)-tosylimido complexes with the BnTPeN ligand system have a larger BDFE than the corresponding complexes with the STPeN-ligand. This implies that the BnTPeN-ligated system reacts with more exothermicity for hydrogen atom abstraction than the corresponding STPeN-ligated complexes, although this advantage is mostly cancelled out by the enlarged electron affinity so that the gas phase basicity is almost the same for the two complexes. In previous studies on tetramethyl-cyclam (TMC)-ligated iron(iv)-oxo complexes the substitution of one of the nitrogen atoms in the ligand framework by a sulfur atom led to a trend in hydrogen atom abstraction barriers that correlated with the HOMO–LUMO energy gap.^71^ Of course, the HOMO–LUMO gap is connected to the electron affinity of the complex and implies that electron transfer is quicker and earlier when a sulfur atom is included in the ligand framework. In the case of 1b*versus*2b and 1c*versus*2c, however, very similar electron affinities are calculated, which implies that for our systems the reactivity differences will not be determined by the HOMO–LUMO gap or EA differences, but, as shown in Fig. 8, mostly through the formation of a stronger O–H/N–H bond in the complex after hydrogen atom abstraction.

## Conclusions

In summary, this report presents the experimental characterization of a sulfur-ligated iron(iv)-tosylimido complex as a mimic of a potential short-lived catalytic cycle intermediate of nitrogenase. The iron(iv)-tosylimido complex was spectroscopically well characterized and compared with its iron(iv)-oxo counterpart using UV-vis absorption spectroscopy, ESI-MS, XANES, EXAFS and resonance Raman spectroscopy. Also, the reactivity studies in heteroatom oxidation and C–H activation reactions were studied for both complexes. In both heteroatom oxidation and C–H activation reactions, the iron(iv)-oxo complex is identified as more reactive than the iron(iv)-tosylimido species. The mechanistic pathways for *S*-oxidation reactions for the corresponding iron(iv)-oxo and iron(iv)-tosylimido complexes are, however, different. The former proceeds *via* oxygen atom transfer while in the latter case electron transfer is the rate-limiting step.

## Author contributions

JKS, CVS, and SPdV conceptualized and designed the research, JKS and RY performed data acquisition and analysis alongside LS, and the results were interpreted by JKS, RY, CVS and SPdV. EXAFS data acquisition and interpretation were carried out by JU & EN. The manuscript was prepared jointly by JKS, RY, LS, JU, EN, SPdV and CVS. All authors have approved the final version of the manuscript.

## Conflicts of interest

There are no conflicts of interest to declare.

## Supplementary Material

SC-017-D5SC07586F-s001

SC-017-D5SC07586F-s002

## Data Availability

The data supporting this article have been included as part of the supplementary information (SI). Supplementary information is available. See DOI: https://doi.org/10.1039/d5sc07586f.
