# Supplementary material for: Sulfur-ligated iron(iv)-imido and iron(iv)-oxo complexes, which one is more reactive?
Source: Chem Sci. 2025 Nov 17;17(2):1349–64. doi: 10.1039/d5sc07586f (PMC12649796; doi:10.1039/d5sc07586f)
Supplement: SC-017-D5SC07586F-s001 [file SC-017-D5SC07586F-s001.pdf]

Supplementary Information (SI) for Chemical Science.
This journal is © The Royal Society of Chemistry 2025

Electronic Supporting Information

Sulfur-Ligated Iron(IV)-Imido and Iron(IV)-Oxo Complexes, Which One Is More Reactive?

Jagnyesh Kumar Satpathy,[a] Rolly Yadav,[a†] Limashree Sahoo,[a] Jens Uhlig,[b] Ebbe Nordlander,[b] Chivukula V. Sastri,*[a] and Sam P. de Visser,*[a,c,d]

[a] Department of Chemistry, Indian Institute of Technology Guwahati, Assam, 781039, India.
[b] Department of Chemical Physics, University of Lund, Lund, Sweden
[c] Manchester Institute of Biotechnology, The University of Manchester, 131 Princess Street, Manchester M1 7DN, United Kingdom. E-mail: sam.devisser@manchester.ac.uk.
[d] Department of Chemical Engineering, The University of Manchester, Oxford Road, Manchester M13 9PL, United Kingdom. E-mail: sam.devisser@manchester.ac.uk.

Table of Contents:

**Part I: Experimental procedures.**	**Page S3**
**Part II: Experimental characterization.**	**Page S5**
**Part III: Computational data.**	**Page S22**
**Part IV: Cartesian coordinates.**	**Page S28**

Part I: Experimental procedures.

Synthesis of Ligand

The ligand BnTPeN was synthesized by following a previously reported procedure,[1] while the ligand STPeNwas synthesized based on a reported procedure,[2,3] but with small alterations as shown in Scheme S1. Thus, 2-aminoethanethiol hydrochloride (20 mmol) and 2-(chloromethyl)pyridine hydrochloride (10.1 mmol) were added to a solution of NaOH (40.2 mmol) in EtOH (20 mL) with ice bath cooling. The reaction mixture was stirred for 30 min before the ice bath was removed and, thereafter, the stirring of the mixture was continued at room temperature for 2.5 h. The EtOH was removed under reduced pressure and water (25 mL) was added to the resulting residue. The aqueous solution was extracted with CH_2Cl_2 (3 x 25 mL) and the combined organic layer washed with brine (10 mL), dried (with K_2CO_3), filtered, and the solvent removed in vacuo to afford a crude yellow oil. Column chromatography (SiO_2, CH_2Cl_2 : MeOH : NH_4OH, 9 : 2 : 0.2) produced compound **1** as a yellow oil.

To a solution of **1** (20 mmol) in MeOH (200 mL) was added 2-pyridinealdehyde (20 mmol). After the solution was stirred at room temp. for 2 h, $NaBH_4$ (20 mmol) was added in several portions and the resulting solution was stirred at room temperature for 1 h. The solvent was removed under reduced pressure. The residue was extracted with $CHCl_3$ and washed with water. The organic phase was dried with Na_2SO_4. After filtration, the solvent was removed under reduced pressure to afford **2** as pale brown oil.

To a solution of 2-(chloromethyl)pyridinehydrochloride (10 mmol) in EtOH (10 mL) was added **2** (10 mmol) and $NaHCO_3$ (100 mmol). After the mixture was stirred at 60 °C for 6 days, the resultant solution was filtered and dried in vacuo. The residue was extracted with $CHCl_3$ and washed with water. The organic phase was dried with Na_2SO_4. After filtration, the solvent was removed under reduced pressure and the crude product was purified with silica gel column chromatography ($CHCl_3$/MeOH = 95:5) to give dark brown oil L_2.

^{1}H NMR (500 MHz, $CDCl_3$) δ 8.51 (d, J = 5.0 Hz, 2H), 7.68–7.64 (m, 2H), 7.52 (d, J = 7.5 Hz, 2H), 7.16–7.13 (m, 2H), 3.85 (s, 4H), 3.11 (t, J = 5.0 Hz, 4H), 2.95 (t, J = 5.0 Hz, 4H), 2.68 (s, 4H); ^{13}C NMR (125 MHz, CDCl3) δ 160.2, 159.2, 149.3, 148.8, 136.8, 136.5, 123.5, 122.5, 122.2, 120.7, 64.6, 64.4, 59.3, 56.3, 32.3, *m/z*: 329 (328 + H^+)

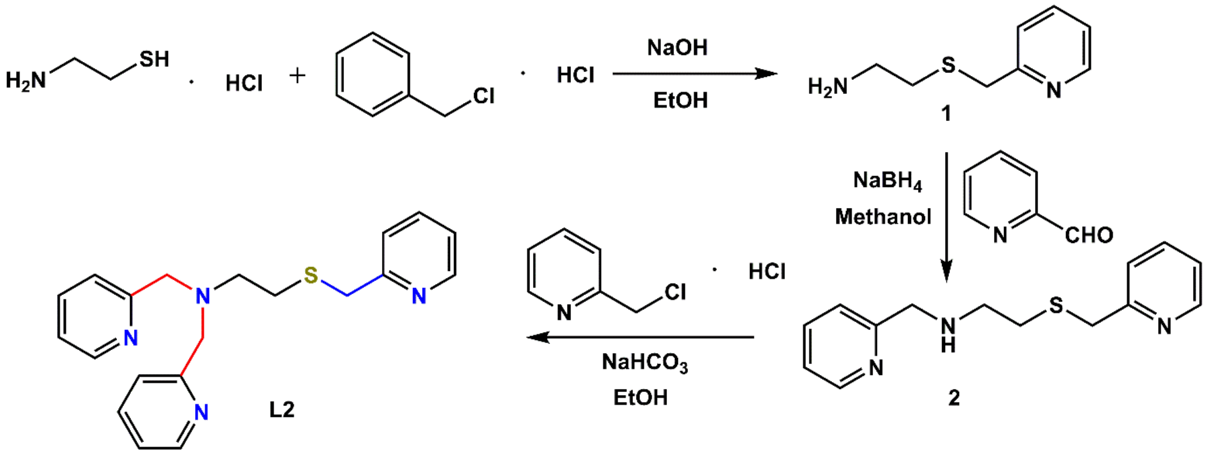

Scheme S1. Synthesis of the STPeN ligand.

Synthesis of metal complexes

The metal complex [Fe^{II}(BnTPeN)(OTf)](OTf) (**1a**) was synthesized by following the reported procedure.[1] [Fe^{II}(STPeN)(OTf)](OTf) (**2a**) was synthesized using the following procedure: 100 mg ligand L_2 was put into a glass vial and dissolved in 2 mL acetonitrile inside a glove box. To this solution, 1.2 eq. of [Fe^{II}(CF_3SO_3)$_2$]•2CH_3CN in 2 mL of acetonitrile were added and stirred overnight at room temperature. The resulting reddish solution was filtered, layered by diethyl ether and kept at –40 °C for slow vapor diffusion. After few days reddish brown crystals are seen. The solution was decanted and dried under vacuum to obtain the desired metal complex **2a** in >90% yield. Complex **1a** and **2a** were characterized by ESI-MS, Cyclic-Voltametry and UV/Vis spectroscopy (Fig. S5 – S7, Tables S1 and S2).

References

1. J. Kaizer, E. J. Klinker, N. Y. Oh, J.-U. Rohde, W. J. Song, A. Stubna, J. Kim, E. Münck, W. Nam and L. Que Jr, Nonheme $Fe^{IV}O$ Complexes That Can Oxidize the C−H Bonds of Cyclohexane at Room Temperature. *J. Am. Chem. Soc.*, 2004, **126**, 472.

2. T. Nakajima, Y. Kawasaki, B. Kure and T. Tanase, Homo- and Heterodinuclear Rh and Ir Complexes Supported by SN_n Mixed-Donor Ligands (n = 2–4): Stereochemistry and Coordination-Site-Exchange Reactions of Cp*M (M = Rh, Ir) Units. *Eur. J. Inorg. Chem.*, 2016, 4701.

3. S. J. Mountford, R. Daly, A. J. Robinson and M. T. W. Hearn, Design, Synthesis and Evaluation of Pyridine-Based Chromatographic Adsorbents for Antibody Purification. *J. Chromatogr. A*, 2014, **1355**, 15.

Part II: Experimental Compound Characterization.

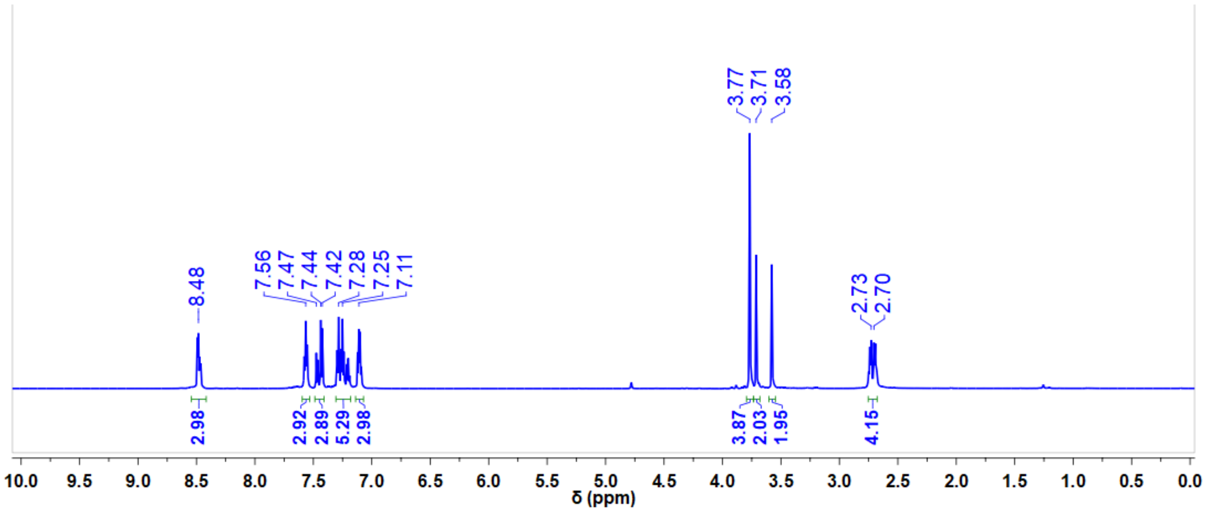

Fig. S1. ^{1}H NMR spectrum of BnTPeN in $CDCl_3$.

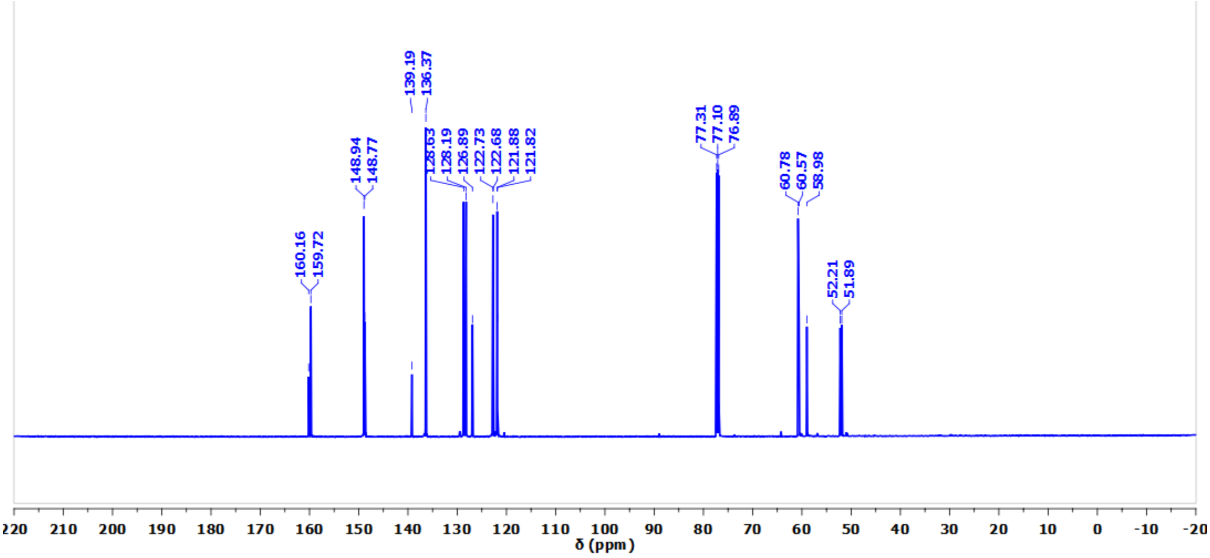

Fig. S2. ^{13}C NMR spectrum of BnTPeN in $CDCl_3$.

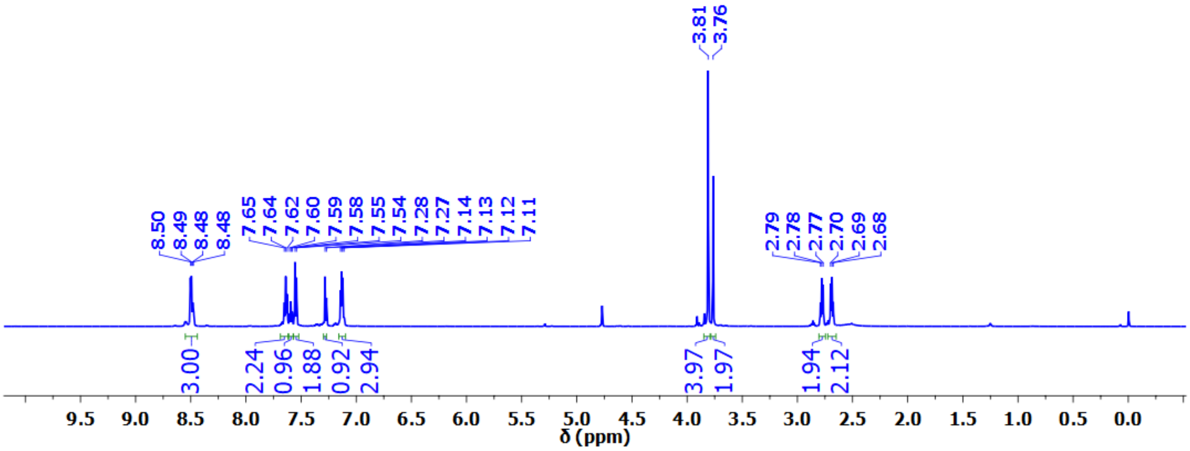

Fig. S3. ^{1}H NMR spectrum of STPeN in $CDCl_3$.

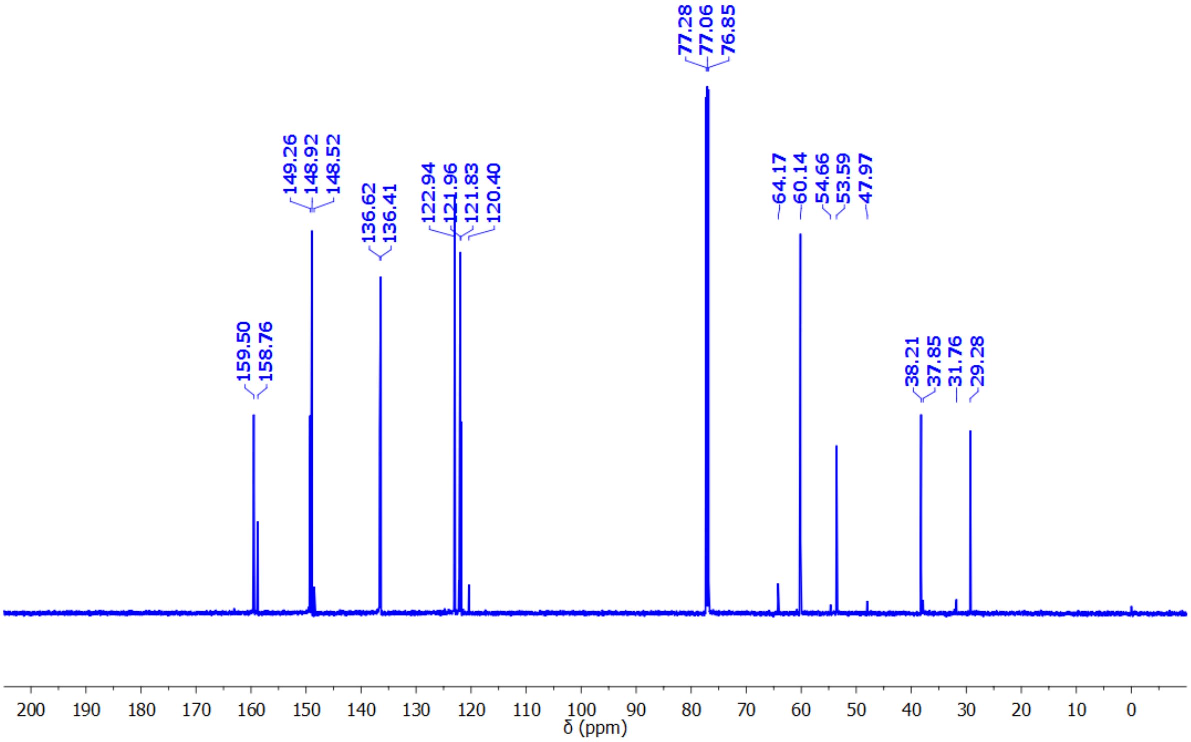

Fig. S4. ^{13}C NMR spectrum of STPeN in $CDCl_3$.

Table S1: Crystallographic data and structure refinement of the complex $[Fe^{II}(STPeN)(CH_3CN)](OTf)_2$.

Crystal identification	*$[Fe^{II}(STPeN)(CH_3CN)](OTf)_2$*
Empirical formula (Calculated)	C_{24} H_{25} F_6 Fe N_5 O_6 S_3
Formula Weight (Calculated) 745.52 g/mol	745.52 g/mol
Color, morphology	Deep red, block
Temperature	298 K
Wavelength	0.71073 Å
Space group (Calculated)	P -1
Hall group	-P 1
Volume (Calculated)	1536 (3) $Å^3$
Bond precision	C-C = 0.0056 Å
Unit cell parameters	a = 11.840 (13) Å α = 109.78 (3) °
	b = 12.227 (13) Å β = 95.38 (4) °
	c = 12.436 (13) Å γ = 110.84 (3) °
Z (calculated)	2
Density (calculated) (g cm^{-3})	1.612
Mu (mm^{-1})	0.779
F000 (F000')	760.0 (761.87)
h, k, l_{max}	14, 15, 15
N ref (calculated)	6482
N ref (Reported)	6333
Minimum transmission (T) (calculated)	0.817 [0.869]
[reported limit]	
Maximum transmission (calculated)	0.660 [0.747]
[reported limit]	
Absorption correction	Multi-scan
Data completeness	0.977
Theta (max)	26.667
R (Reflections)	0.0518 (4158)
wR2 (Reflections)	0.1379 (6333)
R	0.0518 (4158)
S	1.081
Npar	407
Prob (%)	50

Table S2: Selected bond angles for the complex obtained for the crystal structure of $[Fe^{II}(STPeN)(CH_3CN)](OTf)_2$.

cis-equatorial bond angles (°)		*cis*-bond angle of equatorial Fe1-S with $N_{ax/eq}$ (°)	
∠ N1-Fe1-N4	90.05(2)	∠ N3-Fe1-S1	85.50 (2)
∠ N1-Fe1-N3	94.34(2)	∠ N4-Fe1-S1	89.90(2)
∠ N2-Fe1-N4	84.18(2)	∠ N2-Fe1-S1	88.44(2)
∠ S1-Fe1-N3	85.50(2)	∠ N5-Fe1-S1	96.94(2)
***trans*-equatorial bond angles (°)**		***cis*-bond angle of equatorial Fe1-N/ Fe1-S with N2 (°)**	
∠ N3-Fe1-N4	175.38(2)	∠ N1-Fe1-N2	80.84(2)
∠ S1-Fe1-N1	169.23(2)	∠ N4-Fe1-N2	84.18(2)
-	-	∠ N3-Fe1-N2	95.11(2)
-	-	∠ S1-Fe1-N2	88.44(2)

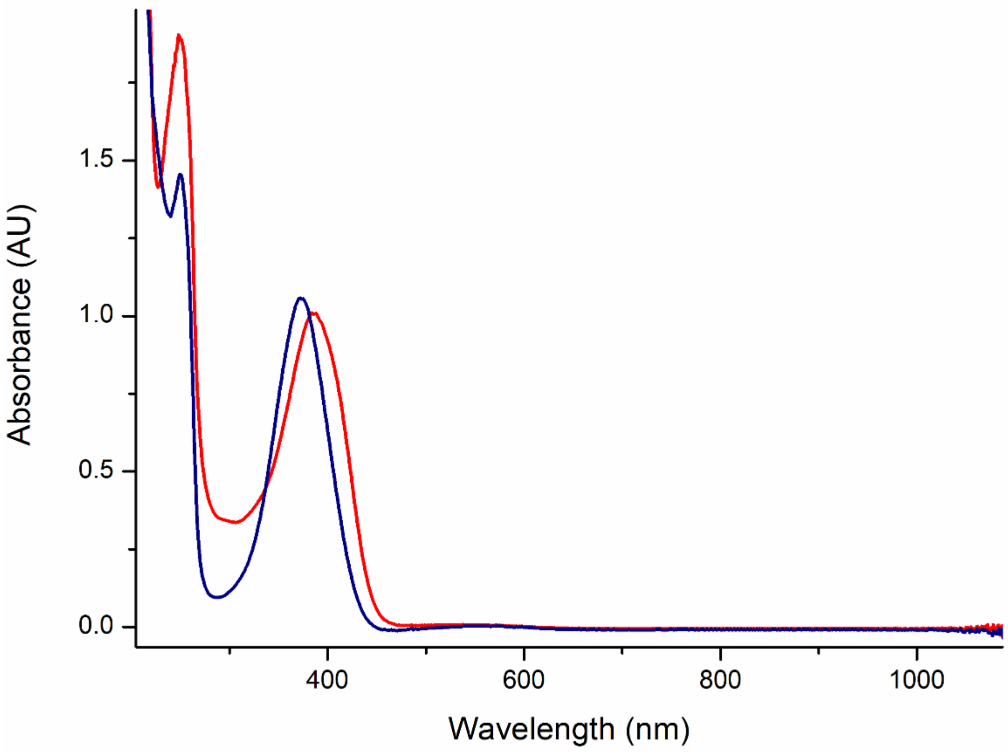

Fig. S5. UV-vis spectra of **1a** (in red) and **2a** (in blue) in CH_3CN at RT.

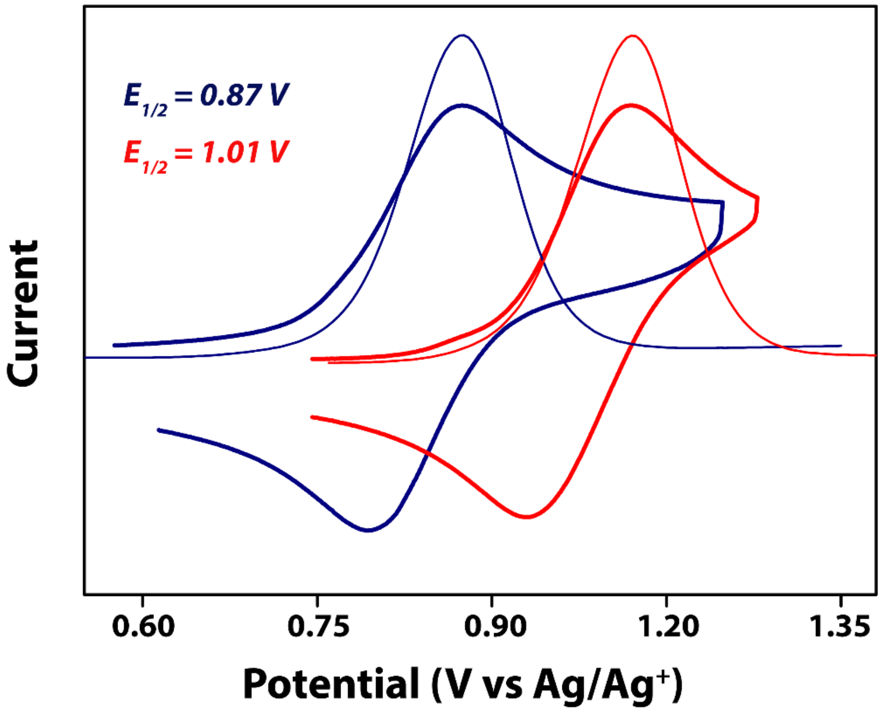

Fig. S6. Cyclic and Differential Pulse Voltammograms for **1a** and **2a** in anhydrous CH_3CN at 25 °C vs Ag/Ag^+.

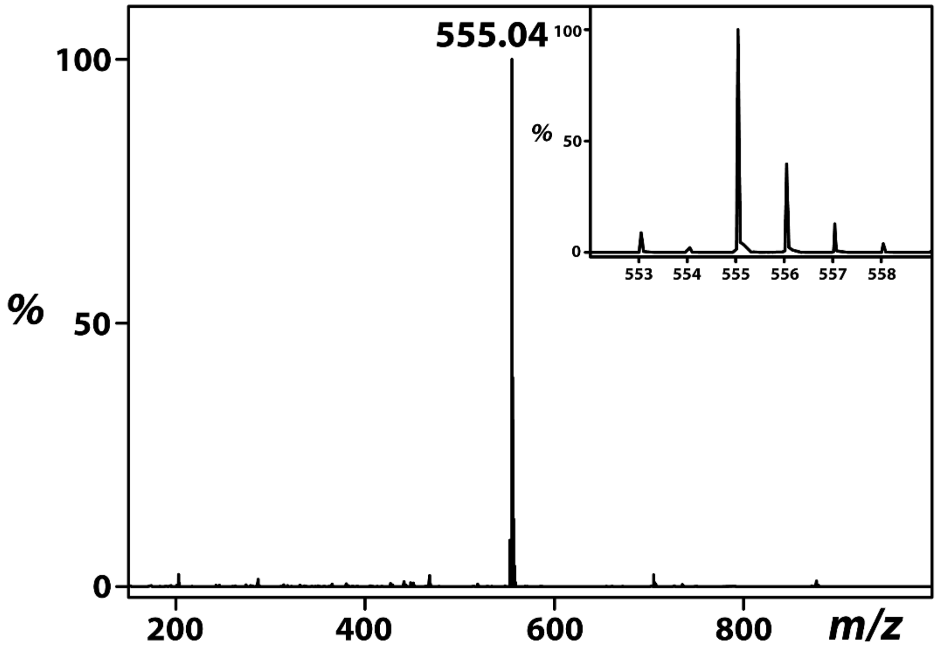

Fig. S7. ESI-MS spectrum of complex **2a** in CH_3CN at 25 °C. The peak at *m/z* 555.04 corresponds to $[Fe^{II}(STPeN)(OTf)]^+$ and the inset shows the isotopic distribution pattern of *m/z* 530.1.

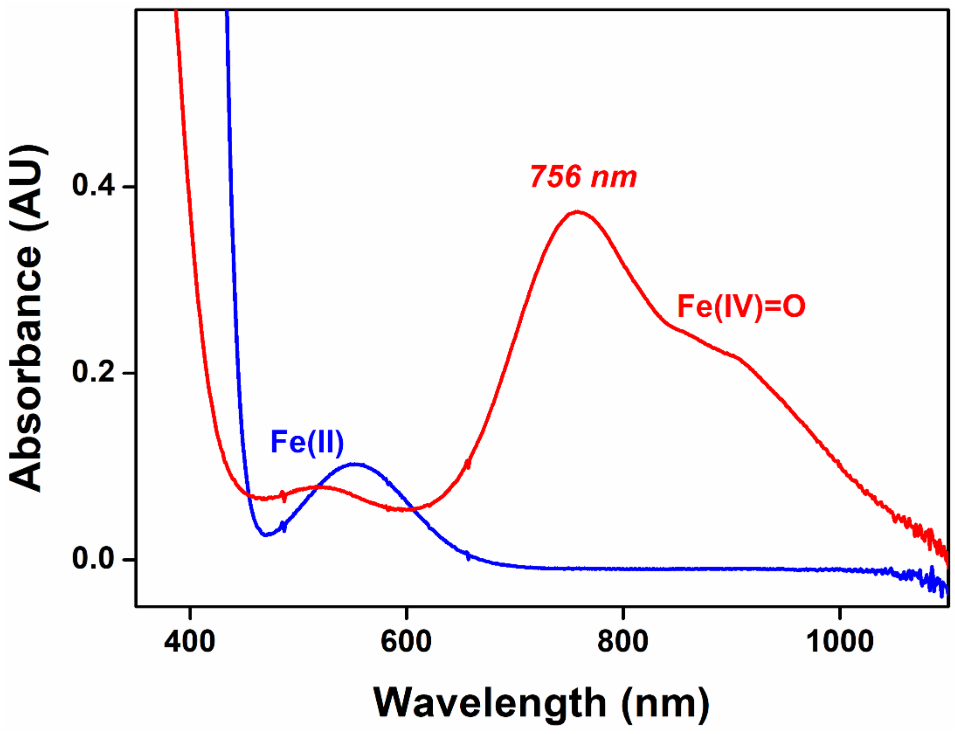

Fig. S8. UV-vis spectra of **2a** before and after the addition of 1.5 equiv. of [s]PhIO to the 1 mM solution of **2a** in acetonitrile at RT.

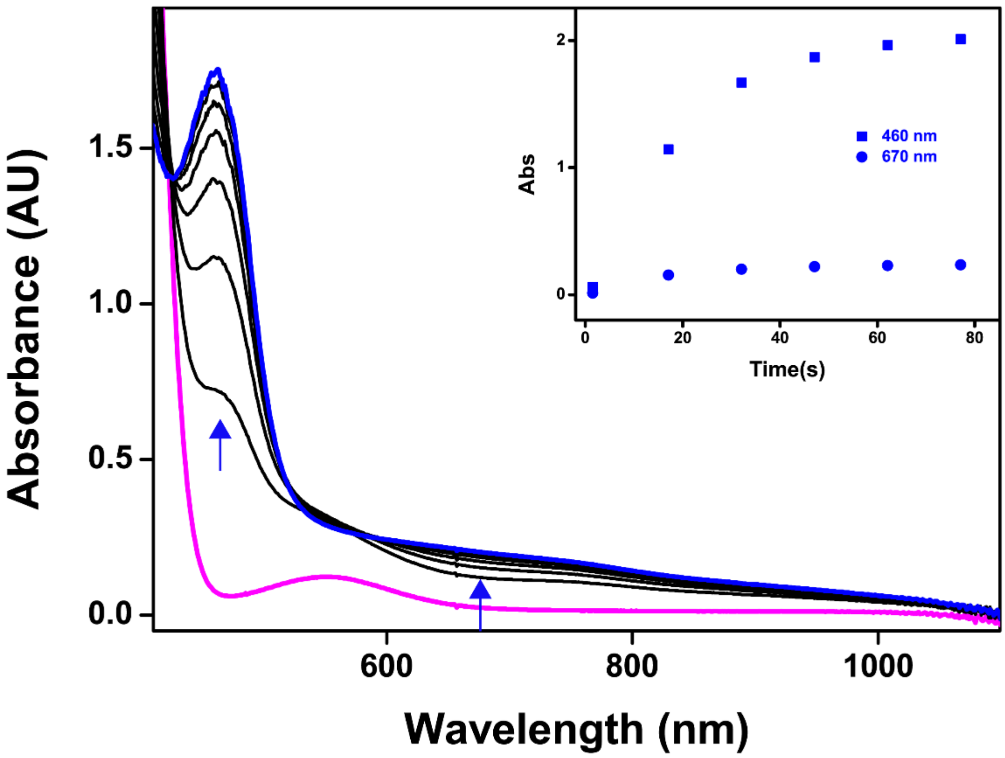

Fig. S9. UV-vis spectral changes observed after the addition of 1.5 equiv. of sPhINTs to the 1 mM solution of **2a** in acetonitrile at RT.

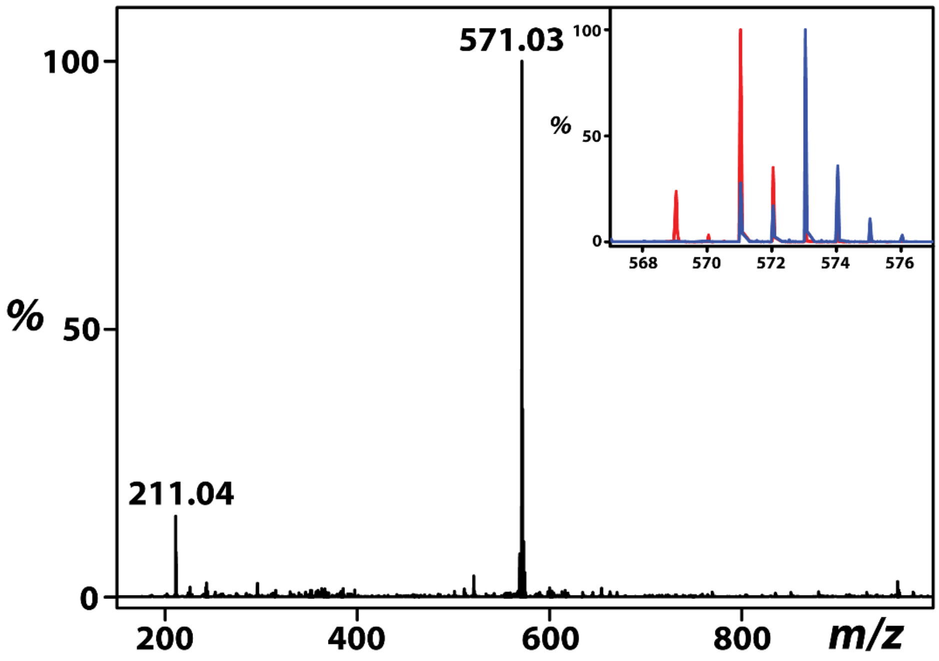

Fig. S10. ESI-MS spectrum of complex **2b** in CH_3CN at 25 °C. The peak at *m/z* 571.03 corresponds to $[Fe^{IV}(O)(STPeN)(OTf)]^+$ and the peak at *m/z* 211.04 corresponds to $[Fe^{IV}(O)(STPeN)]^{2+}$. The inset shows the isotopic distribution pattern of *m/z* 571.03.

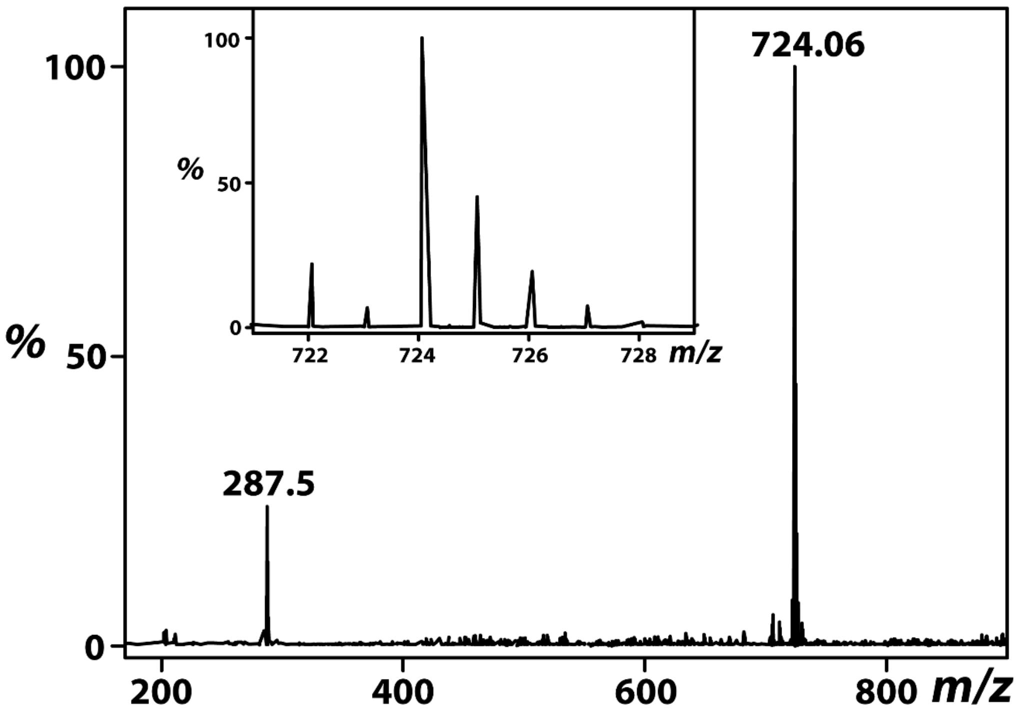

Fig. S11. ESI-MS spectrum of complex **2c** in CH_3CN at 25 °C. The peak at *m/z* 724.06 corresponds to $[Fe^{IV}(NTs)(STPeN)(OTf)]^+$ and the peak at *m/z* 211.04 corresponds to $[Fe^{IV}(NTs)(STPeN)]^{2+}$. The inset shows the isotopic distribution pattern of *m/z* 724.06.

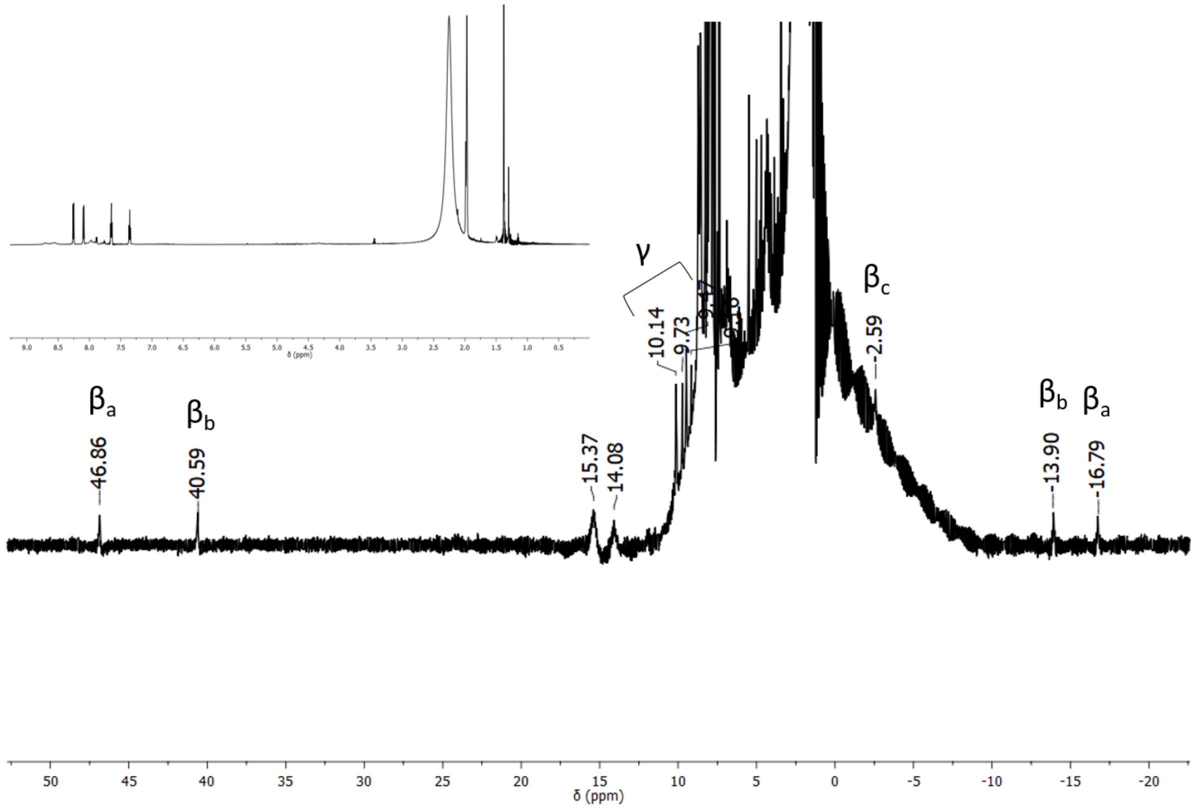

Fig S12. ^{1}H NMR spectra of **2b** in CD_3CN. (256 scans, spectral width= 200 ppm)

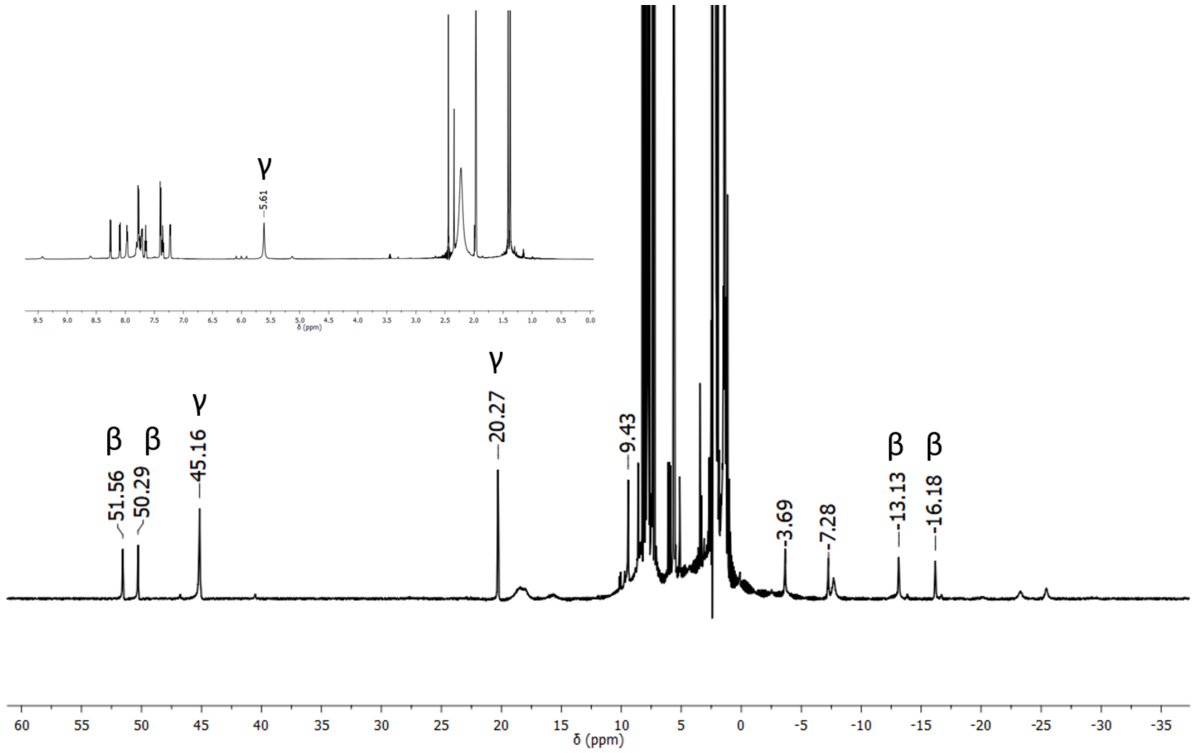

Fig S13. ^{1}H NMR spectra of **2c** in CD_3CN. (256 scans, spectral width= 200 ppm)

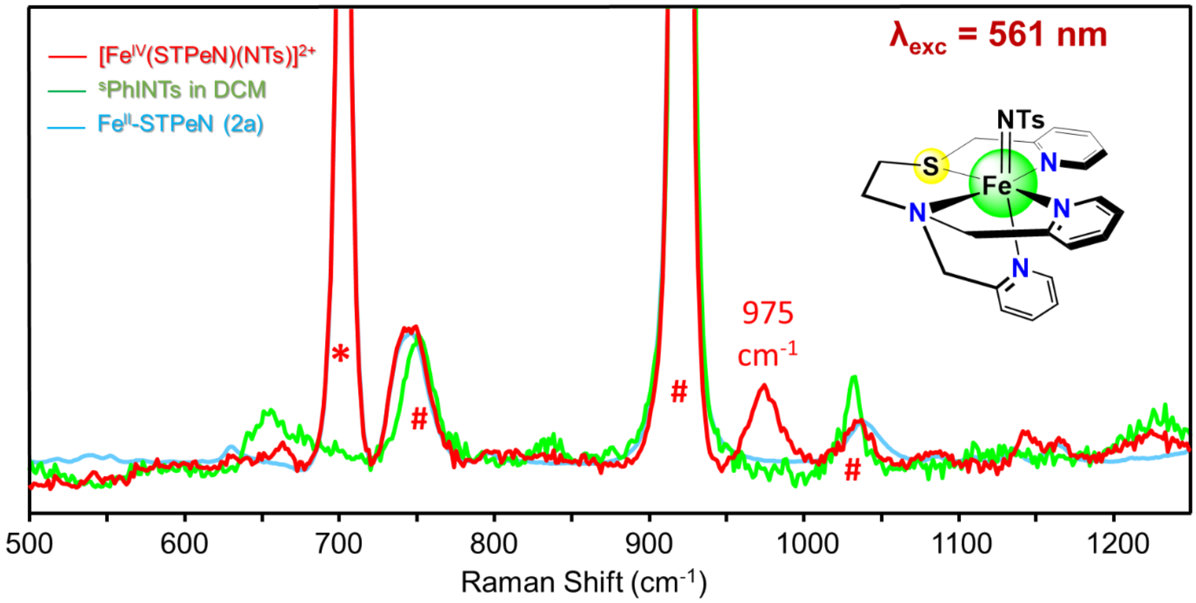

Fig. S14. Resonance Raman spectra of **2c** at 298 K at 561 nm excitation wavelength. The symbols * and # refer to solvent peaks. [Conditions to generate **2c** for rR experiments: 5 mM **2a** + 3 equiv. of ^{s}PhINTs (in dichloromethane) in acetonitrile as solvent.]

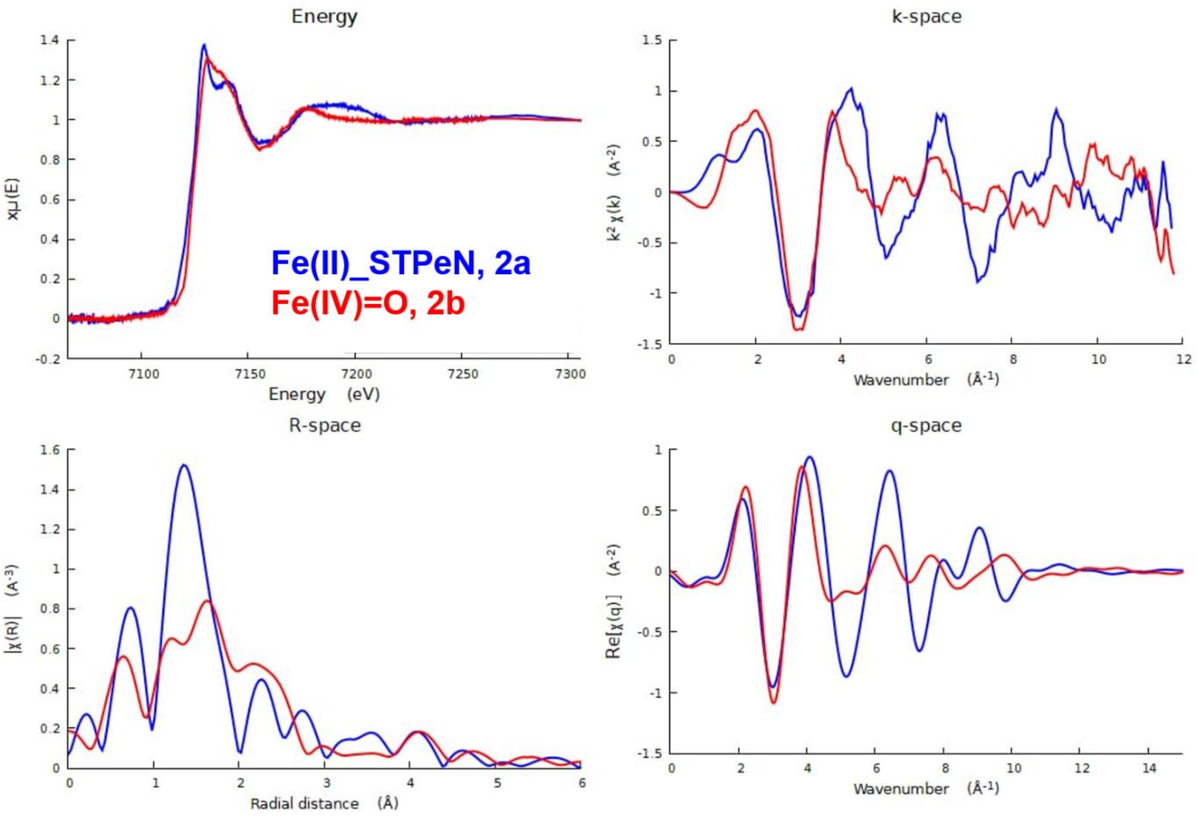

Fig. S15. Normalized Fe K-edge X-ray absorption spectra of **2a** (blue) and **2b** (red) in CH_3CN and Fourier-transformed Fe K-edge EXAFS spectra of **2a** (blue) and **2b** (red) in CH_3CN in K, R and q-Space.

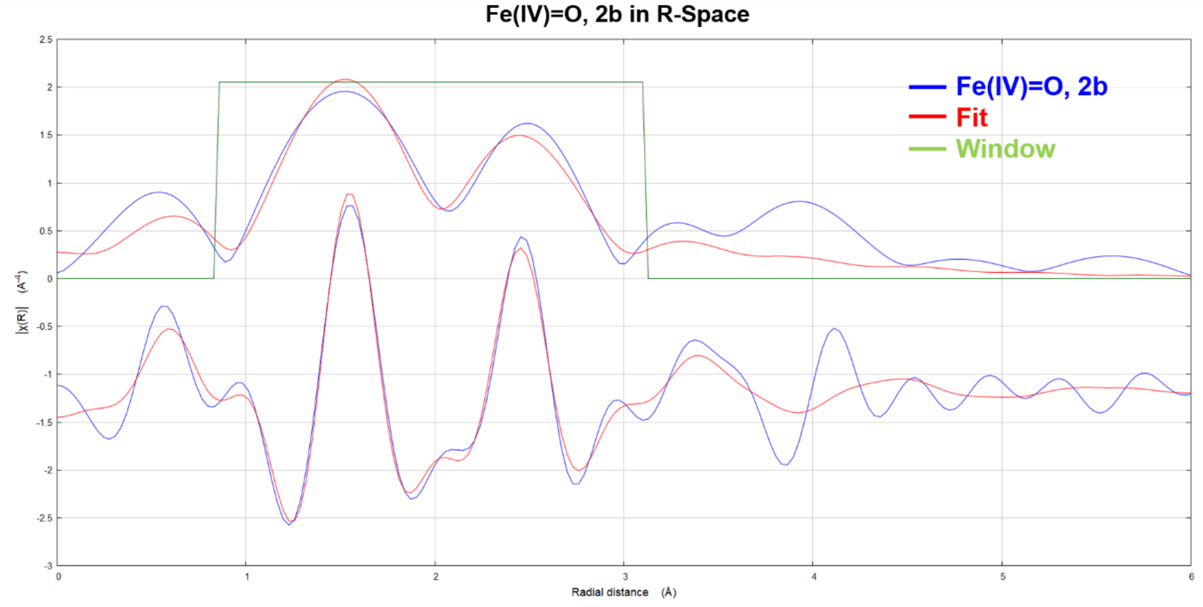

Fig. S16. Unfiltered EXAFS spectra (blue line) and corresponding best fits (red lines) of **2b**.

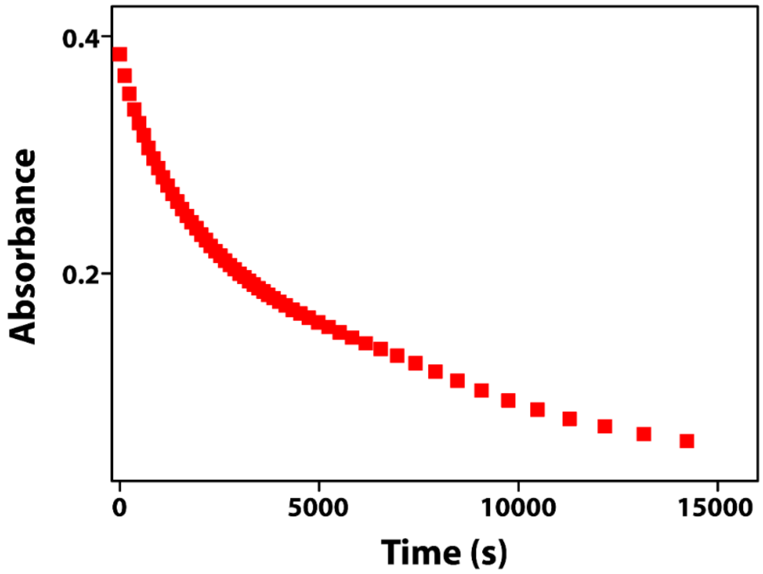

Fig. S17. Decay of **2b** over time in acetonitrile at 298 K.

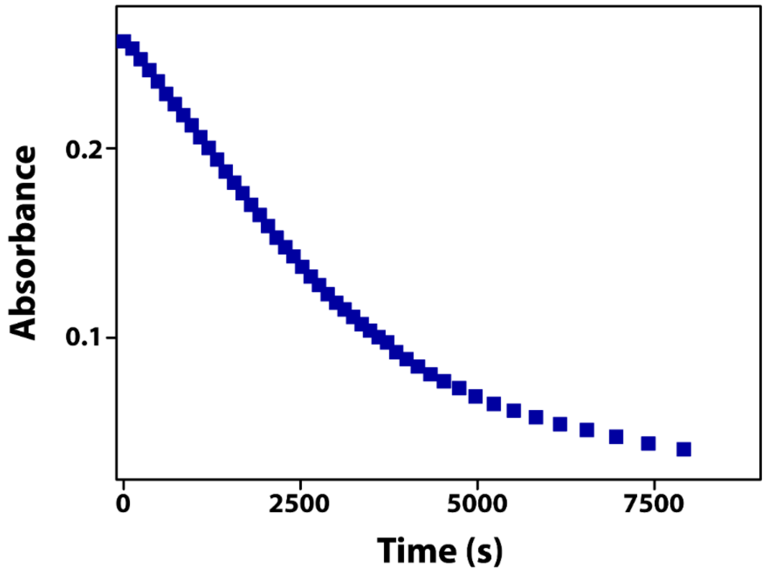

Fig. S18. Decay of **2c** over time in acetonitrile at 298 K.

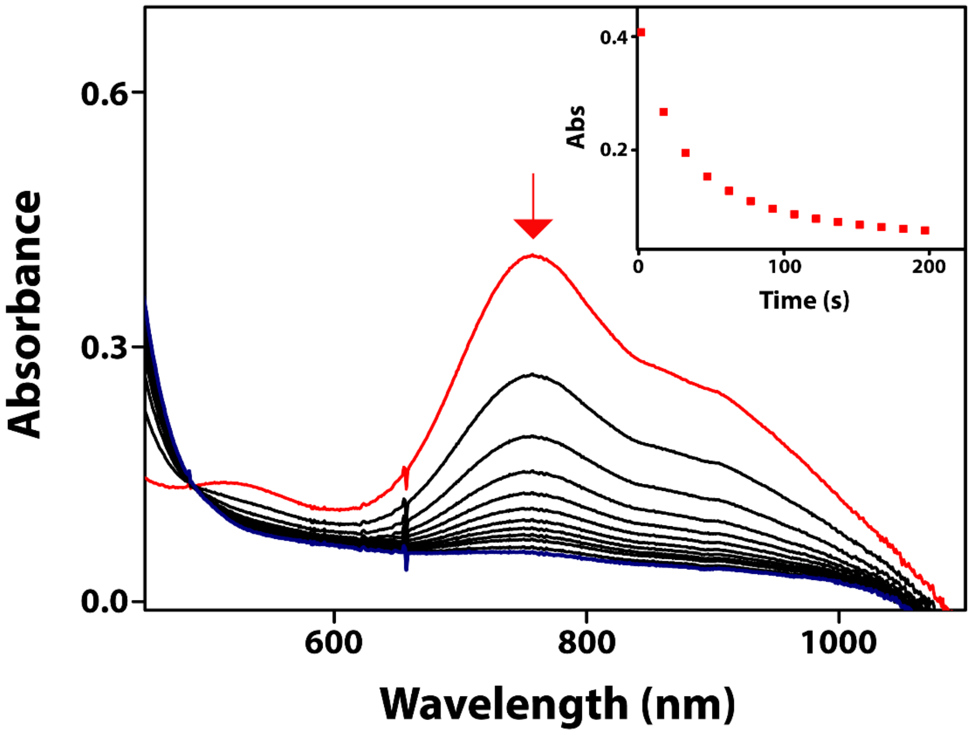

Fig. S19. UV-vis spectral changes observed after the addition of 100 equiv. of ethylbenzene to the 1 mM solution of **2b** in acetonitrile at RT.

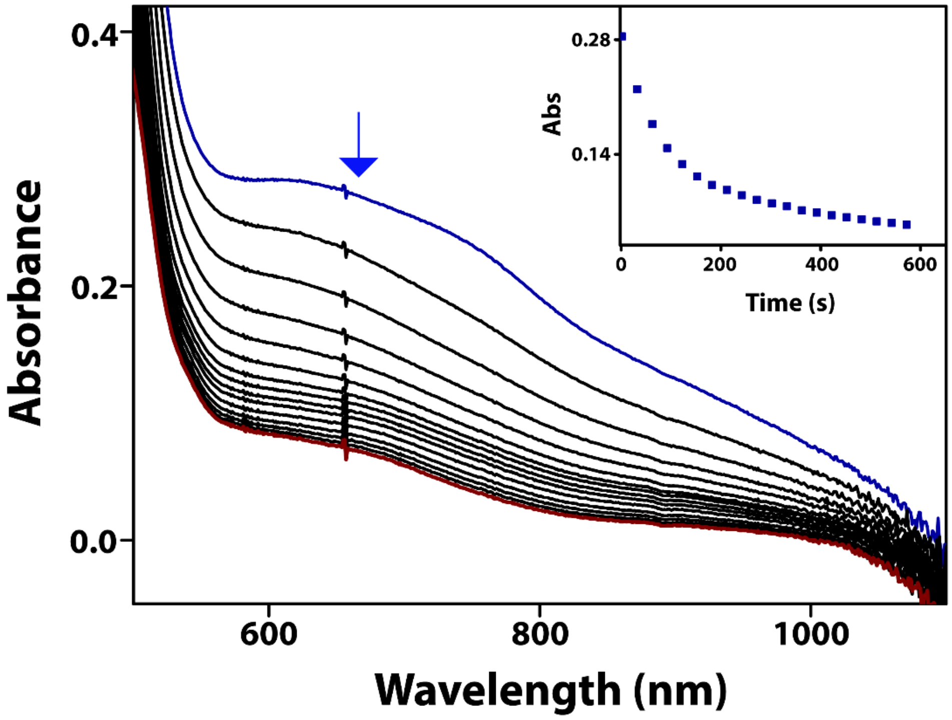

Fig. S20. UV-vis spectral changes observed after the addition of 100 equiv. of ethylbenzene to the 1 mM solution of **2c** in acetonitrile at RT.

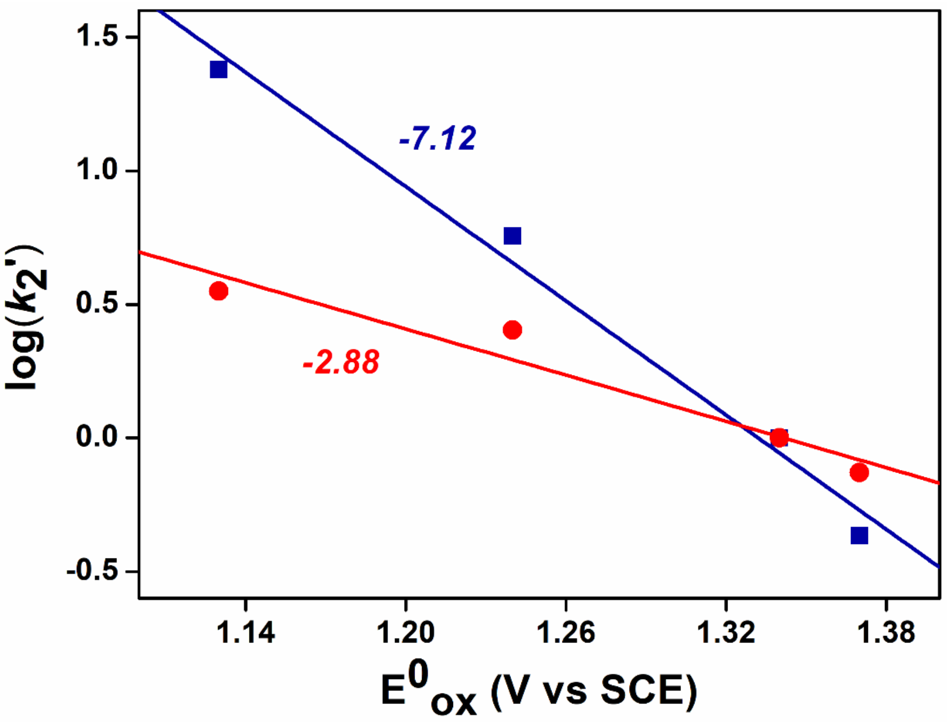

Fig. S21. Plot of log(k_X/k_H) against Hammett parameter (σ_p) of p-X-thioanisole in their reaction with **2b** (red) and **2c** (blue) at 263 K in CH_3CN, where k_X and k_H are the pseudo first-order rate constants of p-X-thioanisole and thioanisole, respectively.

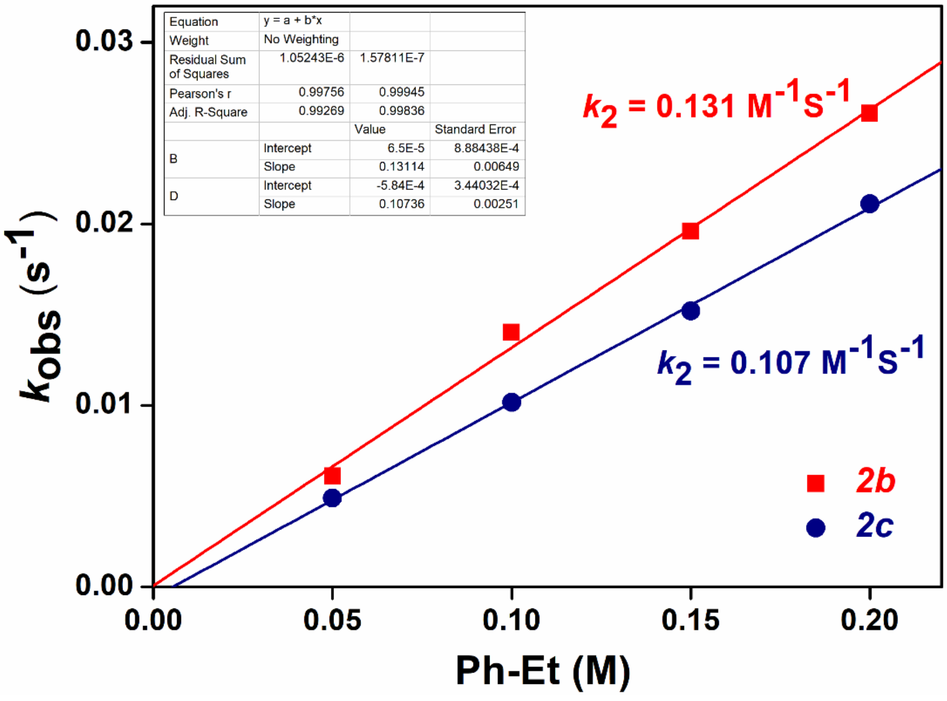

Fig. S22. The second-order rate constant determined for the reaction of **2b** (1 mM) and **2c** (1 mM) with ethylbenzene at 298 K.

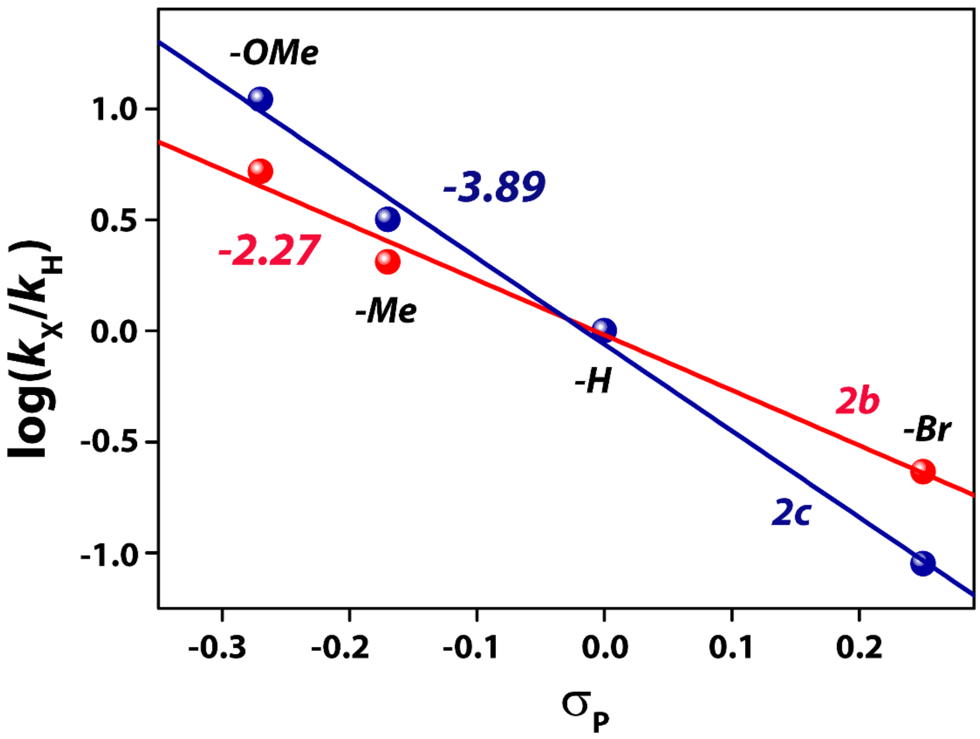

Fig. S23. Plot of log(k_X/k_H) against Hammett parameter (σ_p) of *para*-*X*-ethylbenzene in their reaction with **2b** (red) and **2c** (blue) at 298 K in CH_3CN, where k_X and k_H are the pseudo first-order rate constants of *para*-X-thioanisole and thioanisole, respectively.

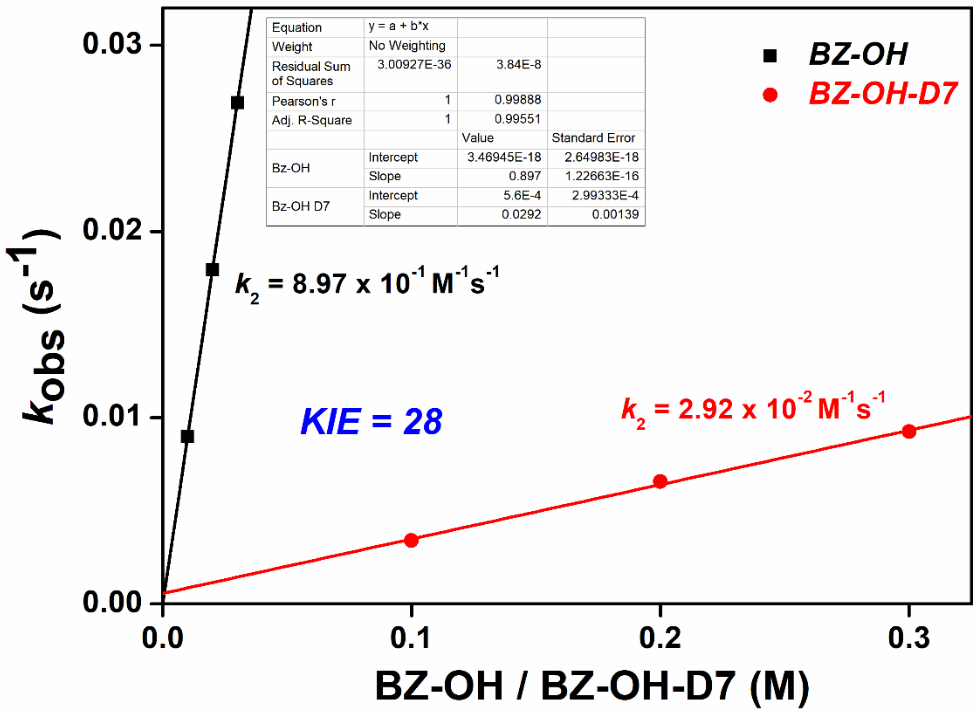

Fig. S24. The second-order rate constant determined for the reaction of **2b** (1 mM) with benzyl alcohol and benzyl alcohol-[D_7] at different concentrations to determine the KIE at 298 K.

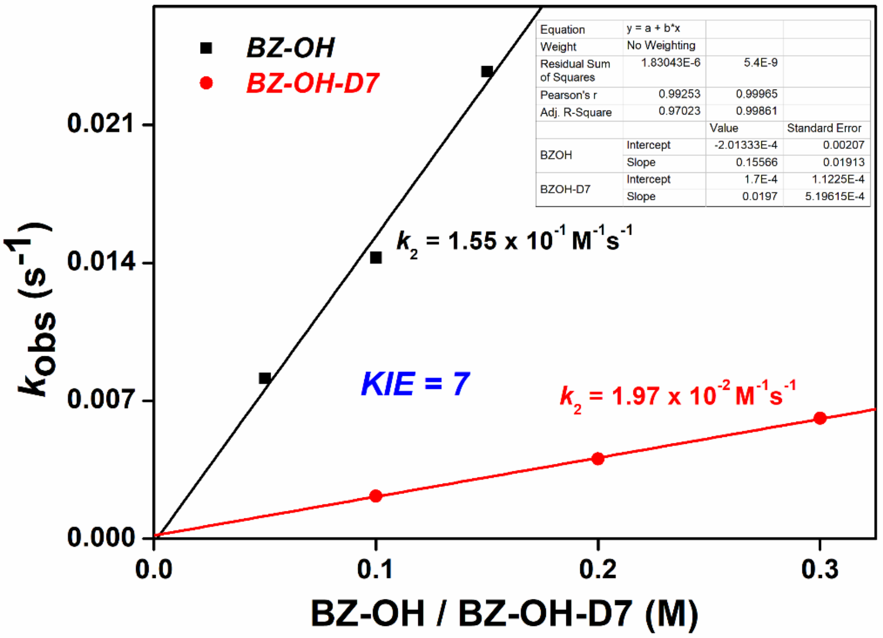

Fig. S25. The second-order rate constant determined for the reaction of **2c** (1 mM) with benzyl alcohol and benzyl alcohol-[D_7] at different concentrations to determine the KIE at 298 K.

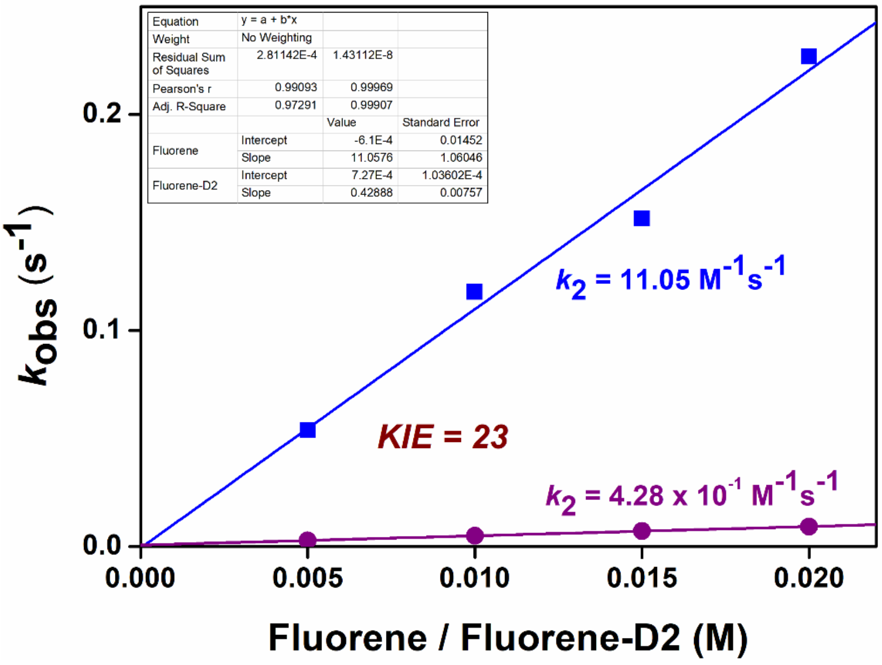

Fig. S26. The second-order rate constant determined for the reaction of **2b** (1 mM) with fluorene and fluorene-[D_2] at different concentrations to determine the KIE at 298 K.

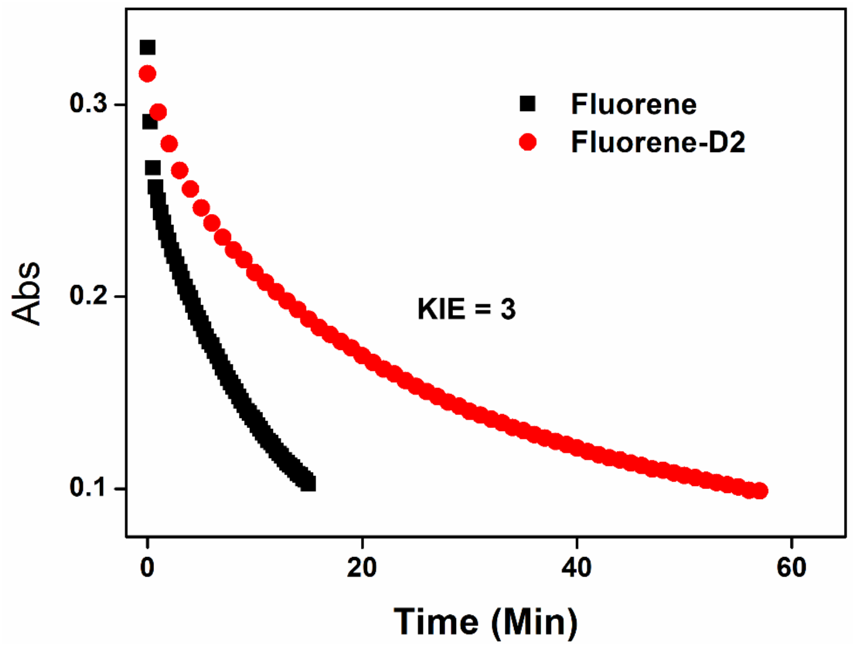

Fig. S27. Time trace for the reaction of **2c** (1 mM) with fluorene and fluorene-[D_2] (10 mM) to determine the KIE at 298 K.

Table S3. Hammett parameters and second-order rate constants (k_2) determined in the reaction of **2b** and **2c** (1 mM solution in CH_3CN) with various *para*-X-substituted thioanisole substrates in CH_3CN at 263 K.[a]

***p*-X**	σ_p [b]	σ_p^+ [b]	E^0_{ox} [c]	***Complex***	**k_2 (M^{-1} s^{-1}) x 10^{-2}**	**k_x/k_H** [d]	**log (k_x/k_H)**
-OMe	–0.27	-0.78	1.13	**2b**	3.240(2)	3.55	0.55
				**2c**	4.868(3)	23.98	1.38
-Me	–0.17	-0.31	1.24	**2b**	2.318(1)	2.54	0.40
				**2c**	1.157(3)	5.69	0.75
-H	0.00	0.00	1.34	**2b**	0.911(2)	1.0	0.00
				**2c**	0.203(4)	1.0	0.00
-Cl	0.23	0.11	1.37	**2b**	0.675(3)	0.74	–0.13
				**2c**	0.087(1)	0.42	-0.37

[a] All the reactions were followed by monitoring the UV/Vis spectral changes of the reaction solution. [b] Data taken from: H. C. Brown and Y. Okamoto, *J. Am. Chem. Soc.*, 1958, **80**, 4979. [c] Relative rate constant obtained by dividing the k_2 of *para*-X-thioanisole by k_2 of *para*-H-thioanisole.

Table S4. Hammett parameters and second-order rate constants (k_2) determined in the reaction of **2b** and **2c** (1 mM solution in CH_3CN) with various *para*-X-substituted ethylbenzene substrates in CH_3CN at 298 K.[a]

***p*-X**	σ_p [b]	***Complex***	k_2 **(M^{-1} s^{-1}) x 10^{-2}**	k_x/k_H [d]	**log (k_x/k_H)**
-OMe	–0.27	**2b**	68.5(1)	5.22	0.71
		**2c**	111.2(2)	11.00	1.04
-Me	–0.17	**2b**	26.8(1)	2.04	0.31
		**2c**	32(3)	3.16	0.50
-H	0.00	**2b**	13.1(2)	1.0	0.00
		**2c**	10.1(4)	1.0	0.00
-Br	0.25	**2b**	3.05(3)	0.232	–0.63
		**2c**	0.905(2)	0.089	-1.04

[a] All the reactions were followed by monitoring the UV/Vis spectral changes of the reaction solution. [b] Data taken from: H. C. Brown and Y. Okamoto, *J. Am. Chem. Soc.*, 1958, **80**, 4979. [c] Relative rate constant obtained by dividing the k_2 of *para*-X-ethylbenzene by k_2 of *para*-H-ethylbenzene.

Table. S5. Pseudo first-order rate constants determined for the reaction of **6b** and **6c** (1 mM) with ethylbenzene and ethylbenzene-[D_{10}] in CH_3CN at RT.

**Concentration (mM)**	**Complex**	k_{obs} **x 10^{-2} for Ethylbenzene**	k_{obs} **x 10^{-2} for Ethylbenzene-[D_{10}]**
**50**	**6b**	0.61(2)	0.042(2)
	**6c**	0.49(4)	0.0122(4)
**100**	**6b**	1.40(2)	0.0899(2)
	**6c**	1.01(3)	0.0248(3)
**150**	**6b**	1.96(1)	0.136(1)
	**6c**	1.52(2)	0.0358(2)
**200**	**6b**	2.61(2)	0.176(2)
	**6c**	2.11(4)	0.0458(4)

Table S6. KIE values determined for different substrates.

	KIE values			
	1b	***1c***	***2b***	***2c***
9,10-Dihydroanthracene	16	NR	NR	NR
Fluorene	17	NR	23	3
Ethylbenzene	53	NR	29	86
Toluene	30	NR	10	4
Benzylalcohol	7	4	28	7

Table S7. Results of Fe K-edge EXAFS simulations (best fit) for **2b**.[a]

**Bond**	**N**	**S02**	**σ^2**	**E_0**	**Δr**	**R_{eff}**	**R**
**Fe−O**	1	2.097	0.02103	4.354	0.05133	1.63010	1.68143
**Fe−N**	1	2.097	0.02103	4.354	-0.01429	2.06470	2.05041
**Fe−S**	1	2.097	0.02103	4.354	0.10067	2.34750	2.44817
**Fe−C**	1	2.097	0.02103	4.354	0.04342	2.91620	2.95962

[a] Fitting range was k = 3 − 8 $Å^{-1}$. R is in units of Å; σ^2 is in units of 10^{-3} Å; E_0 is in units of eV; R-factor for the fitting is 0.0051370, R-factor represents the fractional misfit of the data, while χ^2 for the fitting is 131.4498827, χ^2 represents the fitting metric normalized by the number of independent data points in a given fit.

Part III: Computational Results

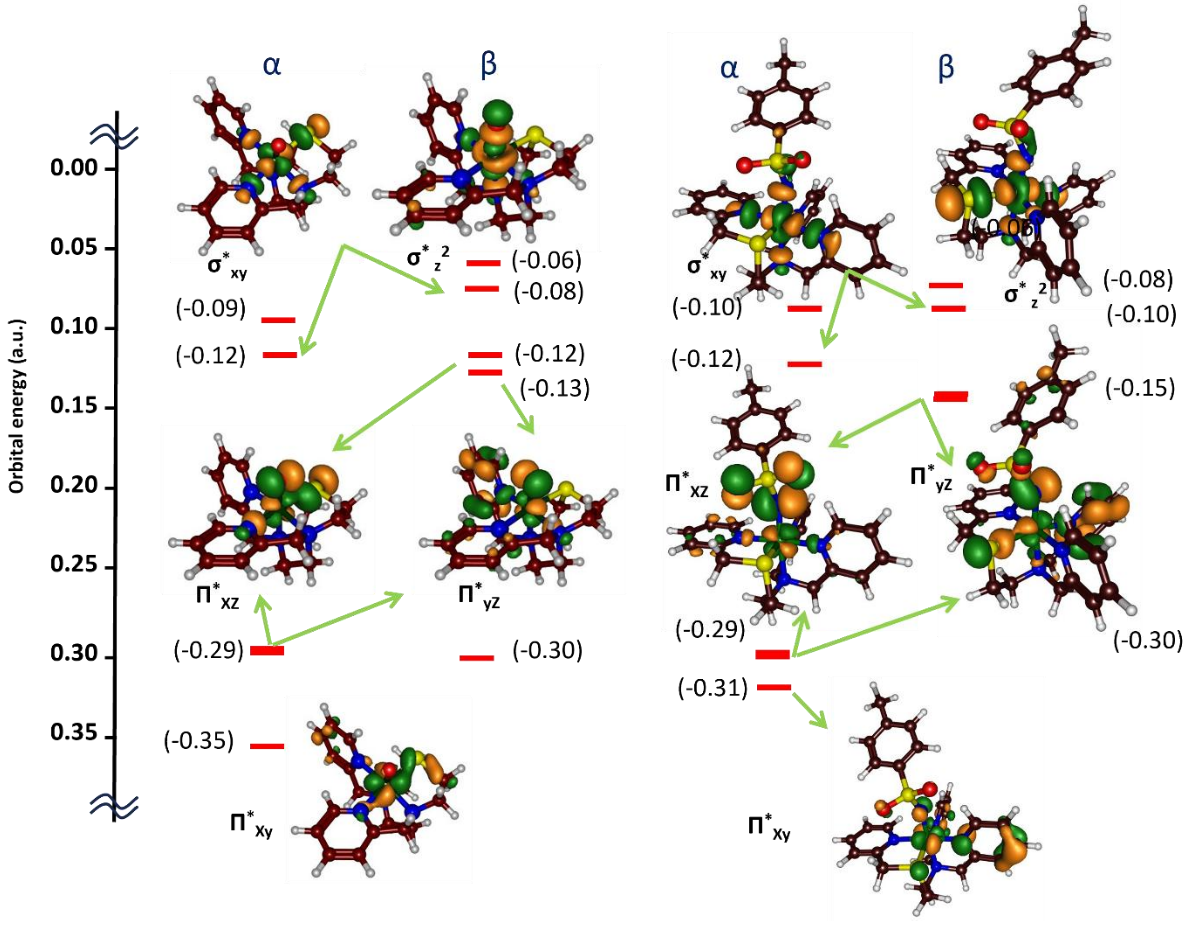

Fig. S28. Orbital diagram of $[Fe^{IV}(O)(STPEN)]^{2+}$ **(2b)** on the left-hand-side and $[Fe^{IV}(NTs)(STPEN)]^{2+}$ **(2c)** on the right-hand-side. The energy levels of the α- and β-set of molecular valence orbitals are given (with energies in au) of both complexes. Also shown are orbital drawings of the relevant molecular orbitals.

Table S8 Absolute energies of UB3LYP/BS1 optimized geometries of isolated species **2b**, and substrates.

	E (au)	ZPE (au)	G (au)	E (au)
	**BS1**	**BS1**	**BS1**	**BS2**
**^{3}2b**	-1590.968789	0.400181	-1590.618691	-1591.296569
**^{5}2b**	-1590.955568	0.39802	-1590.610324	-1591.286565
**DMS**	-478.0130544	0.076155	-477.963954	-478.482888
**CHD**	-233.4156192	0.122355	-233.321706	-233.069209

Table S9. Relative energies of isolated species **2b** as optimized with UB3LYP/BS1.

	ΔE (kcal mol^{-1})	ΔE+ZPE (kcal mol^{-1})	ΔG (kcal mol^{-1})	ΔE (kcal mol^{-1})	ΔG (kcal mol^{-1})
	**BS1**	**BS1**	**BS1**	**BS2**	**BS2**
**^{3}2b**	0.00	0.00	0.00	0.00	0.00
**^{5}2b**	8.30	6.94	5.25	6.28	3.23

Table S10. Absolute energies of UB3LYP/BS1 optimized local minima and transition states along the reaction mechanism of $[Fe^{IV}(O)(STPeN)]^{2+}$ **(2b)** with **DMS**.

	E (au)	ZPE (au)	G (au)	E (au)
	**BS1**	**BS1**	**BS1**	**BS2**
**^{3}RC$_{2b}$**	-2068.983973	0.478588	-2068.568336	-2069.367774
**^{5}RC$_{2b}$**	-2068.971340	0.476466	-2068.560986	-2069.358365
**^{3}TS$_{SO,2b}$**	-2068.961572	0.476673	-2068.54496	-2069.344303
**^{5}TS$_{SO,2b}$**	-2068.968466	0.475498	-2068.557427	-2069.354778
**^{3}PC$_{2b}$**	-2069.014936	0.478007	-2068.597695	-2069.393609
**^{5}PC$_{2b}$**	-2069.033026	0.477048	-2068.618053	-2069.412662

Table S11. Relative (free) energies (in kcal mol^{-1}) of UB3LYP/BS1 optimized local minima and transition states along the reaction mechanism of $[Fe^{IV}(O)(STPeN)]^{2+}$ **(2b)** with DMS with respect to isolated reactants (**^{3}2b + DMS**).

	ΔE	ΔE+ZPE	ΔG	ΔE	ΔG
	**BS1**	**BS1**	**BS1**	**BS2**	**BS2**
**^{3}RC$_{2b}$**	0.00	0.00	0.00	0.00	0.00
**^{5}RC$_{2b}$**	7.93	6.60	4.61	5.90	2.59
**^{3}TS$_{SO,2b}$**	14.06	12.86	14.67	14.73	15.34
**^{5}TS$_{SO,2b}$**	9.73	7.79	6.85	8.16	5.27
**^{3}PC$_{2b}$**	-19.43	-19.79	-18.42	-16.21	-15.21
**^{5}PC$_{2b}$**	-30.78	-31.75	-31.20	-28.17	-28.58

Table S12. Absolute energies (in au) of UB3LYP/BS1 optimized local minima and transition states along the reaction mechanism of $[Fe^{IV}(O)(STPeN)]^{2+}$ **(2b)** with CHD.

	E (au)	ZPE (au)	G (au)	E (au)
	**BS1**	**BS1**	**BS1**	**BS2**
^{3}**RC**$_{H,2b}$	-1824.387169	0.524967	-1823.927658	-1824.781728
^{5}**RC**$_{H,2b}$	-1824.375093	0.522487	-1823.920821	-1824.771369
^{3}**TS1**$_{HA,2b}$	-1824.368540	0.518470	-1823.910996	-1824.763358
^{5}**TS1**$_{HA,2b}$	-1824.367556	0.517851	-1823.915886	-1824.766278
^{3}**INT**$_{H,2b}$	-1824.409222	0.521760	-1823.951733	-1824.808924
^{5}**INT**$_{H,2b}$	-1824.422392	0.517771	-1823.974879	-1824.818577
^{3}**TS2**$_{HA,2b}$	-1824.402201	0.519168	-1823.947045	-1824.802633
^{3}**PC**$_{2b}$	-1824.478781	0.522587	-1824.023704	-1824.880131
^{5}**PC**$_{2b}$	-1824.498097	0.521458	-1824.045511	-1824.901379

Table S13. Absolute energies of UB3LYP/BS1 optimized local minima and transition states along the reaction mechanism of $[Fe^{IV}(O)(STPeN)]^{2+}$ **(2b)** with CHD.

	ΔE	ΔE+ZPE	ΔG	ΔE	ΔG
	**BS1**	**BS1**	**BS1**	**BS2**	**BS2**
^{3}**RC**$_{H,2b}$	0.00	0.00	0.00	0.00	0.00
^{5}**RC**$_{H,2b}$	7.58	6.02	4.29	6.50	3.21
^{3}**TS1**$_{HA,2b}$	11.69	7.61	10.46	11.53	10.29
^{5}**TS1**$_{HA,2b}$	12.31	7.84	7.39	9.70	4.77
^{3}**INT**$_{H,2b}$	-13.84	-15.85	-15.11	-17.07	-18.33
^{5}**INT**$_{H,2b}$	-22.10	-26.62	-29.63	-23.12	-30.65
^{3}**TS2**$_{HA,2b}$	-9.43	-13.07	-12.17	-13.12	-15.85
^{3}**PC**$_{2b}$	-57.49	-58.98	-60.27	-61.75	-64.53
^{5}**PC**$_{2b}$	-69.61	-71.81	-73.95	-75.08	-79.43

Table S14. Absolute energies of UB3LYP/BS1 optimized isolated species **2c**.

	E (au)	ZPE (au)	G (au)	E (au)
	**BS1**	**BS1**	**BS1**	**BS2**
^{3}**2c**	-2390.017079	0.529345	-2389.55213	-2390.48108
^{5}**2c**	-2390.007761	0.529026	-2389.546949	-2390.47422

Table S15. Relative energies (in kcal mol^{-1}) of UB3LYP/BS1 optimized isolated species **2c**.

	ΔE	ΔE+ZPE	ΔG	ΔE	ΔG
	**BS1**	**BS1**	**BS1**	**BS2**	**BS2**
**^{3}2c**	0.00	0.00	0.00	0.00	0.00
**^{5}2c**	5.85	5.65	3.25	4.30	1.71

Table S16. Absolute energies of UB3LYP/BS1 optimized local minima and transition states along the reaction mechanism of $[Fe^{IV}(NTS)(STPeN)]^{2+}$ **(2c)** with **DMS**.

	E (au)	ZPE (au)	G (au)	E (au)
	**BS1**	**BS1**	**BS1**	**BS2**
**^{3}RC$_{2c}$**	-2868.028789	0.608038	-2867.503847	-2868.54846
**^{5}RC$_{2c}$**	-2868.021701	0.605871	-2867.495749	-2868.54375
**^{3}TS$_{SO,2c}$**	-2868.005613	0.607466	-2867.470578	-2868.52268
**^{5}TS$_{SO,2c}$**	-2868.009905	0.606111	-2867.477226	-2868.53006
**^{3}PC$_{2c}$**	-2868.048559	0.608692	-2867.514367	-2868.56189
**^{5}PC$_{2c}$**	-2868.062165	0.608405	-2867.526667	-2868.57649

Table S17. Relative (free) energies (in kcal mol^{-1}) of UB3LYP/BS1 optimized local minima and transition states along the reaction mechanism of $[Fe^{IV}(NTS)(STPeN)]^{2+}$ **(2c)** with DMS with respect to isolated reactants (**^{3}2c + DMS**).

	ΔE	ΔE+ZPE	ΔG	ΔE	ΔG
	**BS1**	**BS1**	**BS1**	**BS2**	**BS2**
**^{3}RC$_{2c}$**	0.00	0.00	0.00	0.00	0.00
**^{5}RC$_{2c}$**	4.45	3.09	5.08	2.96	3.59
**^{3}TS$_{SO,2c}$**	14.54	14.18	20.88	16.18	22.51
**^{5}TS$_{SO,2c}$**	11.85	10.64	16.70	11.55	16.40
**^{3}PC$_{2c}$**	-12.41	-12.00	-6.60	-8.43	-2.62
**^{5}PC$_{2c}$**	-20.94	-20.71	-14.32	-17.59	-10.97

Table S18. Absolute energies (in au) of UB3LYP/BS1 optimized local minima and transition states along the reaction mechanism of $[Fe^{IV}(O)(STPeN)]^{2+}$ **(2c)** with **CHD**.

	E (au)	ZPE (au)	G (au)	E (au)
	**BS1**	**BS1**	**BS1**	**BS2**
^{3}**RC$_{H,2c}$**	-2623.435993	0.653988	-2622.863346	-2623.96656
^{5}**RC$_{H,2c}$**	-2623.425447	0.651983	-2622.853439	-2623.95740
^{3}**TS1$_{H1,2c}$**	-2623.413453	0.649939	-2622.835812	-2623.94249
^{5}**TS1$_{H1,2c}$**	-2623.421933	0.646887	-2622.851032	-2623.95684
^{3}**INT$_{2c}$**	-2623.465711	0.653102	-2622.888184	-2623.99851
^{5}**INT$_{2c}$**	-2623.500815	0.650373	-2622.934040	-2624.02991
^{3}**TS2$_{H2,2c}$**	-2623.474368	0.651182	-2622.899920	-2623.99993
^{3}**PC$_{2c}$**	-2623.539870	0.656016	-2622.960272	-2624.06721
^{5}**PC$_{2c}$**	-2623.563202	0.654283	-2622.991244	-2624.09403

Table S19. Absolute energies (in kcal mol^{-1}) of UB3LYP/BS1 optimized local minima and transition states along the reaction mechanism of $[Fe^{IV}(O)(STPeN)]^{2+}$ **(2b)** with CHD.

	ΔE	ΔE+ZPE	ΔG	ΔE	ΔG
	**BS1**	**BS1**	**BS1**	**BS2**	**BS2**
^{3}**RC$_{H,2c}$**	0.00	0.00	0.00	0.00	0.00
^{5}**RC$_{H,2c}$**	6.62	5.36	6.22	5.75	5.35
^{3}**TS1$_{H1,2c}$**	14.14	11.60	17.28	15.10	18.24
^{5}**TS1$_{H1,2c}$**	8.82	4.37	7.73	6.10	5.00
^{3}**INT$_{2c}$**	-18.65	-19.20	-15.59	-20.05	-16.99
^{5}**INT$_{2c}$**	-40.68	-42.94	-44.36	-39.75	-43.44
^{3}**TS2$_{H1,2c}$**	-24.08	-25.84	-22.95	-20.94	-19.81
^{3}**PC$_{2c}$**	-65.18	-63.91	-60.82	-63.16	-58.80
^{5}**PC$_{2c}$**	-79.82	-79.64	-80.26	-79.99	-80.42

Table S20. Group spin densities and charges of UB3LYP?BS1 optimized local minima and transition states along the reaction mechanism of $[Fe^{IV}(O)(STPeN)]^{2+}$ **(2b)** with **DMS**. B3LYP optimized structures in the solvent with the dielectric constantof 35.688.

	ρ_{Fe}	ρ_{O}	ρ_{LIG}	ρ_{Sub}	Q_{Fe}	Q_{O}	Q_{LIG}	Q_{Sub}
**$^{3}RC_{2b}$**	1.16	0.91	-0.07	0.00	0.39	-0.40	2.01	0.01
**$^{5}RC_{2b}$**	2.94	0.75	0.31	0.00	0.60	-0.39	1.78	0.01
**$^{3}TS_{SO,2b}$**	1.33	0.43	-0.04	0.27	0.42	-0.57	1.59	0.56
**$^{5}TS_{SO,2b}$**	3.26	0.55	0.33	-0.15	0.61	-0.42	1.63	0.17
**$^{3}PC_{2b}$**	1.95	0.03	0.02	0.00	0.36	-0.67	1.32	0.98
**$^{5}PC_{2b}$**	3.72	0.05	0.22	0.01	0.53	-0.68	1.18	0.97

Table S21. Group spin densities and charges of UB3LYP/BS1 optimized local minima and transition states along the reaction mechanism of $[Fe^{IV}(NTS)(STPeN)]^{2+}$ **(2c)** with **DMS**. B3LYP optimized structures in the solvent with a dielectric constantof 35.688.

	ρ_{Fe}	ρ_{NTS}	ρ_{LIG}	ρ_{Sub}	Q_{Fe}	Q_{O}	Q_{LIG}	Q_{Sub}
**$^{3}RC_{2c}$**	0.93	1.12	-0.06	0.01	0.25	-0.27	2.02	0.00
**$^{5}RC_{2c}$**	2.83	0.93	0.23	0.02	0.44	-0.23	1.76	0.03
**$^{3}TS_{SO,2c}$**	2.43	-0.10	0.03	-0.36	0.41	-0.43	1.49	0.53
**$^{5}TS_{SO,2c}$**	2.94	0.51	0.18	0.37	0.44	-0.42	1.59	0.39
**$^{3}PC_{2c}$**	2.02	0.02	-0.04	0.00	0.30	-0.62	1.46	0.85
**$^{5}PC_{2c}$**	3.75	0.02	0.22	0.01	0.46	-0.63	1.23	0.94

Table S22. Group spin densities and charges of UB3LYP/BS1 optimized local minima and transition states along the reaction mechanism of $[Fe^{IV}(O)(STPeN)]^{2+}$ **(2b)** with **CHD**. B3LYP optimized structures in the solvent with a dielectric constantof 35.688.

	ρ_{Fe}	ρ_{O}	ρ_{LIG}	ρ_{Sub}	Q_{Fe}	Q_{O}	Q_{LIG}	Q_{Sub}
**$^{3}RC_{2b}$**	1.15	0.92	-0.06	0.00	0.39	-0.40	1.56	0.00
**$^{5}RC_{2b}$**	2.94	0.75	0.18	0.00	0.61	-0.38	1.43	0.00
**$^{3}TS_{H1,2b}$**	0.89	0.75	-0.05	0.41	0.37	-0.54	1.40	0.32
**$^{5}TS_{H1,2b}$**	3.58	0.43	0.26	-0.38	0.65	-0.53	1.21	0.36
**$^{3}Int_{2b}$**	0.90	0.18	-0.06	0.98	0.31	-0.70	1.45	0.49
**$^{5}Int_{2b}$**	3.95	0.43	0.37	-0.88	0.70	-0.78	1.15	0.61
**$^{3}TS_{H2,2b}$**	1.40	0.05	-0.07	0.63	0.36	-0.73	1.25	0.75
**$^{3}PC_{2b}$**	1.97	0.02	-0.02	0.00	0.36	-0.77	1.09	1.02
**$^{5}PC_{2b}$**	3.73	0.03	0.19	0.00	0.56	-0.80	0.96	1.04

Table S23. Group spin densities and charges of UB3LYP/BS1 optimized local minima and transition states along the reaction mechanism of $[Fe^{IV}(O)(STPeN)]^{2+}$ **(2c)** with **CHD**. B3LYP optimized structures in the solvent with a dielectric constant of 35.688.

	ρ_{Fe}	ρ_{O}	ρ_{LIG}	ρ_{Sub}	Q_{Fe}	Q_{O}	Q_{LIG}	Q_{Sub}
**$^3RC_{2c}$**	0.97	1.12	-0.06	0.00	0.32	-0.21	1.48	-0.02
**$^5RC_{2c}$**	2.85	0.91	0.11	0.00	0.45	-0.22	1.42	-0.02
**$^3TS_{H1,2c}$**	0.86	0.66	-0.05	0.56	0.26	-0.52	1.40	0.41
**$^5TS_{H1,2c}$**	3.88	0.19	0.34	-0.54	0.54	-0.56	1.16	0.52
**$^3Int_{2c}$**	0.97	0.14	-0.07	0.99	0.23	-0.60	1.53	0.39
**$^5Int_{2c}$**	3.72	0.09	0.14	0.00	0.47	-0.86	0.83	1.31
**$^3TS_{H2,2c}$**	1.99	0.00	-0.03	0.01	0.27	-0.74	1.03	1.18
**$^3PC_{2c}$**	1.96	-0.02	0.02	0.00	0.49	-0.61	0.98	0.88
**$^5PC_{2c}$**	3.74	0.02	0.19	0.00	0.33	-0.55	1.08	0.88

Table S24. Test calculations on the spin state ordering of ^{3,5}**1b**, ^{3,5}**1c**, ^{3,5}**2b** and ^{3,5}**2c** complexes as calculated through full geometry optimizations with an alternative density functional approach and basis set BS2 in Gaussian-09. A CPCM solvent model was included with a dielectric constant mimicking acetonitrile. Free energies reported at 233 K.[a]

	B3LYP [b]	B3LYP-GD3BJ	OLYP	PBE0
**1b**	ND	-0.11	-1.23	-3.00
**1c**	ND	-3.20	-3.54	-9.56
**2b**	3.20	2.90	2.37	-0.14
**2c**	1.71	5.85	1.47	-4.63

a. Values for the free energy differences between the triplet and quintet optimized geometries are in kcal mol^{-1}. A positive value denotes a triplet spin ground state. Free energies contain zero-point, thermal, entropic and solvent corrections.
b. UB3LYP/BS2//UB3LYP/BS1 result.

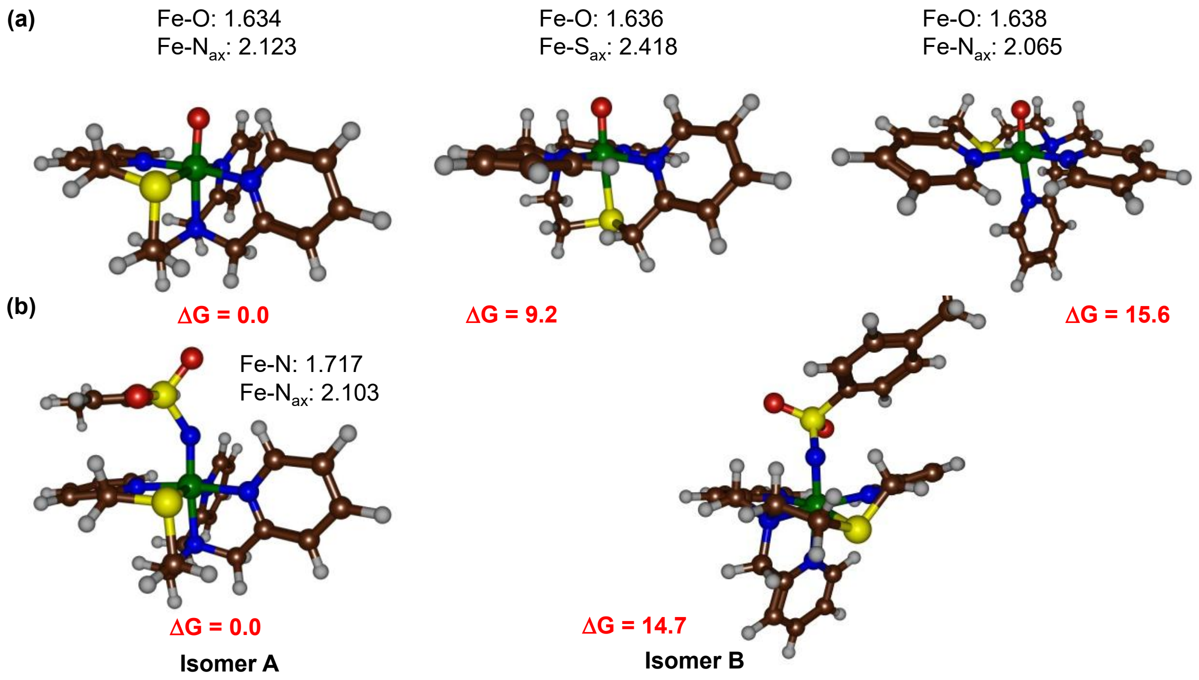

Fig. S29. UB3LYP/BS2 optimized geometries of isomers of (a) ^{3}**2b** and (b) ^{3}**3b**. Bond lengths are in Å and relative free energies in kcal mol^{-1} and include zero-point, thermal, solvent and entropic corrections. As can be seen isomer A is by far the most stable for the -oxo and tosylimido structures and consequently was used for the reactivity studies.

Part IV: Cartesian coordinates

^{3}2b

Fe	-0.005502274293	-0.004113125109	0.012259534339
S	-0.016837848998	-0.056995148790	2.359089665665
N	1.987935979349	0.003355715944	0.010274634786
N	-2.061684750711	-0.108700101610	0.167136963169
N	0.285606374755	-2.132106005454	0.126831574493
N	0.040824155185	-0.427761831659	-1.938036318750
C	2.618638202439	-1.186664492043	0.073234175990
C	2.706928495571	1.148871248793	-0.048787104411
H	2.136888336274	2.070466834310	-0.095657486664
C	-0.074376183587	-1.740359581702	-2.262993969008
C	-2.645225204332	0.093635538752	1.374532131634
C	-0.356739838538	-2.677989749582	1.368658680749
H	-0.095837975492	-3.738685550242	1.496026106590
H	-1.439644459622	-2.615417647915	1.237919779015
C	-2.857278165050	-0.213263400855	-0.923861275142
H	-2.363355072075	-0.336849673488	-1.878221866505
C	-0.366681747157	-2.671833569403	-1.108859270394
H	-1.449173227953	-2.716203729633	-0.936954675322
H	-0.031633680506	-3.695441398707	-1.322203804901
C	0.078844937387	-1.894163370038	2.603728773607
H	1.127174342441	-2.071556614660	2.869416981710
H	-0.529233295089	-2.158066352447	3.476940733615
C	-1.785885350745	0.350079514661	2.588485484374
H	-2.159137081516	-0.175245500450	3.475201449521
H	-1.792248962341	1.424020216281	2.823916421862
C	4.095005276261	1.138590002666	-0.053871927251
H	4.637782461505	2.078659325987	-0.110132503854
C	4.014398830121	-1.264024003998	0.081639499506
H	4.497983341282	-2.237193338936	0.133230021699

C	1.764400352204	-2.432627412593	0.094997682698
H	1.992714451441	-3.032513236567	-0.795514000255
H	2.034844549794	-3.062974359150	0.952086518897
C	-4.242521355305	-0.150656871926	-0.857087465732
H	-4.825974079233	-0.238148708035	-1.769757727821
C	0.252153655817	0.493652831243	-2.900428064289
H	0.334522064531	1.525389882093	-2.573656342461
C	-4.035488120535	0.150938026109	1.512987354696
H	-4.463147886806	0.307267003823	2.500313819394
C	4.761556094069	-0.089631834742	0.013925809798
H	5.848916477847	-0.129313330415	0.012514038583
C	0.028686940300	-2.164410933117	-3.586808187471
H	-0.056211971117	-3.223178566363	-3.821023978508
C	0.241993818128	-1.214799854444	-4.590063166535
H	0.326410410037	-1.525207518445	-5.629585621304
C	-4.847759881373	0.027864375103	0.388815640028
H	-5.930327462209	0.079785707912	0.481758086720
C	0.352819566392	0.133912472084	-4.241220151753
H	0.519866260193	0.903144272528	-4.990595875687
O	-0.102245885015	1.617241948424	-0.126134761567

^{5}2b

Fe	-0.010714114188	-0.034179850116	0.033525555402
S	-0.047501091587	-0.079283628664	2.631088443354
N	2.039120770282	0.003806921688	0.165894015291
N	-2.152550288641	-0.154453412883	0.381742731600
N	0.310434581658	-2.156318383863	0.245227176861
N	-0.063173622054	-0.663244713010	-1.998352795863
C	2.655743866270	-1.193933396395	0.260743003325
C	2.759989733519	1.149123281724	0.175250483026
H	2.194873426110	2.072882662628	0.097722569883
C	-0.161883571232	-2.002547917219	-2.176984851112

C	-2.709101466111	-0.015899765769	1.604057255410
C	-0.269258677074	-2.683945413605	1.530804011771
H	0.004995772864	-3.742403183292	1.645370307642
H	-1.356984891613	-2.634401902315	1.443064860135
C	-2.962488048576	-0.207475536467	-0.703629162887
H	-2.474226860430	-0.282764077658	-1.667041048641
C	-0.385312854762	-2.809008677138	-0.917710086878
H	-1.458541607606	-2.842997944337	-0.695133116615
H	-0.046926069403	-3.846648439775	-1.037209402808
C	0.194888137700	-1.908678950680	2.759695690224
H	1.267838459487	-2.024852091659	2.950981382397
H	-0.333410551590	-2.267875411032	3.650399421849
C	-1.848494778224	0.167089477572	2.840618293947
H	-2.199353393361	-0.473399411887	3.658580801665
H	-1.942451826514	1.205926292634	3.187171258427
C	4.144441947839	1.133996597760	0.273652165467
H	4.695914202296	2.070441122224	0.267815066998
C	4.045307306724	-1.276551450231	0.380075596727
H	4.524694850332	-2.249351409579	0.460069263152
C	1.797489675264	-2.433943629816	0.155387190372
H	2.004231196393	-2.906269046397	-0.813591759433
H	2.084244195776	-3.171097773339	0.916614347978
C	-4.346179481469	-0.157006163573	-0.614986535079
H	-4.946235824463	-0.204930285985	-1.519722416003
C	0.078791444010	0.153181798973	-3.064441286968
H	0.151381876508	1.216831455204	-2.856214295811
C	-4.100248899118	0.028527418267	1.765433556895
H	-4.514714068541	0.133334247279	2.765059961637
C	4.796428910344	-0.100297621634	0.381763406813
H	5.880460990670	-0.144175518213	0.465542112606
C	-0.114424349841	-2.569084398900	-3.449323036484

H	-0.185330369124	-3.647752473363	-3.567097679928
C	0.023964182845	-1.727230283858	-4.557351053062
H	0.060413199317	-2.146774257432	-5.560475326809
C	-4.929901062501	-0.042719391947	0.650381700839
H	-6.011258959801	-0.001495510539	0.764141100340
C	0.121056306432	-0.345776621152	-4.363104941417
H	0.231399375399	0.337796678308	-5.200538387083
O	-0.117054429150	1.569755455337	-0.208170597383

^{3}2c

Fe	0.019875809202	-0.011246630005	0.039456133978
S	-0.020429348623	-0.091404210394	2.383051217877
N	2.032735420358	-0.012347123723	0.068897915565
N	-2.040757330447	-0.104120773219	0.173819712347
N	0.284636526341	-2.120165105212	0.124955856519
N	0.057907474574	-0.474991627879	-1.926492091970
C	2.634562142647	-1.220523005346	0.044109747817
C	2.797635246984	1.104309877902	0.115593874413
H	2.270563948228	2.048199862564	0.150622976943
C	-0.196330032059	-1.772475892716	-2.230310769371
C	-2.651259712017	-0.114541777783	1.383816289760
C	-0.213673712204	-2.694110473184	1.417345439261
H	0.083420302262	-3.748911082501	1.500409379917
H	-1.306607937306	-2.660796124456	1.404731759601
C	-2.817146518149	-0.047485580616	-0.937359021445
H	-2.300007740993	0.012040846043	-1.885496286657
C	-0.484000793412	-2.658815203522	-1.042535947250
H	-1.551252622766	-2.637281781193	-0.796529541163
H	-0.214420553610	-3.704168969654	-1.242545943400
C	0.337721057625	-1.897228034598	2.589909991041
H	1.426767642501	-1.966670372800	2.684987521678
H	-0.096113454704	-2.221098560663	3.542519772598

C	-1.833690695600	-0.040938208097	2.651370598787
H	-2.101836397950	-0.837249580911	3.356763883235
H	-2.026729659259	0.914165412484	3.153300191508
C	4.185033535111	1.049540982661	0.127757812616
H	4.756738092098	1.973332935005	0.160635317827
C	4.025746386037	-1.347428753391	0.067114735227
H	4.474964596179	-2.337664431498	0.049360244567
C	1.750948266859	-2.435421339581	-0.065120029987
H	1.893809811927	-2.881315430268	-1.056612540350
H	2.060454813604	-3.203395398908	0.653844255594
C	-4.203358610769	-0.045902646789	-0.891760904688
H	-4.769708038866	0.000427737541	-1.817847578254
C	0.343035305006	0.401576123256	-2.911352694067
H	0.548642845781	1.419195674401	-2.599842731077
C	-4.045732146238	-0.127404830393	1.499119706919
H	-4.494384727196	-0.143672007995	2.489192079975
C	4.813354877648	-0.198791312000	0.103552198495
H	5.898296574073	-0.274789393650	0.114955068200
C	-0.188564017788	-2.221822126558	-3.548764310345
H	-0.387688397470	-3.269141532099	-3.761968328262
C	0.087706723067	-1.312897099250	-4.573495451610
H	0.099056139964	-1.641812358257	-5.610044912708
C	-4.835002290291	-0.098970787601	0.354094934388
H	-5.919853041670	-0.103193645110	0.431625227465
C	0.363683732580	0.017810876331	-4.248504496210
H	0.595980167032	0.752115531822	-5.015102281589
N	-0.124641554770	1.733364455255	-0.165622308103
O	-1.457786672014	2.957062758236	1.654053994301
S	-0.427757203016	3.165533523497	0.617980275651
O	0.886710419657	3.691985460691	1.044548490331
C	-1.088320765096	4.234484946453	-0.651726104648

C	-0.211147644573	4.880464943932	-1.533474209200
C	-2.470890548382	4.432301322666	-0.734671874965
H	-3.141513781643	3.944773631678	-0.034726304871
H	0.863983676719	4.740029372679	-1.456328955054
C	-0.736808377490	5.730867737708	-2.504674594758
H	-0.057547549729	6.237635376528	-3.187686760729
C	-2.121800037323	5.953959619030	-2.609141657655
C	-2.974644783259	5.289635723227	-1.712769929844
C	-2.674923941628	6.913247129576	-3.637184712209
H	-4.049542582559	5.450594559468	-1.773916565651
H	-3.701012622353	6.651062533502	-3.920663715741
H	-2.697079406228	7.937183156610	-3.235741612828
H	-2.055665989756	6.930772469815	-4.541270260576

[5]2c

Fe	0.133132525566	0.099759051445	-0.145756745075
S	0.055174051840	-0.003282775638	2.445277286503
N	2.186453366142	-0.023005118549	0.108984853437
N	-1.986564563415	0.097114244341	0.160208421743
N	0.280847463079	-2.021907687713	-0.005049077919
N	0.091464780182	-0.509195130289	-2.204798804859
C	2.679387464681	-1.280689293014	0.162677932001
C	3.010192578761	1.026676368681	0.316584610547
H	2.558792130545	2.010077571852	0.278020087098
C	-0.192098297721	-1.814942556279	-2.403032715920
C	-2.576260409091	0.082343154207	1.371726772642
C	-0.257552662504	-2.543877389082	1.291629945675
H	-0.046944408746	-3.618123987170	1.366415642700
H	-1.342446749953	-2.425236606690	1.266834238091
C	-2.760593200488	0.169757006229	-0.948046538866
H	-2.241057360786	0.217481458581	-1.895763162531
C	-0.507677641090	-2.587470664339	-1.146522712872

H	-1.569438371838	-2.487983498314	-0.904620995187
H	-0.295763970577	-3.654998999680	-1.267599953588
C	0.326505265863	-1.823695827535	2.496762829895
H	1.409452931051	-1.948003890017	2.576515639815
H	-0.115589463876	-2.214216418112	3.417069954305
C	-1.754303128092	0.114515180276	2.642282423153
H	-2.080460867831	-0.667284843748	3.334152604716
H	-1.917363635467	1.077222344548	3.133339392794
C	4.361318810079	0.853573625021	0.584226692335
H	4.990384731666	1.721679122030	0.744009453345
C	4.022202560381	-1.523899956590	0.443212282399
H	4.388746373665	-2.543533808096	0.488423834862
C	1.730564677056	-2.394187105527	-0.185061406424
H	1.885363651160	-2.645415577405	-1.238832417894
H	1.952478890098	-3.300648027413	0.386147403916
C	-4.144858431989	0.185856362525	-0.894360299106
H	-4.716358321189	0.238590101076	-1.813739852251
C	0.370944466397	0.286207537843	-3.253404568208
H	0.591113887313	1.322301523162	-3.020635174139
C	-3.969902134562	0.087482935488	1.494479287141
H	-4.412959262436	0.064096168016	2.484425259434
C	4.874829493224	-0.442231561392	0.654265159607
H	5.924378059753	-0.609638035971	0.872962069161
C	-0.210099283820	-2.369212027976	-3.678501873548
H	-0.431546336676	-3.422359599417	-3.811934911102
C	0.068910147087	-1.544819360839	-4.770490664487
H	0.061740518088	-1.953454886419	-5.775670307827
C	-4.764569867783	0.134743918600	0.355682491712
H	-5.846238746233	0.141269855811	0.440706473807
C	0.366638632179	-0.197974440967	-4.556947219838
H	0.594356984749	0.468166994729	-5.381139141731

N	0.082861060151	1.821837752576	-0.394039182524
O	-1.078340153028	2.915691218298	1.636187500606
S	-0.181015294518	3.206385478429	0.504476896267
O	1.151864330091	3.771167616127	0.785095422258
C	-1.032220626588	4.246993689960	-0.656613805495
C	-0.287444927955	5.043539257671	-1.533815765871
C	-2.431212055349	4.242514013191	-0.683470913341
H	-2.992890762597	3.624103252194	0.007916759024
H	0.796356040628	5.040170066526	-1.494594435401
C	-0.964252158076	5.849675485265	-2.444212751113
H	-0.394233203474	6.475053554361	-3.125492796344
C	-2.368173775921	5.871820246728	-2.494238454024
C	-3.085884651672	5.057105058842	-1.603174120394
C	-3.087937545044	6.774095111977	-3.464870196006
H	-4.171949692847	5.063420444442	-1.627004011238
H	-4.073141963009	6.376443693650	-3.726417100922
H	-3.241560647618	7.767823441836	-3.023950161837
H	-2.511211002760	6.912016536391	-4.384686205945

$^{3}RC_{2b}$

Fe	-0.161407438409	-0.033547280838	-0.082852526358
S	-0.583562600358	-0.053446020786	2.218450421995
N	1.773690417026	0.180832597812	0.301086786469
N	-2.170584284619	-0.395968353819	-0.301968794942
N	0.314799170952	-2.109197080686	0.130675654162
N	0.289080524503	-0.459536390783	-1.967916632555
C	2.502999511561	-0.934501534684	0.496162059806
C	2.360065811300	1.393729235652	0.389268309097
H	1.710730914519	2.243328833132	0.219924981366
C	0.355531548013	-1.775705590718	-2.283158922153
C	-2.982784516520	-0.316161805598	0.779692701396
C	-0.470825004415	-2.693300285225	1.259478092168

H	-0.151023009781	-3.724140022303	1.455383938054
H	-1.517603103689	-2.723539654038	0.954156069401
C	-2.730489114907	-0.619467318235	-1.511897064855
H	-2.061104497666	-0.635552284709	-2.359713352248
C	-0.061564801639	-2.715551960735	-1.180538582638
H	-1.147960718319	-2.843170644745	-1.198702138520
H	0.383887054520	-3.707752595685	-1.304669953692
C	-0.306629644096	-1.855953123152	2.521041188207
H	0.705022154184	-1.909928881538	2.931399759370
H	-0.994779202555	-2.182294252696	3.304534739688
C	-2.402370231662	0.081885476786	2.111318192189
H	-2.846642640067	-0.483390640580	2.934852869489
H	-2.609102972965	1.142160992721	2.297024177314
C	3.709177261349	1.531417245157	0.678311543734
H	4.144470820698	2.522062899847	0.739826883136
C	3.863406300122	-0.864407229871	0.795899261324
H	4.426070621049	-1.778636349468	0.951342338434
C	1.795021432190	-2.256740044910	0.354425377402
H	2.231128522172	-2.802972128734	-0.487304812633
H	1.974118299707	-2.876571259691	1.237781301599
C	-4.092325237443	-0.814741706519	-1.687660490491
H	-4.480713202087	-0.994543470372	-2.683307733413
C	0.595035723853	0.473728449599	-2.887901817402
H	0.519612880047	1.504319130005	-2.563229846697
C	-4.359746718098	-0.518716438567	0.674026992699
H	-4.974429459983	-0.460750300302	1.565419027881
C	4.474591655110	0.382115618189	0.887337707761
H	5.532036235446	0.456560268179	1.118638733065
C	0.738991764081	-2.191123170410	-3.553785167089
H	0.792715901805	-3.250229502509	-3.779957061490
C	1.049566776256	-1.228636560389	-4.515363027103

H	1.350486304792	-1.532167206630	-5.512611011089
C	-4.925223391193	-0.776243903658	-0.570444599465
H	-5.994305636761	-0.934059587641	-0.666126248457
C	0.975417268408	0.122806676654	-4.178005702572
H	1.210218563327	0.899628173858	-4.896353153151
O	-0.405222421623	1.564216805273	-0.283065799183
S	-0.405909812771	6.444148361903	-0.076439171095
C	-0.626986944953	5.162855952870	-1.361580283674
H	-1.654302005532	5.245859463789	-1.726116257181
H	0.060662407165	5.327164042218	-2.196687950588
H	-0.475709662276	4.161705523824	-0.946867603750
C	1.343135897500	6.151298800525	0.365491664839
H	1.608036967664	6.877051919305	1.138607384816
H	1.482072006147	5.141357838520	0.762844095707
H	1.994155408911	6.299912682158	-0.500950006302

$^{5}RC_{2b}$

Fe	0.161726385833	0.009615341038	-0.247881841217
S	-0.000324578066	0.388957723822	2.297444657507
N	2.191780675143	-0.022918107962	0.127267510058
N	-1.987677118634	0.005483368030	-0.002406912632
N	0.350542437483	-2.058811901548	0.297506860032
N	0.201866810594	-0.920922458718	-2.131486601843
C	2.718392930278	-1.223846566670	0.445011632499
C	2.959353267977	1.085177645989	0.156810500317
H	2.462662390468	2.011285947647	-0.107743405712
C	-0.002096487843	-2.259179995232	-2.120700690634
C	-2.607185835184	0.274899392772	1.164160981946
C	-0.263602686801	-2.340028846322	1.633906632279
H	-0.044092376819	-3.373817314349	1.929125922781
H	-1.345403592045	-2.254791841748	1.521992998079
C	-2.736616905357	-0.186768694913	-1.112150627848

H	-2.197309444462	-0.369051098719	-2.032202058309
C	-0.355076142376	-2.835625911670	-0.771448754628
H	-1.431967153263	-2.742638512896	-0.601807441294
H	-0.103527997853	-3.899613179512	-0.708896072838
C	0.229254502725	-1.390296822207	2.716708627997
H	1.300507597629	-1.493883756152	2.908876801910
H	-0.290283001158	-1.593398042658	3.656669003150
C	-1.807270790110	0.609573817869	2.405581122010
H	-2.177839486042	0.049168482905	3.268761929702
H	-1.942062439013	1.671850579588	2.636680350959
C	4.299589012686	1.030603493936	0.511218210662
H	4.891283457200	1.938520004579	0.522679334127
C	4.052896466712	-1.346626235414	0.825561285381
H	4.453820415290	-2.319993611085	1.085891337548
C	1.815166656578	-2.416203560956	0.278541723033
H	2.040124890763	-2.870688624884	-0.691296607539
H	2.020965041667	-3.179687863995	1.034583401530
C	-4.122195277008	-0.154670126446	-1.097327624519
H	-4.672436032746	-0.320765467975	-2.016199471751
C	0.493740188523	-0.288911960075	-3.283910127306
H	0.647164277825	0.782042725024	-3.214928046291
C	-4.002291945397	0.311228199814	1.251402286433
H	-4.469134740405	0.521518137159	2.207811201047
C	4.852202182815	-0.205111540250	0.857087154238
H	5.894376430839	-0.279081669596	1.149825208080
C	0.080213436510	-3.010358061000	-3.287698023421
H	-0.078178042056	-4.082553879255	-3.256219104763
C	0.373547054153	-2.357885109729	-4.487269281118
H	0.443281804336	-2.924210562686	-5.410183230277
C	-4.770325819805	0.091258189620	0.113811366927
H	-5.853560975862	0.119226016576	0.169275765046

C	0.585000751707	-0.978291779196	-4.487264592247
H	0.819292106059	-0.441927594234	-5.399465615035
O	0.167010995269	1.553791585190	-0.748598898168
S	-1.874558790129	5.043894646142	-2.654738243258
C	-0.114536674666	4.906398203807	-2.180800325713
H	0.455319312059	4.747291751898	-3.100047177373
H	0.231533284109	5.827043763065	-1.701768368499
H	0.041305644685	4.055424727484	-1.511641062046
C	-2.619837050130	5.345610003948	-1.013052010233
H	-3.698173066060	5.446137858414	-1.159923499321
H	-2.428128524636	4.506849063255	-0.337339237526
H	-2.232062162468	6.269136870075	-0.573375365189

^{3}TS1$_{2b}$

Fe	0.071702092578	0.271527097795	-0.098988964376
S	-0.318942718833	0.320902165364	2.291144173222
N	2.058424183529	0.195374686293	0.227859578754
N	-1.963767391326	0.235126536020	-0.247899667521
N	0.285511893400	-1.918859906592	0.201305718890
N	0.410353219198	-0.383171845954	-2.018312390684
C	2.617672015957	-1.009242449774	0.456991242143
C	2.827364627380	1.302205281918	0.279117825820
H	2.303125038149	2.229197042102	0.082345315347
C	0.257749296127	-1.708356388291	-2.235277161628
C	-2.751338514307	0.333436952190	0.849236922998
C	-0.492073179784	-2.346632172331	1.391637003749
H	-0.293927580833	-3.401332102060	1.628912580327
H	-1.552932131268	-2.260658254816	1.146804489822
C	-2.564695961512	0.157487253685	-1.457592255959
H	-1.907251911939	0.112192445522	-2.314257223452
C	-0.254007476943	-2.501458232440	-1.054501751831
H	-1.346086107721	-2.432971411457	-1.017791286630

H	0.003720381694	-3.562684385013	-1.152381554031
C	-0.163559168786	-1.489048811229	2.609795975506
H	0.871438291806	-1.618329431231	2.938413788831
H	-0.808687456596	-1.748949414020	3.452995260773
C	-2.128277710418	0.565052071410	2.204995905699
H	-2.608006727476	-0.047434504516	2.973423571828
H	-2.282170216327	1.611201771713	2.492932333027
C	4.185227032964	1.248245003561	0.561632586890
H	4.765096795954	2.163932409736	0.590700738903
C	3.975481355605	-1.135410216462	0.756719213900
H	4.394319479499	-2.119059188337	0.941852115145
C	1.739424550583	-2.233917930086	0.340970349647
H	2.066326162041	-2.809066948329	-0.531989572547
H	1.899887963856	-2.889416067824	1.203554496603
C	-3.941821832413	0.137460024118	-1.623219263114
H	-4.359832223258	0.071465606910	-2.621176833129
C	0.846368448790	0.411447358333	-3.011689190116
H	0.954124579391	1.460124614013	-2.761801233778
C	-4.145589376638	0.300556054269	0.756004102455
H	-4.739186643947	0.368509537067	1.661389050315
C	4.770799603819	0.005156076912	0.809104762224
H	5.828299645210	-0.074145227347	1.040030239198
C	0.535009428046	-2.275833935238	-3.476341535496
H	0.411863201918	-3.343710854731	-3.620937885719
C	0.974754642155	-1.453012313484	-4.513767614516
H	1.197022136504	-1.874994668384	-5.488571290936
C	-4.753007080681	0.197264982856	-0.490349026983
H	-5.834492956946	0.172229422124	-0.575656614148
C	1.135711652419	-0.087515165469	-4.277650417617
H	1.483324394964	0.584025297731	-5.054576242421
O	0.166343645745	2.003602083339	-0.376659642599

S	-1.149741674053	3.612694103448	-0.520371629118
C	-0.383046997065	4.307742721138	-2.009080448219
H	-0.777122562828	3.754529943788	-2.863869298802
H	-0.645930921623	5.364592800259	-2.103349449009
H	0.700889873540	4.184700470426	-1.949088367236
C	-0.338362116349	4.588395031688	0.775563505215
H	-0.674748391670	4.192138040425	1.735808323870
H	0.745134973427	4.482297630519	0.687572158662
H	-0.631237574709	5.637900308742	0.687557481851

^{5}TS1$_{2b}$

Fe	-0.170858366735	0.140913827247	-0.303216455981
S	-0.390252891252	0.419459588156	2.255104597526
N	1.882781509512	0.260864966801	0.108386418401
N	-2.329537820994	-0.127861887934	-0.054324729115
N	0.243257437661	-1.968368758881	0.231850902391
N	-0.021689758827	-0.798331179115	-2.195992981391
C	2.510207163181	-0.890629175598	0.423038480910
C	2.547354142857	1.431233513289	0.174025011694
H	1.974094999090	2.312430735030	-0.088046963771
C	-0.074128480812	-2.150162671287	-2.196792795039
C	-2.971537364246	0.029953288768	1.120081259628
C	-0.356199375682	-2.323848009230	1.552178008643
H	-0.030517908928	-3.328912295493	1.852693784514
H	-1.439965876672	-2.355977820489	1.424783608394
C	-3.053070927526	-0.404118209211	-1.162091816358
H	-2.499270044546	-0.491306788545	-2.088287001965
C	-0.367483477178	-2.785685259019	-0.858156389944
H	-1.449664511772	-2.811174309564	-0.697404265346
H	-0.009613654699	-3.820647759788	-0.823980072728
C	0.009383633287	-1.335759252112	2.653506137964
H	1.081229155673	-1.330390360627	2.869459338234

H	-0.506319274699	-1.603275254747	3.579597788352
C	-2.211348474474	0.469368181221	2.353626144125
H	-2.530845588040	-0.098549128323	3.231810420813
H	-2.443811897118	1.521222512979	2.555003318414
C	3.878656085900	1.496175523685	0.560435411664
H	4.383255672182	2.454685375286	0.599977448457
C	3.842574253236	-0.896940299589	0.833650347791
H	4.324897318056	-1.834162129765	1.089720723408
C	1.730989971564	-2.166234617245	0.224906254410
H	2.013319122164	-2.573800111132	-0.750970436295
H	2.014594709960	-2.918727347737	0.967868127303
C	-4.429550073008	-0.571299610066	-1.138859282548
H	-4.959643001830	-0.796785561989	-2.057051359655
C	0.202270084398	-0.130864541764	-3.343663236487
H	0.234381370787	0.949915457673	-3.265605578560
C	-4.355815855616	-0.139758055352	1.219277562181
H	-4.839197275827	-0.019174514611	2.182970871950
C	4.534602345284	0.310808335781	0.900946427769
H	5.572437049486	0.326832316216	1.217555366685
C	0.098931876112	-2.878314120760	-3.369638521551
H	0.062462027741	-3.961927731150	-3.345817901963
C	0.323167917294	-2.189728338994	-4.563314956303
H	0.461882400630	-2.738064600277	-5.489428469434
C	-5.094430684313	-0.447148180713	0.081829418629
H	-6.169561269457	-0.579748193588	0.145140457492
C	0.377278041879	-0.795184579673	-4.552078846786
H	0.554856277620	-0.228661292611	-5.458976958846
O	-0.312242312200	1.699588846930	-0.795935775087
S	-1.108234505408	4.334286530214	-1.742306456420
C	0.491337461425	4.541416871237	-2.590844505173
H	0.369218183157	4.164047991071	-3.608922083056

H	0.772256493010	5.597803737089	-2.629258103041
H	1.271679227872	3.966881880094	-2.084804213933
C	-0.706124625425	5.028855704093	-0.105891710879
H	-1.615121694946	4.979646622838	0.498349120478
H	0.080058542027	4.442470532357	0.376944609373
H	-0.391215480813	6.072088608924	-0.198629499940

$^3PC_{2b}$

Fe	0.098763298201	0.325379093637	-0.080416378257
S	-0.370231431210	0.312474388578	2.425104198233
N	2.076383147635	0.224321278452	0.230714478565
N	-1.950390594207	0.341732243173	-0.214543417425
N	0.272545477854	-1.880487710909	0.195052885672
N	0.440628836893	-0.429771809766	-2.130800523822
C	2.616139385637	-0.991345708886	0.458129865625
C	2.869326573500	1.315404788313	0.293348723464
H	2.374160952312	2.258452489877	0.098714191661
C	0.279620419404	-1.759831074811	-2.268550552770
C	-2.758107103765	0.363131874166	0.872831714145
C	-0.510016771513	-2.322004908838	1.375645825053
H	-0.320414466400	-3.383916235368	1.589857423527
H	-1.569446386979	-2.226852355110	1.126984621039
C	-2.532752198269	0.300381075462	-1.436788810402
H	-1.856783477208	0.304588906942	-2.281180753734
C	-0.272903189250	-2.475035355255	-1.053977794906
H	-1.361229683486	-2.356550663140	-1.031595653947
H	-0.062218810996	-3.550289035506	-1.108345202663
C	-0.195707391111	-1.509118792347	2.629857499478
H	0.838697072660	-1.645600870400	2.958632727856
H	-0.841625974491	-1.828760336606	3.452052562155
C	-2.176791303730	0.535873121416	2.260030600688
H	-2.686759545959	-0.114397324891	2.976663882353

H	-2.355382457710	1.566372332992	2.589929556526
C	4.224626898861	1.236080772018	0.583880887407
H	4.819874337947	2.141606233978	0.621260624352
C	3.970087661205	-1.142679800654	0.763850026042
H	4.370184890305	-2.134880837407	0.945501259683
C	1.723387538454	-2.205523105120	0.335245806103
H	2.047251403467	-2.778628443009	-0.540399954203
H	1.879535536399	-2.866699807340	1.194618389771
C	-3.905776005727	0.252690471552	-1.628159982039
H	-4.306017105797	0.219860957639	-2.635113520298
C	0.906515446758	0.287486634468	-3.166181309774
H	1.025156784966	1.351788390551	-2.990025310382
C	-4.149971123451	0.298253708764	0.751796461774
H	-4.759826122970	0.304865484074	1.649044424849
C	4.786990866233	-0.018063221394	0.827153866893
H	5.841623254297	-0.118035300725	1.063262517717
C	0.577675335763	-2.416643837107	-3.461437554574
H	0.445570586447	-3.490890503500	-3.538249631955
C	1.048723287654	-1.669369376877	-4.541800550475
H	1.287519903836	-2.157367427704	-5.481515036502
C	-4.736046539498	0.239781566867	-0.507479264100
H	-5.815138696637	0.190509613930	-0.611519003676
C	1.218948389067	-0.292569316984	-4.393096548160
H	1.590688646383	0.322282316344	-5.205391926530
O	0.265621300858	2.324703764862	-0.519556262283
S	-0.861269540852	3.417151215934	-0.506374551980
C	-0.480073940677	4.443837927907	-1.952722656531
H	-0.684996096509	3.840641964724	-2.839302670135
H	-1.136076292551	5.317887871040	-1.937333298362
H	0.571339769935	4.738911536164	-1.920056110155
C	-0.351376699465	4.555228939303	0.812148558946

H	-0.453250993065	4.014106790761	1.755063653367
H	0.686181603055	4.857689722663	0.651260417252
H	-1.020559662505	5.419340683103	0.803502579846

$^5PC_{2b}$

Fe	-0.008194480858	0.296914452085	-0.338331709992
S	-0.302612004563	0.555174407816	2.255997140706
N	2.143522793851	0.217525497087	0.065322274086
N	-2.260450898616	0.303308482446	-0.125435992600
N	0.281331856855	-1.920920878511	0.236280497292
N	0.095166829806	-0.770910272333	-2.243157338297
C	2.626622795270	-0.979851706133	0.447865703775
C	2.931381613597	1.305933860564	0.154484946747
H	2.476574124412	2.237846020396	-0.163733514512
C	-0.065240580176	-2.110021115889	-2.196671519006
C	-2.894476336566	0.306718660507	1.064669271481
C	-0.338105294881	-2.203138858386	1.554270918082
H	-0.073792832209	-3.215328075943	1.895440505578
H	-1.422764880621	-2.179964074973	1.422508219485
C	-2.992217271082	0.107005762828	-1.244374931171
H	-2.443992473184	0.123306695168	-2.179897586178
C	-0.391839084847	-2.692483091546	-0.837907373719
H	-1.472576059128	-2.623278127837	-0.673743620376
H	-0.127486719692	-3.757295828429	-0.802695921989
C	0.064691521659	-1.209396673385	2.641069671064
H	1.140863758070	-1.226307764574	2.835553946295
H	-0.437888308712	-1.465797672133	3.577683544647
C	-2.128959220539	0.629262939513	2.335830936681
H	-2.471093897764	-0.000605891017	3.161805562052
H	-2.346972139267	1.663967031764	2.624123677005
C	4.236950689643	1.245180094147	0.628433258997
H	4.838291435242	2.146007793414	0.681563868939

C	3.922824255621	-1.118009006219	0.948677337382
H	4.282826784028	-2.093606597563	1.258900359824
C	1.746776889254	-2.196814953128	0.230694783098
H	2.008021892148	-2.609029707642	-0.750028169168
H	1.991502015162	-2.974800052288	0.963733488145
C	-4.363165050158	-0.110634561114	-1.228147158987
H	-4.898504787782	-0.264670177125	-2.158320041499
C	0.367210341852	-0.177927577767	-3.419775356422
H	0.502841850086	0.897573908555	-3.389141699774
C	-4.272227319718	0.082701810440	1.161445918542
H	-4.748148677882	0.082668448648	2.136700054532
C	4.738698958440	0.008412457861	1.037413722303
H	5.749711790347	-0.078014919594	1.423137360359
C	0.037615246012	-2.896428426057	-3.342744287176
H	-0.085522073612	-3.972066698001	-3.274160337197
C	0.304054620467	-2.279314905372	-4.564958937931
H	0.387530850026	-2.872983609013	-5.469754090650
C	-5.016120008698	-0.130466851640	0.005295395887
H	-6.085423535769	-0.306713510402	0.065689737421
C	0.474957264527	-0.894838433862	-4.606416329763
H	0.693514868906	-0.378607581121	-5.534415733103
O	0.184568347858	2.270927831964	-0.976174470072
S	-0.927987627317	3.265799473025	-1.453907104042
C	-0.030572097779	4.419319656404	-2.531197137375
H	0.267674127819	3.870666132829	-3.426201175529
H	-0.708685532231	5.232533292099	-2.802770647974
H	0.845166100234	4.798120110607	-1.998616420607
C	-1.207666769076	4.372228156349	-0.041563341175
H	-1.696528727618	3.787219838941	0.738869792633
H	-0.248978213312	4.760975517425	0.310143798337
H	-1.866539717536	5.182357266117	-0.364937745092

$^{3}RC_{2b,H1}$

Fe	0.022320419553	0.011237528475	0.545061578392
S	0.069097698152	-0.989929668881	2.660068772866
N	1.995810820241	-0.175732741215	0.448010016992
N	-2.021385896092	-0.001593380490	0.746249064744
N	0.079118142267	-1.978286018014	-0.244697985405
N	-0.010097892306	0.428412974564	-1.391636211180
C	2.495058024342	-1.330197760023	-0.033651957111
C	2.831774239318	0.795754320180	0.872116631358
H	2.356585681395	1.693201773151	1.247618573575
C	-0.297604488278	-0.604065943124	-2.220849053341
C	-2.569126426195	-0.341766837285	1.937823338104
C	-0.560785659870	-2.924004437973	0.719249302045
H	-0.427086865746	-3.960847425494	0.385941289383
H	-1.630858217334	-2.713649160484	0.731587940445
C	-2.843245313729	0.403940374870	-0.247558575126
H	-2.373714681795	0.707843733552	-1.172102294157
C	-0.677173190413	-1.889493812132	-1.529344763760
H	-1.746375423275	-1.881426735253	-1.297420931977
H	-0.489290198938	-2.760466759330	-2.164921867091
C	0.028614585010	-2.754899757240	2.113637167925
H	1.070377609040	-3.081327624005	2.170516680244
H	-0.534335984080	-3.330460657121	2.852418989114
C	-1.667521954521	-0.642819879877	3.106439282859
H	-2.049232175553	-1.468860095136	3.711911216452
H	-1.610297446861	0.237078661424	3.757491150003
C	4.209258970789	0.642856204466	0.826094679202
H	4.848302806115	1.446104294477	1.174122255368
C	3.870887659710	-1.549971065736	-0.099470822060
H	4.246202415017	-2.490742182439	-0.487746127697
C	1.507682250645	-2.358015254716	-0.521964175987

H	1.640014485208	-2.488179095683	-1.600215301509
H	1.723715788323	-3.331958635017	-0.073343298720
C	-4.223537974818	0.442025631815	-0.115537542224
H	-4.829689328247	0.772543022899	-0.950914775725
C	0.301761647908	1.636696903898	-1.895138155081
H	0.520871622912	2.410421995194	-1.169264281331
C	-3.951538729479	-0.344097398044	2.130517962515
H	-4.352380803434	-0.640336765769	3.093541259425
C	4.738616636023	-0.552098919373	0.333625738428
H	5.811968782663	-0.704247579096	0.288961821894
C	-0.276495158994	-0.440265362358	-3.601831205512
H	-0.500869188503	-1.282567198561	-4.246820485357
C	0.036381586082	0.813045270037	-4.130359218340
H	0.056369825735	0.960245215662	-5.205170808470
C	-4.792765248566	0.046499464548	1.093956466765
H	-5.868905397439	0.050702925168	1.231493261423
C	0.328723461446	1.868040849678	-3.265640508920
H	0.576418097376	2.857896414088	-3.635506229273
O	0.089205875222	1.544221723825	1.088982303534
C	-1.572908544479	6.250211584021	-4.151947600262
C	-1.139831359273	6.444806084511	-2.902942918452
C	0.283607378459	6.205982483849	-2.467750244179
C	1.160605262109	5.700300165900	-3.584953313253
C	0.727000774234	5.505845800971	-4.835466236914
C	-0.690512677761	5.769456145465	-5.275701388823
H	-2.615527785680	6.445098689665	-4.399135563403
H	-1.831607447192	6.797203503449	-2.139139194121
H	0.709363255501	7.133807159309	-2.050149532493
H	2.201936234746	5.499087959433	-3.336808395936
H	1.417895274947	5.147568528645	-5.597429681232
H	-1.118753825030	4.859438038090	-5.727435758069

H	0.304869692694	5.497085371660	-1.623298950734
H	-0.695086805622	6.505293660862	-6.097241759708

^{5}RC$_{2b,H1}$

Fe	0.490617379557	-0.584424524040	1.038304058826
S	0.251434084167	-1.928071089660	3.227047245358
N	2.421466992710	-1.308116786415	1.075813734411
N	-1.603472654382	-0.253982154710	1.449970024685
N	0.132829835673	-2.483992700003	0.102360998752
N	0.435739131053	-0.046882964943	-0.995267269492
C	2.619804352691	-2.508821364890	0.493209053351
C	3.433059934467	-0.686921693390	1.715280555486
H	3.193365370772	0.272023628326	2.159604525741
C	-0.083777051184	-0.980436346881	-1.825865169255
C	-2.217535267424	-0.674425364394	2.574293086358
C	-0.637636552585	-3.407509499309	0.995464511932
H	-0.703578407994	-4.398547958242	0.528840460320
H	-1.652050278291	-3.013740543910	1.074166444531
C	-2.302160915263	0.486246227273	0.559538341239
H	-1.759618011030	0.831836034709	-0.310594559103
C	-0.654832071200	-2.196538796234	-1.139244554023
H	-1.690419982265	-2.001009735981	-0.845209599891
H	-0.663506906518	-3.066105277930	-1.804578277997
C	-0.017084126487	-3.541322293249	2.378918167066
H	0.971002757424	-4.008616570530	2.352237018912
H	-0.652327636004	-4.164218489695	3.014173127668
C	-1.438780517890	-1.393971833143	3.654939642808
H	-1.996564290570	-2.258554174268	4.026299477909
H	-1.303219887367	-0.714594057296	4.503803912605
C	4.696856233824	-1.253184165397	1.796524672639
H	5.490310804339	-0.727945366181	2.315322968086
C	3.860948913332	-3.139560145237	0.550157016256

H	3.997925199209	-4.108006364003	0.081646968180
C	1.463731867206	-3.076881722440	-0.286414247312
H	1.637210221367	-2.857001514159	-1.344360555972
H	1.422577176998	-4.166864219044	-0.199415681548
C	-3.641378614302	0.799830368033	0.731958954329
H	-4.154279416336	1.391720728347	-0.017204418782
C	0.942599642560	1.096550843286	-1.493843145260
H	1.343674480987	1.796245389262	-0.769266224916
C	-3.569742012670	-0.404434323526	2.804050963962
H	-4.036525313098	-0.768424857041	3.712991132392
C	4.911102541967	-2.502305476778	1.208556131762
H	5.885756015051	-2.976041769354	1.263091649573
C	-0.110114468758	-0.786534465408	-3.202544185529
H	-0.522272193470	-1.551566186191	-3.851059334133
C	0.406144441400	0.401609687305	-3.723767076978
H	0.395914886276	0.574264220414	-4.794926580610
C	-4.292823274889	0.335042304520	1.875100305810
H	-5.342543829168	0.551637306137	2.043761836159
C	0.941711114940	1.357374460627	-2.858959660419
H	1.354662013876	2.288052327181	-3.230502240507
O	0.902004184543	0.859289431807	1.654872363814
C	-2.017508996705	7.898260894121	-7.647077887185
C	-2.175941872784	7.283764141117	-6.471193688444
C	-1.477532168560	7.712151164278	-5.205837308553
C	-0.576667841555	8.903550932176	-5.409335208740
C	-0.418139020354	9.517843387730	-6.585287271349
C	-1.120176253843	9.092421869314	-7.849542660740
H	-2.554518695200	7.536290476753	-8.522847886243
H	-2.841423110631	6.424771219256	-6.395495009862
H	-2.222496513017	7.934924806253	-4.423443518155
H	-0.037988484872	9.264267870692	-4.534068791584

H	0.249015193238	10.375411948404	-6.661616039132
H	-0.377998208172	8.874887195679	-8.635927722454
H	-0.898120510102	6.869606226197	-4.792208407563
H	-1.703406923192	9.935365266849	-8.256748394638

^{3}TS1$_{2b,H1}$

Fe	0.282445133936	0.513472436814	-0.208516910989
S	0.255977285872	0.260049981221	2.119517114033
N	2.234309988022	0.085763427497	-0.195479235563
N	-1.750900284014	0.824323750541	-0.044710074488
N	0.102163477212	-1.608639445556	-0.307444209335
N	0.238991860805	0.288370678820	-2.183343619824
C	2.583050988331	-1.213181413563	-0.251711989087
C	3.191628456312	1.030262315859	-0.092247397375
H	2.829105798683	2.049930775627	-0.050579076950
C	-0.165946779399	-0.922873433041	-2.634574381693
C	-2.306381118511	0.933770407445	1.187275924290
C	-0.634039881270	-2.107682664084	0.893495786448
H	-0.629860174449	-3.204782706675	0.917081672423
H	-1.671323752735	-1.782183353101	0.803997858710
C	-2.565395677725	0.911386916820	-1.119159869838
H	-2.091129943017	0.859023071726	-2.088644871052
C	-0.660055953598	-1.865230694878	-1.564916934025
H	-1.718551156843	-1.670626077460	-1.367760817403
H	-0.575583713941	-2.911400611544	-1.877503426679
C	-0.021727748768	-1.565007006617	2.179704876518
H	0.969085973547	-1.983347215419	2.378116438696
H	-0.652400548531	-1.796261821021	3.041798289156
C	-1.406134127376	0.963573439044	2.396155242431
H	-1.865356064806	0.468739484008	3.255349890923
H	-1.213411288480	2.003264682953	2.684781687951
C	4.539862268545	0.705518189931	-0.042118311126

H	5.278985284480	1.494340654199	0.039864393967
C	3.919306377728	-1.610741128465	-0.198216612453
H	4.168137291872	-2.665808696555	-0.241596945905
C	1.471651023389	-2.219751463245	-0.410102451516
H	1.572793818550	-2.699912666591	-1.388244810918
H	1.577255123435	-3.020076130382	0.328509863480
C	-3.942047818512	1.061506990512	-1.018908873981
H	-4.540177321480	1.115962519867	-1.921162184137
C	0.664475732069	1.224154747492	-3.051680559375
H	0.977788574475	2.165818508752	-2.616672719922
C	-3.683797452836	1.079419233545	1.361453950177
H	-4.088567453783	1.148880038196	2.365139140579
C	4.910804608677	-0.639833947091	-0.093318987452
H	5.956280001056	-0.927800139325	-0.051253237389
C	-0.156715969563	-1.225944725958	-3.992357162790
H	-0.479029264096	-2.205169094505	-4.328975470946
C	0.271722142950	-0.255307264544	-4.898999139568
H	0.286090691133	-0.471000905488	-5.962298560866
C	-4.517182185826	1.136543444854	0.248242169729
H	-5.590207494912	1.242817242551	0.368991612397
C	0.687973352174	0.987649310011	-4.421879236469
H	1.031662317109	1.765454453255	-5.094029885694
O	0.638336983342	2.189258694833	-0.150127968099
C	-2.105770854915	4.590379836842	-2.659913203142
C	-1.959196228969	4.294335824926	-1.353658995233
C	-0.658608702436	4.386648228937	-0.666072408682
C	0.417504458235	5.044462768024	-1.435082619645
C	0.290502225871	5.338018182713	-2.743497835159
C	-0.973448512746	5.083259971507	-3.513776774543
H	-3.081213963946	4.501149302021	-3.133104527656
H	-2.817620045277	3.967935276742	-0.772139354905

H	-0.725627918896	4.710767632035	0.379779524442
H	1.347064298264	5.263437493733	-0.914717700515
H	1.114315324143	5.801924247723	-3.281477235918
H	-0.777209121118	4.363414911199	-4.328688299125
H	-0.213929051319	3.235749382185	-0.455516880649
H	-1.278849286124	5.999471150147	-4.046139668271

^{5}TS1$_{2b,H1}$

Fe	0.003522818271	0.630939690730	-0.461194286651
S	-0.084479169531	0.253212307884	2.096986397382
N	2.082283648572	0.236132218311	-0.181253129579
N	-2.180932743370	0.624464528794	-0.110894784409
N	0.057986714558	-1.644735179195	-0.484725581317
N	-0.101913935275	0.137403628269	-2.537095691408
C	2.488446924926	-1.047997441907	-0.208551955814
C	2.959032516204	1.212557892305	0.126692244161
H	2.556961575168	2.218815222235	0.132547854673
C	-0.395814596268	-1.145657735392	-2.848053923396
C	-2.740404946367	0.563209389841	1.113585087328
C	-0.541332912039	-2.203896295182	0.758209702029
H	-0.390378329343	-3.291567916959	0.795476141512
H	-1.617741504623	-2.026105079496	0.715586489090
C	-2.985883808952	0.751729508463	-1.188983371420
H	-2.492659428362	0.825410509774	-2.150261652081
C	-0.737149154510	-2.040955378060	-1.679063116584
H	-1.797356393026	-1.924015925922	-1.433637613699
H	-0.577590047828	-3.095161790906	-1.933485249625
C	0.036072429385	-1.585189107500	2.026124028591
H	1.103070886503	-1.793315761517	2.141601818523
H	-0.471071447910	-1.993297546967	2.904332238013
C	-1.868119800565	0.548597193979	2.351823995806
H	-2.241799918245	-0.172868317195	3.083949859414

H	-1.915052447861	1.534598539610	2.827717210944
C	4.287651884157	0.939425402258	0.422016363559
H	4.965662396856	1.750076677523	0.663387209604
C	3.805069690927	-1.396941121004	0.092480035701
H	4.106614009588	-2.438806101968	0.072929977007
C	1.479327785677	-2.075593575345	-0.662975897699
H	1.650066659521	-2.248706650976	-1.730699798514
H	1.651298655263	-3.035987629899	-0.164504208613
C	-4.369257992050	0.789214955961	-1.096179468592
H	-4.966892547018	0.888242455404	-1.995076098641
C	0.182527280403	1.017819879858	-3.515181698324
H	0.412711250470	2.029041851837	-3.196232881225
C	-4.128650297973	0.580911353689	1.281103345122
H	-4.545536545435	0.518106503052	2.280660294793
C	4.715052590362	-0.390427320189	0.409608078334
H	5.744257258442	-0.640828767697	0.646029060256
C	-0.413869749281	-1.582918788891	-4.169290054286
H	-0.643343177939	-2.619025596913	-4.393357520170
C	-0.128362545483	-0.670680923011	-5.186562617167
H	-0.136543516933	-0.991094770141	-6.223376282651
C	-4.954117812367	0.691377684409	0.167335508782
H	-6.032939751534	0.707627490925	0.283283429897
C	0.176266842068	0.650374399147	-4.855955445098
H	0.408671291577	1.385298672521	-5.618152295917
O	0.156343451659	2.299481172378	-0.559024405253
C	-1.393858392104	5.921334938918	-2.564930316351
C	-0.843733979151	5.582711871667	-1.383651950236
C	0.492450169160	4.946557310500	-1.280422277980
C	1.255358080188	4.881072805144	-2.552030364285
C	0.712400477354	5.213399894778	-3.738954613508
C	-0.688168757227	5.731901315394	-3.874591800623

H	-2.381986224724	6.374824913425	-2.596344169699
H	-1.390011140308	5.754577971362	-0.458999563811
H	1.088860878746	5.336898138895	-0.442893118258
H	2.283204106915	4.528579998961	-2.502971953403
H	1.303320195276	5.138284058334	-4.649060795639
H	-1.276058921430	5.058328339006	-4.523381255062
H	0.332085242318	3.846641778261	-0.927881345185
H	-0.680261775483	6.682280258431	-4.434941818352

^{3}INT$_{2b}$

Fe	0.329870962651	0.447422805156	-0.148812349122
S	0.278144835011	0.108470862337	2.169251964462
N	2.268408875139	0.019078171953	-0.119123968882
N	-1.695303153198	0.821617948614	0.004512004441
N	0.120037181981	-1.632938617431	-0.343156202944
N	0.299264589300	0.325726656416	-2.129577959599
C	2.606688627122	-1.278485154806	-0.236270459477
C	3.227490244560	0.951999508315	0.046081549764
H	2.871345702243	1.971027636379	0.130329795895
C	-0.124595728144	-0.855332732776	-2.639485843316
C	-2.260474826864	0.890496427245	1.236130342949
C	-0.629600241786	-2.182684725558	0.830329328374
H	-0.639655792290	-3.279159800096	0.792972895204
H	-1.661696690552	-1.837632444879	0.753845976407
C	-2.492104305273	1.009608266566	-1.071975262412
H	-2.010258457063	0.982236232771	-2.038904131534
C	-0.643177962122	-1.829821313169	-1.613532163099
H	-1.698248892936	-1.622604882984	-1.411778079340
H	-0.575053290888	-2.864924423772	-1.963080984595
C	-0.013339668070	-1.714756241498	2.142061779280
H	0.974210947920	-2.149890040241	2.318763372738
H	-0.646149852833	-1.984189057488	2.991331711660

C	-1.380371333609	0.815318992379	2.458785924295
H	-1.864537206757	0.268254332739	3.271372237318
H	-1.179527507735	1.828300387072	2.825865973705
C	4.572357275595	0.612621244910	0.101113075697
H	5.316488924042	1.389658844633	0.233896094516
C	3.938117994716	-1.689642515328	-0.181412425538
H	4.180853958952	-2.742808651725	-0.274629903742
C	1.484945482545	-2.259113237589	-0.466754419676
H	1.588902365684	-2.680181160261	-1.471310514083
H	1.567456428100	-3.103791018117	0.223502327814
C	-3.858816211927	1.230586347212	-0.975973777307
H	-4.441699161178	1.369107909500	-1.879078072001
C	0.757199042871	1.290165058131	-2.948828213466
H	1.083804855408	2.205188967382	-2.469365008629
C	-3.630668273330	1.096251016285	1.403915497557
H	-4.044305835150	1.129502152011	2.405875281740
C	4.933839362268	-0.731535569949	-0.011751217964
H	5.976480183535	-1.029221688521	0.032269819819
C	-0.106142139495	-1.097121550190	-4.008838731331
H	-0.444893924426	-2.052891596092	-4.392710384319
C	0.354532251806	-0.096090858382	-4.865463421406
H	0.377525181622	-0.264001319619	-5.937119510534
C	-4.444910765742	1.263239620270	0.287873625277
H	-5.512043415849	1.421063364097	0.404262888549
C	0.792509135303	1.114330708786	-4.328000017170
H	1.162464113961	1.913642812387	-4.959734891921
O	0.693450802807	2.191012703510	-0.006121072136
C	-2.318929646693	4.643659894348	-2.668944634002
C	-2.119075423680	4.752428288532	-1.319697390743
C	-0.833301811825	5.035694981547	-0.781712634148
C	0.270351500368	5.220551986878	-1.661534022766

C	0.124101680774	5.121982289626	-3.018188839325
C	-1.202230749669	4.821414841546	-3.658905050346
H	-3.310227936816	4.431208458072	-3.061362072923
H	-2.958637202091	4.625148313905	-0.640150117709
H	-0.703379163039	5.156003851928	0.289646935444
H	1.246617446861	5.450364027239	-1.240729160593
H	0.977292940586	5.274458449113	-3.674513776097
H	-1.118975484646	3.923952683683	-4.301529810176
H	-0.058900292274	2.755172899162	-0.275072546204
H	-1.461644545785	5.618489657835	-4.381936362333

^{5}INT$_{2b}$

Fe	0.609815167315	-0.164424991458	0.392876148434
S	-0.166611193765	-0.638860474327	2.891149964120
N	2.573715653647	-1.002618232164	1.050391041074
N	-1.679088163445	0.058777942322	0.272235870714
N	0.337965289124	-2.569129567260	0.327004711127
N	0.834545986645	-0.789247062138	-1.741556159035
C	2.721300002281	-2.334698967000	1.140784427081
C	3.521901464106	-0.199851588923	1.565809549596
H	3.333237370224	0.864251509319	1.462826643724
C	0.468082254940	-2.039271234513	-2.087265426900
C	-2.491448718465	0.037001916575	1.344683617634
C	-0.587920673764	-3.017707699841	1.390426623135
H	-0.612030236016	-4.117583441001	1.447013412207
H	-1.592924046636	-2.687828871653	1.115970171178
C	-2.221327840645	0.294510297025	-0.940773578719
H	-1.526102357545	0.302731476241	-1.773377446174
C	-0.234144757796	-2.843561941933	-1.012344400657
H	-1.288596017295	-2.548274623062	-0.988772402403
H	-0.204945693783	-3.914681845589	-1.256098906271
C	-0.238181456160	-2.474787233391	2.774051208288

H	0.750575740153	-2.806173435915	3.104037494655
H	-0.963594851833	-2.840860810181	3.506236490995
C	-1.920993663297	-0.146410350561	2.741730373905
H	-2.535046406034	-0.851246658354	3.310626886018
H	-1.981464878067	0.811783576161	3.270440555184
C	4.661589795471	-0.694354366633	2.192204119513
H	5.403702528258	-0.010877478279	2.590718705506
C	3.827429531500	-2.909971086223	1.771803632185
H	3.912921492381	-3.989964271032	1.839960818959
C	1.680716874254	-3.194949563565	0.449846101672
H	2.054097035165	-3.395158671579	-0.560822144365
H	1.608206290484	-4.171354945276	0.946313260230
C	-3.579458212946	0.506930699151	-1.141796201631
H	-3.959968116151	0.687112829081	-2.141252237279
C	1.423967521494	-0.001852784749	-2.657390027040
H	1.700797618523	0.988385917645	-2.308358235763
C	-3.873833062730	0.228000009469	1.222623166415
H	-4.500948526145	0.192469123535	2.107900012123
C	4.811881048834	-2.078249820396	2.301915395546
H	5.680535920182	-2.503224370551	2.795483062051
C	0.688279744310	-2.539714888888	-3.370668899491
H	0.392338558986	-3.554896551957	-3.614813696929
C	1.292193895745	-1.718085063098	-4.322911634953
H	1.473026712829	-2.086526880762	-5.327893811502
C	-4.425905384523	0.466277144690	-0.031780723185
H	-5.495682024127	0.615116918312	-0.141690015024
C	1.667586697054	-0.423897892624	-3.961777737812
H	2.145207221485	0.245241021701	-4.669259607066
O	1.217600465050	1.625038343226	0.324017610869
C	-1.656000632785	5.175129046740	-0.254562222681
C	-0.372631417325	5.453885240918	-0.649961489491

C	-0.027812272888	5.308687304951	-2.011381997445
C	-0.946935032712	4.888668262556	-2.996777142505
C	-2.237554316944	4.608403709620	-2.626714215909
C	-2.667028724796	4.734706405918	-1.226271591105
H	-1.955964074805	5.275575045247	0.783961371646
H	0.375496967921	5.784366018738	0.061482943637
H	0.991488305073	5.532889315933	-2.314392375851
H	-0.626311991681	4.797597726699	-4.028247630041
H	-2.972694404833	4.286502534024	-3.357943581996
H	-3.110765907146	3.776216092749	-0.890924132565
H	0.516902038624	2.254782539170	0.096868379924
H	-3.551870694972	5.398621637163	-1.167864457554

^{3}TS2$_{2b,H2}$

Fe	0.050570844584	0.433053770263	-1.061726751798
S	0.154017009210	1.182823192362	1.252776616281
N	2.037272012667	0.315186538491	-1.110273533666
N	-1.994386426958	0.571825446218	-0.820129505248
N	0.229304611472	-1.547364909062	-0.233129102239
N	-0.050308989627	-0.704022235610	-2.778640980937
C	2.602590769185	-0.819586980947	-0.656088029934
C	2.820733492099	1.324944066921	-1.539326918414
H	2.290550689823	2.199980731747	-1.893426479971
C	-0.241384008934	-2.027521815482	-2.590615403618
C	-2.519662515101	1.075681232719	0.323173766802
C	-0.357500480998	-1.586631502900	1.138062372542
H	-0.174672558045	-2.563807502577	1.605112341270
H	-1.437790788422	-1.466965664486	1.037129199869
C	-2.848854506434	0.171280291841	-1.789768682404
H	-2.396265268186	-0.203552046029	-2.697531405713
C	-0.535395671564	-2.426938189811	-1.164776346764
H	-1.601616499397	-2.283779529703	-0.963074042162

H	-0.308902288639	-3.484386687936	-0.990024338058
C	0.209300976898	-0.487313853992	2.031061776993
H	1.267214654780	-0.644670266261	2.259275635741
H	-0.327515884476	-0.453150792628	2.982529098016
C	-1.606053664511	1.654578923093	1.378444956745
H	-1.967852285931	1.423860057652	2.384081174200
H	-1.595959433065	2.747082617256	1.285818717860
C	4.206228499904	1.237410278544	-1.525872565519
H	4.801380624332	2.071527323838	-1.880221704631
C	3.989000658763	-0.972619213034	-0.607376353635
H	4.415963413088	-1.896517770674	-0.231373100428
C	1.679123938641	-1.935999138308	-0.227506413908
H	1.822903862015	-2.786663744402	-0.901488448060
H	1.961282080062	-2.293436538552	0.767791034126
C	-4.229334085798	0.224357633063	-1.658591052482
H	-4.858090799478	-0.114818655849	-2.473876558526
C	0.189141546895	-0.226881818514	-4.012145177174
H	0.342382088615	0.844440254106	-4.079831697411
C	-3.900171526685	1.140789612219	0.528852825963
H	-4.279646763182	1.536803533813	1.464709666691
C	4.801601697541	0.068122122782	-1.047989220029
H	5.881928706935	-0.031462723399	-1.019188335054
C	-0.202788779459	-2.923449863853	-3.655338936713
H	-0.351246442216	-3.983063507024	-3.477386692936
C	0.031465135805	-2.432433095404	-4.940751426434
H	0.065459521553	-3.111888451691	-5.786231936296
C	-4.768957150838	0.709049713041	-0.467940597024
H	-5.842924043763	0.754938794158	-0.319613119458
C	0.230970944168	-1.063603152229	-5.123226423803
H	0.421204018440	-0.645887606693	-6.105437178382
O	0.089324500012	2.111988730175	-1.815963961156

C	-0.593680510399	5.856314683253	-1.558137365279
C	-1.561505324743	6.401737599074	-0.755152714881
C	-1.479751779305	6.281265943703	0.653638918308
C	-0.384837575330	5.618119106261	1.258342103944
C	0.604189580734	5.060710820829	0.487409422322
C	0.539155260224	5.078204936017	-0.995990363434
H	-0.653517832546	5.958165187613	-2.638314093654
H	-2.394397987923	6.943396317292	-1.194522891545
H	-2.252526664370	6.720847086836	1.276580282597
H	-0.324618278225	5.570256914751	2.342038348557
H	1.452117189053	4.566511803643	0.953506840288
H	0.462232379155	4.010071796410	-1.354350182400
H	-0.815714207905	2.379266370685	-2.052723811344
H	1.501098315801	5.391462826378	-1.438341256595

$^{3}PC_{2b}$

Fe	-0.047515326805	0.566980045238	-0.988656416488
S	-0.034578174242	1.262250003144	1.383619669163
N	1.946747866530	0.460621257715	-1.033504046386
N	-2.091990386293	0.654707852884	-0.787058484042
N	0.202912808995	-1.511679838315	-0.165055349185
N	-0.085134461617	-0.633642745469	-2.764816446192
C	2.537199825435	-0.667353084917	-0.584321065501
C	2.724126047982	1.475878361936	-1.468129537605
H	2.205753823198	2.349087013578	-1.841273078443
C	-0.215280983553	-1.960006073393	-2.552223784433
C	-2.674725933806	1.064218844513	0.367177198957
C	-0.393225830314	-1.560657061711	1.190682141499
H	-0.177670381584	-2.521013662870	1.681718600299
H	-1.477958329037	-1.487208012324	1.082346496729
C	-2.900823995423	0.273106032260	-1.804528890801
H	-2.404004592476	-0.034607958193	-2.714527373076

C	-0.523819647843	-2.373951942906	-1.127799587115
H	-1.596699264999	-2.249845867027	-0.946559384288
H	-0.290272814318	-3.435363862647	-0.977686529426
C	0.118369181651	-0.434857651966	2.090734071029
H	1.185970511868	-0.536020874918	2.305544353919
H	-0.407194915179	-0.451803604214	3.049093015659
C	-1.825105739902	1.608275367544	1.492571555480
H	-2.198320525490	1.271865605220	2.463679375073
H	-1.888589250937	2.702838656493	1.489297394124
C	4.110166391521	1.416541152557	-1.456627311674
H	4.687463203170	2.260544542639	-1.816451003019
C	3.927321456622	-0.792140090183	-0.538249333418
H	4.368362916403	-1.710751342461	-0.165123646493
C	1.658051661057	-1.823263084266	-0.156201998244
H	1.852864674631	-2.667414350068	-0.827080041798
H	1.963137883320	-2.163459447069	0.839354679008
C	-4.285339184780	0.262181781794	-1.716983774830
H	-4.873279637913	-0.054949066072	-2.570725892272
C	0.162347266388	-0.185011650231	-4.008415820734
H	0.267231944641	0.889707281625	-4.115597183095
C	-4.063100217331	1.059546743674	0.528808015861
H	-4.487658524220	1.382578446090	1.473416221334
C	4.725577471586	0.259555458145	-0.975617100612
H	5.807254620838	0.177508976457	-0.945829901557
C	-0.105787262868	-2.879664100947	-3.593321325674
H	-0.206886936283	-3.940458008915	-3.389689333984
C	0.137538319428	-2.414245394092	-4.885944533477
H	0.226331351379	-3.113365682877	-5.711355278780
C	-4.882116603455	0.654316619419	-0.519350742811
H	-5.961166073072	0.648397718277	-0.404647233381
C	0.275686570847	-1.042650150426	-5.098606041958

H	0.473000921176	-0.639265354904	-6.085525798565
O	-0.195685252024	2.473657738897	-2.068752569199
C	-0.395813499708	5.901188664632	-1.576467627736
C	-1.433001806495	5.987655217266	-0.642985230024
C	-1.202476569064	5.641246292345	0.692402669797
C	0.062677482686	5.201127857232	1.094819587063
C	1.100990476039	5.109359524010	0.161806702015
C	0.873525503874	5.462723264463	-1.174575302264
H	-0.569774060007	6.181432575379	-2.611650437191
H	-2.415356190453	6.332097423085	-0.953594570081
H	-2.005926572097	5.720721402006	1.419694242538
H	0.242613756147	4.939047819283	2.133665557658
H	2.086484578869	4.775880058347	0.474703872287
H	0.107464926141	3.302577730940	-1.644863198643
H	-1.114629097221	2.655787906414	-2.332042547110
H	1.685611598391	5.417080727881	-1.895795667889

^{5}PC$_{2b}$

Fe	0.124541528733	-0.241021581414	-0.293313036789
S	0.576417803055	0.273299438912	2.258959988910
N	2.289587463937	-0.440843005226	-0.434662415460
N	-1.938513596592	-0.076320704374	0.492429383668
N	0.453832734636	-2.396910210190	0.369954736894
N	-0.242058293374	-1.392693776583	-2.130678387715
C	2.812698696240	-1.639511907359	-0.116811355051
C	3.114337820013	0.593902343912	-0.692277164641
H	2.632193554494	1.531602445637	-0.944716624055
C	-0.436255208649	-2.713615453022	-1.915489786912
C	-2.246486359374	0.192500538055	1.777545880909
C	0.132891518796	-2.516590905031	1.813844964249
H	0.378651965416	-3.524239893295	2.180157206817
H	-0.946272329206	-2.387047180617	1.925774518349

C	-2.945510112952	-0.307172309264	-0.381542364094
H	-2.649354975430	-0.510811704057	-1.404739042694
C	-0.503907159936	-3.161259589557	-0.467788655431
H	-1.511125339492	-2.970022167833	-0.082307167906
H	-0.331828843734	-4.242610170453	-0.396593822333
C	0.871042695647	-1.498522408869	2.682516817337
H	1.956572784788	-1.614460034423	2.613047860002
H	0.597836849621	-1.645687150559	3.730977427618
C	-1.157106550428	0.531659239756	2.778205668002
H	-1.325089767770	-0.004366314997	3.716859856867
H	-1.218594939910	1.599595741723	3.014897129008
C	4.496704230131	0.473687919687	-0.641113369964
H	5.124255275981	1.330883190774	-0.857737696653
C	4.193261504950	-1.833025945664	-0.030051015325
H	4.586646921964	-2.807966840409	0.238845965745
C	1.855485446017	-2.800302741244	0.070714369387
H	1.857986554967	-3.377662765189	-0.860295191386
H	2.232530346002	-3.476828985017	0.846860455811
C	-4.283378884877	-0.292544477900	-0.017519548096
H	-5.048780622427	-0.486611101389	-0.760212721474
C	-0.190143786295	-0.936475527199	-3.395758685827
H	-0.012586549984	0.127117768868	-3.516965519478
C	-3.574351249123	0.214120319255	2.218361054789
H	-3.787428730266	0.425694887316	3.261083239278
C	5.045080676212	-0.764229334039	-0.297456438710
H	6.121012033554	-0.894103596747	-0.236765581935
C	-0.590963068863	-3.609976316610	-2.971419465397
H	-0.733890841291	-4.665747397569	-2.767444728812
C	-0.553184304718	-3.130086612185	-4.280720659363
H	-0.672108127942	-3.812126849963	-5.116570733667
C	-4.603785372188	-0.030993431505	1.315951154307

H	-5.637498058097	-0.017909337749	1.646432445660
C	-0.346725272303	-1.768252761483	-4.499584747376
H	-0.299745855748	-1.354815344577	-5.500691237415
O	0.155552319998	1.749871692700	-1.145035653806
C	0.264112237546	5.579781215005	-1.230109588606
C	-1.117308917930	5.559047931669	-1.444336286479
C	-1.962717835657	4.943300761694	-0.515720572266
C	-1.426475042046	4.349199558426	0.631013771190
C	-0.041853126042	4.370377052811	0.848285137017
C	0.803384113419	4.985901857750	-0.084688809435
H	0.918930240599	6.059540130576	-1.952010968903
H	-1.535265199113	6.022443201801	-2.333734594769
H	-3.036175170699	4.929963896078	-0.682463629648
H	-2.083191409497	3.878005390394	1.357087451477
H	0.374300270132	3.926464855419	1.749074727037
H	0.133114836373	2.498365404866	-0.513489157954
H	-0.517494340607	1.965035582892	-1.813642065563
H	1.875974259334	5.007814067586	0.086777551056

^{3}RC$_{2c}$

Fe	-0.151606099781	-0.270634433766	0.023968323913
S	-0.857178513220	-0.651443734302	2.216951210636
N	1.383194765353	-1.518109084932	0.350965239056
N	-1.845228618443	0.882799339849	-0.148771105294
N	-1.156329868778	-2.056954103926	-0.481682631919
N	0.178999223836	-0.337319298267	-1.954482405657
C	1.162892443211	-2.821864004806	0.084032659865
C	2.567598114262	-1.143681226155	0.882264711102
H	2.691529643334	-0.089827168333	1.093777910942
C	-0.661045206711	-1.129563232173	-2.662955981398
C	-2.666873199180	1.060016840674	0.912312150748
C	-2.216665043040	-2.380236922316	0.517542303264

H	-2.621353302106	-3.382741543711	0.332052986484
H	-3.031050401311	-1.665792479270	0.384129454047
C	-2.118854487131	1.556182034896	-1.290838246719
H	-1.417697509426	1.432996061257	-2.103236326266
C	-1.727694727653	-1.804071308820	-1.838952862436
H	-2.594916744706	-1.147029348376	-1.729595646457
H	-2.072306858234	-2.734077605335	-2.303226836485
C	-1.650054601710	-2.292605861460	1.924561559849
H	-0.866505565752	-3.030571681809	2.113395123266
H	-2.426191515387	-2.434166264363	2.680564805445
C	-2.323777840982	0.442639393736	2.243848085250
H	-3.164594633534	-0.120541623531	2.658540611362
H	-2.070958152267	1.237605278011	2.949142627971
C	3.571364529788	-2.061785400137	1.158502729205
H	4.506604420063	-1.715595782654	1.583337111822
C	2.121991137614	-3.797175261025	0.352710133057
H	1.906195281703	-4.837547729634	0.134494022096
C	-0.143761811693	-3.167910394399	-0.573246009410
H	0.049162403104	-3.376158087726	-1.629726254218
H	-0.563137113193	-4.086979478943	-0.153363550107
C	-3.226266288243	2.376082905528	-1.440299155099
H	-3.379771174119	2.885857526773	-2.385988595640
C	1.173243515723	0.318008006556	-2.581118750356
H	1.807938067288	0.933349588821	-1.955391080379
C	-3.807972793661	1.862127321558	0.825543279638
H	-4.447096564439	1.968300173596	1.695376865315
C	3.345290890476	-3.412920607497	0.893956061559
H	4.106764495620	-4.155357085910	1.108852751439
C	-0.527013698836	-1.277692942375	-4.039062449881
H	-1.208043103359	-1.924736753498	-4.580422676385
C	0.494298122378	-0.589825538396	-4.695891696181

H	0.617495523866	-0.690748079334	-5.769052466894
C	-4.099424308545	2.523002120725	-0.361975306001
H	-4.981535217864	3.150066492331	-0.439798948280
C	1.359012969034	0.216529950647	-3.955694922961
H	2.169927834572	0.759482945048	-4.427210372509
N	0.762451860143	1.180018828661	0.341975052613
O	-0.068209827924	2.479866836885	2.392047412909
S	1.067857415782	2.369873982405	1.457308631365
O	2.407096158510	2.089181883404	2.007337416516
C	1.138329730157	3.839422256686	0.459566603326
C	2.356974271287	4.213214240714	-0.120268626953
C	-0.023423978597	4.590154926467	0.258624126021
H	-0.957354925783	4.295735186050	0.724603891653
H	3.252210953821	3.627621158904	0.058178454075
C	2.401584712403	5.358211692591	-0.908303459385
H	3.345598545865	5.659987864771	-1.353649499567
C	1.250734825068	6.134867241417	-1.131364026355
C	0.045126915194	5.734172179882	-0.535217056552
C	1.321096233566	7.370607976881	-1.993828132880
H	-0.851018834946	6.329335756295	-0.686060824404
H	0.372766582548	7.914824023883	-1.993407262331
H	2.107626919583	8.049337449863	-1.644597816193
H	1.561031394344	7.108282298050	-3.031523932626
S	-2.353839086206	4.002168037430	-5.008634286868
C	-2.897081449385	5.745537271482	-4.923318643063
H	-2.735055984995	6.156569741374	-3.922795312789
H	-2.367621030258	6.353459566730	-5.662476477116
H	-3.966623161752	5.762513338769	-5.147699645034
C	-0.580537045277	4.202717069405	-4.611665632604
H	-0.126339170219	3.208986298117	-4.647360568995
H	-0.088488980473	4.845568822371	-5.347263773854

H	-0.448383460375	4.620443157689	-3.609120051309

^{5}RC$_{2c}$

Fe	-0.096749797975	0.095766482810	-0.062763047625
S	0.306636593602	0.027840763396	2.502696436128
N	1.969180862276	0.036877579531	-0.182032437208
N	-2.119182063254	0.040622129539	0.623995063536
N	0.135530534998	-2.018839009269	0.078497843182
N	-0.475011483449	-0.550806900422	-2.074731744998
C	2.503299310586	-1.204352458962	-0.196564344691
C	2.783661508622	1.113702797125	-0.147960457014
H	2.301972968137	2.083014649568	-0.119274147786
C	-0.743503249399	-1.868407628029	-2.207525395932
C	-2.475812771659	0.031104185927	1.924217129318
C	-0.161592465097	-2.535774660472	1.452720163666
H	0.084445367862	-3.604175196164	1.498995736717
H	-1.237095408880	-2.442552243306	1.611895854537
C	-3.084925517336	0.046624956193	-0.324122461826
H	-2.749393243120	0.089019095096	-1.351797875025
C	-0.815000287013	-2.629469935239	-0.906771817167
H	-1.824394399002	-2.563682374860	-0.490338682164
H	-0.587768587385	-3.691804128906	-1.048189417293
C	0.600531790657	-1.789818055219	2.536653028970
H	1.683931778383	-1.906021399959	2.445570675227
H	0.315606635817	-2.170079609044	3.521192383674
C	-1.437616916658	0.146440195326	3.019424666864
H	-1.620274395544	-0.589663092474	3.807013389600
H	-1.522544831226	1.139280447727	3.469096237764
C	4.165537216011	0.984916848701	-0.131130929984
H	4.785516172519	1.873643915020	-0.105382197124
C	3.882078321382	-1.403485329961	-0.164908691368
H	4.281850779402	-2.411579368258	-0.169062051154

C	1.545513734200	-2.355029292510	-0.337683494016
H	1.526599517067	-2.645278639830	-1.392460227752
H	1.893312648132	-3.230505736150	0.218686884426
C	-4.435385084351	0.001504224563	-0.016956571063
H	-5.167065569018	0.002681027291	-0.816528804965
C	-0.402393899637	0.233843465400	-3.165306317860
H	-0.177464528580	1.280769819047	-2.994169623663
C	-3.821767299736	-0.026499469956	2.300403568114
H	-4.075428129464	-0.045762716238	3.355000393292
C	4.724399191808	-0.294239981424	-0.134804319234
H	5.801029149015	-0.427068892658	-0.111127129008
C	-0.959383518019	-2.444310639121	-3.455017654551
H	-1.166809032611	-3.505526372505	-3.536679404092
C	-0.896227007079	-1.630527351606	-4.588351375205
H	-1.059943208020	-2.056811181810	-5.572758025482
C	-4.812516712861	-0.045685801412	1.326157361098
H	-5.859342278108	-0.087936315755	1.608490292769
C	-0.609769802709	-0.272475934999	-4.443444888808
H	-0.540973279488	0.391624887486	-5.297389411175
N	-0.237963044259	1.809908482556	-0.325639336743
O	-1.095270098153	2.939998969700	1.831139121192
S	-0.406043482708	3.216230523260	0.558873550736
O	0.931483224797	3.835467353220	0.598063309559
C	-1.474889380567	4.176512549196	-0.484979632741
C	-0.916462835494	4.941871088574	-1.514966564661
C	-2.857713611685	4.130381634158	-0.277413902533
H	-3.272792990759	3.535501816120	0.528589428857
H	0.158135030393	4.971130714340	-1.658370992337
C	-1.764326397227	5.673526601956	-2.341221306873
H	-1.338519086481	6.271396492900	-3.141932911140
C	-3.156952206792	5.651880115652	-2.157294089898

C	-3.685803665840	4.871341497537	-1.116189467768
C	-4.062560704168	6.471222063081	-3.042086291652
H	-4.760450324059	4.845395993723	-0.959191966674
H	-5.051197789025	6.011880754891	-3.137117824860
H	-4.206764974105	7.474744121518	-2.620644272255
H	-3.637589649359	6.595718637066	-4.042751916720
S	0.611140166709	3.578265228100	-5.229745633988
C	2.343830655075	3.788616065338	-4.686714821305
H	2.328813609357	3.936196584019	-3.603684269010
H	2.935835064284	2.898361508941	-4.918061074558
H	2.796321825629	4.665185737253	-5.159179029472
C	0.868600127428	3.357118861251	-7.026046962693
H	-0.116033086462	3.218828964175	-7.479897926140
H	1.341416893435	4.241809512603	-7.462161792205
H	1.482254096238	2.474238015642	-7.227034847769

$^{3}TS_{2c}$

Fe	-0.703906817437	0.203689167970	-0.539707659182
S	-1.436398004597	0.119525586130	2.063983150229
N	1.185066639745	-0.568789826318	-0.416350369099
N	-2.698968015553	0.632886550721	-0.737852727568
N	-1.203116087221	-1.940398152332	-0.255159392212
N	-0.466417826423	-0.648120127711	-2.660969359714
C	1.292150474050	-1.889104148491	-0.154322469810
C	2.292375294017	0.113297581801	-0.765955611985
H	2.137388524866	1.149895293310	-1.022887706562
C	-0.760894616306	-1.960245532739	-2.724184355334
C	-3.502533696114	0.940239544902	0.311862362913
C	-2.329302422527	-2.194344646692	0.700827688201
H	-2.472922553497	-3.277681479600	0.811722844887
H	-3.235198785878	-1.791916589928	0.247413180771
C	-3.249570301587	0.559359508133	-1.973275489838

H	-2.571080916990	0.324681048684	-2.780870117730
C	-1.601421860681	-2.491760308934	-1.589235133051
H	-2.641242473470	-2.195295864752	-1.761724712141
H	-1.578989069221	-3.586826433976	-1.566216861938
C	-2.119591040738	-1.582692230856	2.086878739111
H	-1.420945772312	-2.160946609258	2.695814978080
H	-3.071584565958	-1.577863921935	2.624803243714
C	-2.922954383240	1.109742875374	1.693286039515
H	-3.686430261746	0.910273584060	2.448857700530
H	-2.582357997443	2.143116573283	1.815632684363
C	3.548162615930	-0.475382800659	-0.823101581100
H	4.406839850063	0.123217379243	-1.105105392443
C	2.519753592792	-2.548982446191	-0.199947898885
H	2.563685134167	-3.610848991335	0.016475080898
C	0.038068038960	-2.608970364653	0.247604892186
H	0.064917087999	-3.654100454983	-0.079431700432
H	0.004590461654	-2.628602827338	1.338907515708
C	-4.599450046876	0.766143892712	-2.215704924106
H	-4.978195837312	0.686249085658	-3.228049170394
C	0.225951123610	-0.087568935910	-3.665499553918
H	0.461211725759	0.965868309429	-3.558169561808
C	-4.871955201671	1.154308597583	0.131142194813
H	-5.485708219548	1.388557938151	0.993892151894
C	3.667472611737	-1.832800861706	-0.527134337091
H	4.632155927953	-2.328141664041	-0.565844797143
C	-0.376396939364	-2.753916701344	-3.803544022587
H	-0.624837098548	-3.809781560007	-3.819586317009
C	0.326856891498	-2.164002133004	-4.854557993693
H	0.636275018619	-2.759075257882	-5.707846145752
C	-5.432050006445	1.067922272439	-1.139293631182
H	-6.495140319157	1.230347840340	-1.283869034623

C	0.632922942662	-0.805008183680	-4.787194578613
H	1.182804219828	-0.307852171407	-5.578504831020
N	-0.084635805922	1.927280628800	-0.689229020190
O	-0.888897578758	3.440407510335	1.291874266391
S	0.311121597395	2.883718557221	0.633008504874
O	1.230181605520	2.059530458524	1.444666496728
C	1.247877995341	4.281104200916	0.012806935059
C	2.478103311098	4.084978544245	-0.625967359112
C	0.778072750970	5.570555090432	0.270988322243
H	-0.154306128721	5.712201022202	0.805338810177
H	2.868522978030	3.088338356789	-0.794507777700
C	3.213998319435	5.190471287909	-1.039148536565
H	4.165861664779	5.035884820020	-1.539872375777
C	2.753255595691	6.499812792580	-0.813714096640
C	1.532209972912	6.668610822844	-0.148301943127
C	3.565983351010	7.685556452231	-1.271395764124
H	1.164432068860	7.670664693661	0.053273120634
H	3.082324425204	8.629318255315	-1.004882777680
H	4.565084358249	7.674069822897	-0.819915609225
H	3.705200025866	7.669043501807	-2.358968548089
S	-1.346237211812	3.243563133321	-2.197988581826
C	-2.364496143048	4.570043232067	-1.472010550885
H	-3.057130440517	4.105385380643	-0.769742384626
H	-1.740665409549	5.301778512657	-0.957975863882
H	-2.920886459972	5.048399991242	-2.283535117356
C	-0.101517283466	4.206934494421	-3.112171954073
H	0.690160876119	3.519295934056	-3.413468657821
H	-0.586284279074	4.620038129281	-4.001224369738
H	0.308686806309	5.006776971320	-2.496015177523

$^{5}TS_{2c}$

Fe	0.213022980161	0.428159251233	-0.657292307968
S	0.654963785598	0.394739402569	1.909652477637
N	2.302262198171	-0.130701095248	-0.598451198847
N	-1.834161954246	0.729378805425	0.139126451998
N	0.079406899820	-1.767513358955	-0.376729024967
N	-0.557494958824	-0.278143696714	-2.554744362886
C	2.550080885127	-1.455173901500	-0.645593952478
C	3.321170210998	0.723376189542	-0.377662428200
H	3.055279558315	1.770408743443	-0.304714252732
C	-1.011360264952	-1.550885691724	-2.572446239325
C	-2.129877615200	0.721937733480	1.455481864818
C	-0.174753119748	-2.157494836952	1.042399504956
H	-0.048319006071	-3.242754384573	1.155984920013
H	-1.220193058286	-1.928582226255	1.259519783001
C	-2.848973461464	0.821749293180	-0.749485138327
H	-2.569660296001	0.863842187170	-1.793219130690
C	-1.036615357796	-2.251158469532	-1.237019471243
H	-1.979159578464	-2.017757133042	-0.732498879844
H	-0.994431397506	-3.339895131848	-1.359820650972
C	0.723248221363	-1.440129226692	2.041755671890
H	1.777772275165	-1.695908433126	1.914891227498
H	0.441793707136	-1.726301762034	3.058895470988
C	-1.030178485219	0.760821508599	2.495219261108
H	-1.265126025024	0.098340372995	3.332687572156
H	-0.965904405303	1.778782440714	2.889169426897
C	4.633933699883	0.290539087910	-0.238986721037
H	5.424332404758	1.012621057062	-0.068895884402
C	3.841041967037	-1.964583885638	-0.507430293578
H	4.006468159231	-3.035897267836	-0.545961459797
C	1.361365228502	-2.344612217787	-0.896440277133

H	1.255628126295	-2.471727535978	-1.978668244129
H	1.522845719600	-3.342691548150	-0.473986628601
C	-4.183687322228	0.864335134740	-0.374881659530
H	-4.953072890103	0.933437540795	-1.135267792895
C	-0.590348770690	0.450016319480	-3.685497854375
H	-0.242733587036	1.471031695432	-3.593201574099
C	-3.455137554762	0.749393594941	1.903811410951
H	-3.654955855904	0.728511351864	2.970002414588
C	4.898733496176	-1.078275033442	-0.312342504158
H	5.911793252566	-1.451843236158	-0.203782056882
C	-1.486252028553	-2.139953657682	-3.741605504521
H	-1.832597181854	-3.167685460207	-3.728307803650
C	-1.512723282559	-1.386067067814	-4.915113216789
H	-1.881102725385	-1.824100042588	-5.837100522350
C	-4.495344964214	0.813678038589	0.983863373182
H	-5.526805055011	0.833766164467	1.320646654595
C	-1.063392795346	-0.065250339805	-4.886973441928
H	-1.075949140320	0.556865318946	-5.774598970126
N	0.492842924827	2.154644069823	-1.107089018708
O	0.009886996691	3.275734939831	1.159639573313
S	0.099976312016	3.554848309014	-0.289284878082
O	1.054139380803	4.598203255462	-0.719919546928
C	-1.534042465093	4.057639459453	-0.829956121659
C	-1.790366699247	4.266050028758	-2.190156320799
C	-2.515519051542	4.310597927028	0.128595893382
H	-2.303226233778	4.151341298138	1.179459641529
H	-1.024330827099	4.080751387410	-2.934913729551
C	-3.045247920376	4.718932862533	-2.583464969278
H	-3.245060212408	4.878131748361	-3.639841476683
C	-4.054263800558	4.979890589706	-1.639391842523
C	-3.767523654134	4.769453828410	-0.284638555965

C	-5.405942429599	5.482641669520	-2.084219623074
H	-4.531517474319	4.967236234595	0.462131502273
H	-6.097105094362	5.571439907645	-1.241380368222
H	-5.320719867770	6.468640632346	-2.557241242883
H	-5.853753835547	4.809802134247	-2.824883678123
S	2.401445999003	2.969810776614	-2.750560339445
C	1.805522138885	4.322739222801	-3.812160078351
H	1.479166957456	5.122544489260	-3.147430077996
H	2.635973023946	4.669496554681	-4.434532130142
H	0.983072736747	3.991076040900	-4.450321492445
C	2.756917826692	1.700708510737	-4.008789520882
H	3.018564400842	0.778775353117	-3.486610604086
H	1.897646958650	1.529095027992	-4.660023913457
H	3.610427271439	2.032964150322	-4.607591119030

$^{3}PC_{2c}$

Fe	-0.724009160411	-0.060846808388	-0.573713065643
S	-1.187200635304	0.058868675908	1.726499509075
N	1.146837427520	-0.819645661263	-0.154959483936
N	-2.769648508905	0.477298101397	-0.809718895799
N	-1.204102066237	-2.288618633009	-0.400206260577
N	-0.465974615657	-0.643442837234	-2.487518596078
C	1.288176827354	-2.154546841532	-0.052007420712
C	2.235988531923	-0.030028375795	-0.043385852937
H	2.052222975753	1.034119632419	-0.098765010655
C	-0.964398483107	-1.864251100696	-2.807450706979
C	-3.491221987388	0.802439050260	0.292090793895
C	-2.175522139980	-2.432233742924	0.708109880751
H	-2.340964682065	-3.489862447286	0.961177057060
H	-3.132197281369	-2.027402451891	0.372412765860
C	-3.408092681207	0.430873942874	-2.001100799079
H	-2.799260595357	0.181328456647	-2.860290206403

C	-1.768481786920	-2.573263944691	-1.735689291231
H	-2.799481729914	-2.205250749773	-1.754684113593
H	-1.811251737207	-3.649449680329	-1.946917710957
C	-1.708969924426	-1.702508076311	1.970464655799
H	-0.822161426483	-2.174954787404	2.402889764209
H	-2.490152769881	-1.723539260124	2.735357775127
C	-2.777288971503	0.955832471686	1.612340761703
H	-3.419333454987	0.667161521651	2.448127744440
H	-2.502095317786	2.008197275118	1.755413743787
C	3.508826007542	-0.543479068547	0.162755424557
H	4.354145343335	0.130451814046	0.244796162011
C	2.538767227609	-2.738458322549	0.163019851198
H	2.617377161840	-3.817864468147	0.245629173118
C	0.055498517905	-3.032233277950	-0.164400422971
H	0.215096951796	-3.756534720652	-0.971165021025
H	-0.033598416103	-3.628134741749	0.750768228074
C	-4.764954880106	0.685260272134	-2.147548231725
H	-5.218852273212	0.632050754577	-3.130407512467
C	0.270784471501	0.021508880174	-3.397742018608
H	0.683605387345	0.969384432780	-3.074241480901
C	-4.863173012446	1.057961021473	0.219698689870
H	-5.408757727363	1.303836372949	1.124226984560
C	3.663697360895	-1.927140631815	0.267039092642
H	4.643114100283	-2.365365280420	0.430504443687
C	-0.757944440835	-2.422923159110	-4.066991876452
H	-1.164778236898	-3.403249169671	-4.290595949526
C	-0.022213096290	-1.717007716162	-5.018240896144
H	0.146666686874	-2.138626045668	-6.003783066133
C	-5.511067159724	0.997851195744	-1.011197532684
H	-6.576401689575	1.192339476624	-1.080843418861
C	0.508933898918	-0.474583776440	-4.674557952726

H	1.104678835511	0.101754728873	-5.373193579006
N	-0.292248655655	2.444245689768	-0.918784976677
O	-1.058010611258	4.081829789731	0.963241638738
S	0.127790992193	3.404145472747	0.395077521864
O	0.882206125482	2.487613482930	1.270007650282
C	1.259720124270	4.680940715315	-0.159234575791
C	2.478959244034	4.318991064152	-0.743409949237
C	0.930081091068	6.022988053715	0.040460551565
H	-0.011162490151	6.287402599593	0.509399827300
H	2.737914772527	3.275254045768	-0.888208882583
C	3.360794405030	5.317819347906	-1.144314154426
H	4.307825653716	5.038900696389	-1.598328021925
C	3.050441189549	6.677418939516	-0.968467102769
C	1.828942791120	7.010180427396	-0.366828141799
C	4.005629899567	7.748833557167	-1.434143243400
H	1.575564955089	8.054987529338	-0.209800767834
H	3.796206455159	8.708416470546	-0.952553967091
H	5.044746672564	7.474717057300	-1.223737718098
H	3.923419810559	7.897036011872	-2.518728614860
S	-1.176916008375	3.067835866505	-2.184870391602
C	-2.600278725866	4.083549902928	-1.635379616454
H	-3.247969065417	3.442515216450	-1.037748285430
H	-2.280321087093	4.946490391680	-1.054015912096
H	-3.112885297252	4.385016890186	-2.552273489099
C	-0.222693614230	4.329787440844	-3.104340074275
H	0.728682986698	3.873423530327	-3.381559408795
H	-0.807941992370	4.542771969088	-4.002645928907
H	-0.064255446215	5.235879541038	-2.520895094215

$^{5}PC_{2c}$

Fe	0.293862090235	0.509479354982	-0.856292881901
S	0.624185231856	0.430168354919	1.788979710998

N	2.419902600341	-0.156446832633	-0.573925625448
N	-1.855365777618	0.820496151886	-0.006078980605
N	0.123632816134	-1.795105990220	-0.489754804903
N	-0.602340291028	-0.287708332319	-2.730178836117
C	2.612903726259	-1.491212052130	-0.575275781045
C	3.452228919521	0.649263259695	-0.252680684899
H	3.237570116643	1.711793507771	-0.213651508043
C	-0.945515508983	-1.593688177097	-2.722267227259
C	-2.151487864376	0.815915591500	1.310198815151
C	-0.202603179304	-2.144513102561	0.918465595278
H	-0.078956513119	-3.225903664044	1.079590664629
H	-1.259331664438	-1.914408904989	1.070604342359
C	-2.881871647157	0.857557722039	-0.882727136360
H	-2.613284579245	0.880748392761	-1.930524172555
C	-0.948816218852	-2.290458661761	-1.382286248913
H	-1.909489160679	-2.079847322715	-0.900501720930
H	-0.890719982281	-3.378360421196	-1.517777097558
C	0.624033257144	-1.400060368287	1.964249397401
H	1.679484486187	-1.681286308110	1.932220823729
H	0.255979213216	-1.654715312403	2.962398673326
C	-1.044076967232	0.913371116393	2.336525915180
H	-1.302528951967	0.354153290970	3.239653141567
H	-0.927968117335	1.963209064745	2.624996939905
C	4.723049262534	0.167175319591	0.034249338989
H	5.521493402721	0.857631358063	0.281129449083
C	3.859314232899	-2.051956555290	-0.290076514812
H	3.979298237166	-3.130185105619	-0.295433126350
C	1.426713573349	-2.356262887974	-0.935042341052
H	1.394457641768	-2.441604572842	-2.027118015950
H	1.566905844592	-3.371724934540	-0.542277021085
C	-4.218034364191	0.863774985725	-0.503509190602

H	-4.993526171751	0.892457491978	-1.260811720142
C	-0.733670010980	0.408694670135	-3.875428650788
H	-0.492961761831	1.462876013789	-3.814289154975
C	-3.473259143006	0.799658045570	1.770023742412
H	-3.665930156096	0.779030819179	2.837694960652
C	4.931089673552	-1.212876812214	0.006861247162
H	5.908060418086	-1.630037546927	0.229081222403
C	-1.377158744295	-2.244584539923	-3.877978861848
H	-1.627707955359	-3.299373808494	-3.838141018422
C	-1.491490556766	-1.523440259998	-5.065097517807
H	-1.827676015943	-2.012467810437	-5.973752085678
C	-4.521897927873	0.820774130872	0.856728147504
H	-5.551807279233	0.808542151138	1.199399743687
C	-1.176617033138	-0.164365567632	-5.061918643365
H	-1.267447279762	0.442664143837	-5.955520956303
N	0.758421545799	2.627626035991	-1.685535085942
O	0.375992976133	3.298958185497	0.801098582037
S	0.321571178885	3.827210025509	-0.575140231793
O	1.135078558344	5.034514305642	-0.861563403268
C	-1.396842399266	4.180718950712	-0.934939223157
C	-1.858304236736	4.283442634770	-2.249860795721
C	-2.237943104934	4.472704510674	0.142468905421
H	-1.869874154217	4.395576925139	1.159146047362
H	-1.207579441329	4.058250249040	-3.086116836568
C	-3.176327813692	4.670306629301	-2.479762912766
H	-3.534878268310	4.746482299547	-3.502694331790
C	-4.045111651368	4.964211475902	-1.416754283724
C	-3.552817047360	4.861791478421	-0.107631570999
C	-5.478515682203	5.356710077958	-1.678334657235
H	-4.205589075312	5.089729221580	0.730428991401
H	-5.886832548129	5.951068458352	-0.855339128981

H	-5.571400834714	5.934454906979	-2.603485657401
H	-6.111037493789	4.465826358222	-1.785935024036
S	2.173587829041	3.120158634096	-2.521235969199
C	1.663283672932	4.281502503667	-3.833126794087
H	1.355426461901	5.204043080686	-3.343102674440
H	2.549496755882	4.459011632926	-4.448606908581
H	0.858911923641	3.850371278521	-4.431513118808
C	2.541403007851	1.683521164821	-3.566658399713
H	2.734142747325	0.824126107630	-2.927483092522
H	1.726425218658	1.483669348310	-4.260626961583
H	3.450487954604	1.951011434927	-4.110615809606

$^{3}RC_{2c,H1}$

Fe	0.061082264073	-0.031127655843	0.301200697980
S	0.433841854743	-0.194244056644	2.600649856675
N	2.041755226637	-0.078011965338	0.022374455605
N	-1.940723890572	-0.111099835117	0.778940675625
N	0.260933632561	-2.130439709414	0.243955655766
N	-0.247752759170	-0.378967393889	-1.651297384863
C	2.596470293704	-1.296065054794	-0.139646253156
C	2.837453424784	1.013893492293	0.038192152162
H	2.347432516982	1.966355303031	0.189575718267
C	-0.582456613671	-1.650910214014	-1.975957902009
C	-2.328847467191	-0.169872538467	2.074790378762
C	-0.037222871901	-2.738458335495	1.575372863459
H	0.221475177592	-3.804427637406	1.571419965806
H	-1.112314443344	-2.662767136222	1.746861461401
C	-2.898472146194	-0.033284382355	-0.174101580128
H	-2.557327506792	0.062621194511	-1.194624057438
C	-0.706375673527	-2.584467366402	-0.800365467830
H	-1.715023766945	-2.533031496309	-0.381756814847
H	-0.519565767114	-3.624792534566	-1.085212398841

C	0.733792173352	-2.015098954613	2.666753865201
H	1.816574486492	-2.130611076607	2.569887891045
H	0.451929706825	-2.372695522293	3.660106173872
C	-1.299897056657	-0.084179635293	3.172008208212
H	-1.460239346413	-0.840977184854	3.944021211706
H	-1.374100653554	0.899077984348	3.644100193755
C	4.213526372462	0.921657989496	-0.116487796535
H	4.812915449043	1.824626929591	-0.098403463684
C	3.972817021848	-1.460232474171	-0.289444736209
H	4.383919827207	-2.456348618526	-0.412891221179
C	1.659211929016	-2.469372913340	-0.203016020423
H	1.618967344666	-2.824519516191	-1.236893615833
H	2.043003654381	-3.304646311680	0.389639399150
C	-4.254153801218	-0.068549420934	0.113292245265
H	-4.972334704250	-0.007276961371	-0.696106673620
C	-0.117440786665	0.547706878550	-2.618418084902
H	0.153562920172	1.543784299042	-2.290782657162
C	-3.678371220323	-0.229266382250	2.431467086367
H	-3.946198971456	-0.293144627905	3.480624168738
C	4.793715281759	-0.336696794724	-0.281897254803
H	5.867320056796	-0.441451320306	-0.398737535833
C	-0.805657195786	-2.026248834613	-3.296070777744
H	-1.064264245693	-3.053683401921	-3.527593834912
C	-0.688651323718	-1.064039109028	-4.300147068358
H	-0.863385496407	-1.333364377811	-5.336601001895
C	-4.655413596258	-0.185651413281	1.444086995574
H	-5.706955489276	-0.228344887262	1.707903110550
C	-0.336505244676	0.241462960128	-3.957150093080
H	-0.237201373596	1.021285940670	-4.705011960326
N	-0.057036912060	1.707894847151	0.218940409398
O	-1.134445047743	2.844199982672	2.248413983471

S	-0.272495781746	3.107431257441	1.081290186355
O	1.072023365558	3.668294144035	1.311394516000
C	-1.152620144294	4.142071813414	-0.065596721629
C	-0.428462804000	4.933006775293	-0.965860621383
C	-2.550404897355	4.139848143687	-0.060061196210
H	-3.097425206490	3.530974463517	0.651352968054
H	0.655875763387	4.932978569418	-0.947370748931
C	-1.124766287312	5.731686895448	-1.867490113307
H	-0.569831599283	6.356894481581	-2.561708337173
C	-2.530812852852	5.751102815556	-1.888473174516
C	-3.226680485491	4.947123288369	-0.973041963105
C	-3.266364447775	6.627108036756	-2.872028375301
H	-4.312836652261	4.956352723236	-0.969924076982
H	-4.349825299378	6.514664492833	-2.777137332783
H	-3.018426599666	7.683885611195	-2.716049778080
H	-2.985336918469	6.380898134465	-3.902918606048
C	-2.147315807298	2.047634755871	-7.931378858682
C	-3.031536538628	2.259870742425	-6.952236401211
C	-2.713339292131	3.024231017694	-5.692659190534
C	-1.283754268798	3.500213567564	-5.641978282226
C	-0.398262187230	3.286515415542	-6.621580428563
C	-0.726208849263	2.549142714255	-7.894939871501
H	-2.445085206919	1.487523574481	-8.816646017862
H	-4.044626814006	1.871074283552	-7.044903549673
H	-3.397331376882	3.883537677505	-5.591351665226
H	-0.983047564087	4.049550861129	-4.750484275187
H	0.618441623492	3.664402797886	-6.522846939084
H	-0.026490825793	1.708503149266	-8.032885609873
H	-2.931705268333	2.400790919260	-4.809632270968
H	-0.536387749621	3.202202187092	-8.763261432574

$^{5}RC_{2c,H1}$

Fe	-0.105260476727	0.067866085156	-0.076150043894
S	0.644017099021	0.071897333142	2.410380201875
N	1.921368425626	0.026688201315	-0.491961779543
N	-2.013656129165	-0.008925865417	0.878030380111
N	0.180002310197	-2.044507927115	0.085330332184
N	-0.779351287036	-0.659938323869	-1.989606404669
C	2.457415597444	-1.211352752887	-0.584428994901
C	2.722684692637	1.109040686874	-0.594720834084
H	2.244837239147	2.074933629295	-0.493350934773
C	-1.073617745229	-1.978482782332	-2.019381304550
C	-2.189951721801	-0.034888595987	2.214155599114
C	0.145111322118	-2.532873069444	1.500129632987
H	0.443952962023	-3.588171042087	1.525412961190
H	-0.890276223610	-2.481136522154	1.842107112131
C	-3.099583960164	-0.005448186723	0.069906002384
H	-2.907237798728	0.054629263957	-0.992916741612
C	-0.920090386432	-2.690644360140	-0.698903422858
H	-1.843240757548	-2.609630909642	-0.117779357376
H	-0.713038652829	-3.757039478320	-0.839973865183
C	1.046047353838	-1.723869652665	2.418917150802
H	2.100245010364	-1.781481920591	2.135338563475
H	0.962812774991	-2.092926885294	3.444476174859
C	-1.016886643925	0.092855768390	3.162294209112
H	-1.058875360936	-0.675802690622	3.938829891326
H	-1.082870845673	1.065254786314	3.657052747853
C	4.090794209776	0.989794825722	-0.797680875914
H	4.699326139374	1.883241497691	-0.876728338887
C	3.824724824961	-1.400269813908	-0.772119887098
H	4.226074707636	-2.405749241653	-0.834209491582
C	1.498776318315	-2.368960892718	-0.569006882423

H	1.300726589819	-2.650076376938	-1.607777033361
H	1.944293594816	-3.245836076155	-0.089416593953
C	-4.394652869203	-0.074588455499	0.556800454716
H	-5.228100014724	-0.074153089938	-0.135954602010
C	-0.886471113755	0.077301517051	-3.110910561942
H	-0.627712189716	1.127426416952	-3.027101888390
C	-3.471440326605	-0.119705733766	2.768755149567
H	-3.578623979521	-0.152513484238	3.847793598439
C	4.652302011651	-0.284974892884	-0.883259651883
H	5.719896618638	-0.410019051715	-1.031279635836
C	-1.501565228704	-2.603357504991	-3.185702972594
H	-1.723628085189	-3.664889400237	-3.184215573906
C	-1.627376104954	-1.838606166620	-4.346812071433
H	-1.958615708175	-2.303296874212	-5.269858746110
C	-4.585070265060	-0.144478918440	1.937908248854
H	-5.582829014150	-0.206467653751	2.359574129575
C	-1.311010672698	-0.479681246539	-4.312846553851
H	-1.385761349703	0.141627251227	-5.199151678416
N	-0.314057096464	1.781490995734	-0.338463329887
O	-0.964714700823	2.866593001632	1.905534992248
S	-0.324557888728	3.157945377435	0.610574439570
O	1.052386639726	3.685685204946	0.607330224501
C	-1.371862940322	4.250152962229	-0.320183944725
C	-0.789675855651	5.191797472024	-1.176714048243
C	-2.760323007196	4.158521175864	-0.169197507093
H	-3.196126139491	3.438619560208	0.515139100626
H	0.288836532324	5.258918150743	-1.265641587928
C	-1.620125652933	6.054652807144	-1.888388114282
H	-1.176682912112	6.800361737781	-2.542379564342
C	-3.017224863391	5.979397521596	-1.766307268866
C	-3.570106799101	5.025840190598	-0.896009373800

C	-3.899490674355	6.898755702537	-2.572568425140
H	-4.648427110504	4.969429491382	-0.777253566596
H	-4.880692124337	7.024637698828	-2.105296546596
H	-3.441361530065	7.885406779990	-2.694918994627
H	-4.062427214689	6.489258109358	-3.578264424097
C	-0.564634359049	1.945961337975	-7.396610046186
C	-1.791350306913	2.342636565382	-7.040055006148
C	-2.074503741660	3.182667212438	-5.821907105564
C	-0.830750000159	3.507152364493	-5.033271134143
C	0.396090816950	3.111821780109	-5.390615107681
C	0.682497295515	2.296532621908	-6.625844650124
H	-0.431760939011	1.343076131323	-8.293731877578
H	-2.649054738326	2.059563476219	-7.648746974486
H	-2.581563807547	4.115164946036	-6.123101689005
H	-0.965580783589	4.104292945610	-4.132953714961
H	1.253108418837	3.390801973456	-4.778906654244
H	1.225673268635	1.376915269533	-6.352474394355
H	-2.807061287195	2.672945831738	-5.174553379426
H	1.385232881195	2.840908240156	-7.278651144344

^{3}TS1$_{2c,H1}$

Fe	-0.276604760985	0.012465355324	-0.448959842283
S	-0.395983616740	0.104683764716	1.903986046162
N	1.686262905617	0.423019629785	-0.260812371873
N	-2.237047850112	-0.646447787180	-0.345676305563
N	0.405691525187	-1.982627476710	-0.189830745887
N	0.019956603094	-0.527630911582	-2.369989771599
C	2.515756077480	-0.630538198169	-0.115613121785
C	2.204295125206	1.668176376587	-0.227773817367
H	1.496226137549	2.475088832253	-0.310678805445
C	0.131047649105	-1.860507154800	-2.595887328380
C	-2.887298241500	-0.622124904098	0.845112767913

C	-0.128590244993	-2.567631206725	1.078017313160
H	0.336143663770	-3.543563363459	1.265374621473
H	-1.198348602939	-2.732688225072	0.948326492717
C	-2.900525888451	-1.117284752774	-1.424330695279
H	-2.372102319799	-1.086535875928	-2.365978348452
C	-0.091935079103	-2.727496856685	-1.384458802718
H	-1.155895987098	-2.932346105207	-1.246261835033
H	0.413224862533	-3.693851780927	-1.483894552596
C	0.131826666874	-1.631491126057	2.247722242630
H	1.196189831256	-1.551421640089	2.485808992071
H	-0.379365713156	-1.976463283338	3.150285002642
C	-2.213118125406	-0.008535919731	2.044078149820
H	-2.465815251415	-0.539603833937	2.964974088124
H	-2.547282556028	1.029547043587	2.146511262725
C	3.561337799677	1.904898332121	-0.062383175226
H	3.925570401219	2.925647359443	-0.044415995975
C	3.888611312352	-0.466367712659	0.066050839770
H	4.520324666196	-1.340266629513	0.184843909332
C	1.909533338491	-2.002185850236	-0.194940091067
H	2.254517424172	-2.481987187633	-1.115493130423
H	2.272353119249	-2.628677997007	0.625385859395
C	-4.194787937296	-1.615393634869	-1.362661686541
H	-4.666170910360	-1.986353456502	-2.265477606136
C	0.258647504147	0.334241402622	-3.373852372186
H	0.187022692377	1.384687529733	-3.125341109738
C	-4.189429899938	-1.108464054219	0.976367676104
H	-4.669182002277	-1.082425374754	1.948612680560
C	4.422618775850	0.817880576238	0.088354045075
H	5.488432360295	0.967398974529	0.226022619660
C	0.445260364427	-2.362327186856	-3.854059336248
H	0.530279010017	-3.433420791561	-4.000655188570

C	0.665741159705	-1.468853340914	-4.903301265176
H	0.918137020080	-1.838061686721	-5.891890493402
C	-4.852983223573	-1.619454179202	-0.134602452546
H	-5.862083364740	-2.007486749265	-0.043213645338
C	0.581350908761	-0.099867708110	-4.655964583297
H	0.765760833771	0.629520986596	-5.436111343963
N	-0.871532413771	1.737285011376	-0.871244837148
O	-2.667616606887	2.858742473954	0.607535144463
S	-1.253436189229	2.959843009690	0.174011730601
O	-0.237763658376	3.006965395426	1.254811989692
C	-1.100327083326	4.486880441458	-0.760453652183
C	0.047581312068	4.762646354145	-1.511168868393
C	-2.112898360895	5.440713602175	-0.633008866110
H	-2.999148316855	5.215558226017	-0.050406245468
H	0.829704963238	4.020843499220	-1.628372752384
C	0.170012113992	5.999638451388	-2.139646981285
H	1.058260263714	6.208355897434	-2.729830630530
C	-0.832383999547	6.977434522188	-2.025580954426
C	-1.971864409571	6.675045537341	-1.267713793152
C	-0.672547858814	8.324212495113	-2.687419082589
H	-2.763388767734	7.413147712842	-1.171250122225
H	-1.636709312566	8.826187684418	-2.810554958584
H	-0.032978484197	8.979934755558	-2.082361204424
H	-0.201539767529	8.232068008798	-3.671608151949
C	-3.789882801856	0.897894776471	-4.433487912534
C	-3.700170431793	1.450231373456	-3.204384008410
C	-2.693997705760	2.469359840357	-2.871154912103
C	-1.915356419037	2.990953736700	-4.009064138648
C	-1.993842334635	2.450250336040	-5.242903766896
C	-2.903548580088	1.306166705536	-5.569081132552
H	-4.551539069762	0.149771354117	-4.640278214594

H	-4.393442708284	1.153395865311	-2.422918506933
H	-3.077263028986	3.249488653074	-2.203956869121
H	-1.259208650429	3.837529954761	-3.827275376216
H	-1.402745903657	2.863308668663	-6.056908293547
H	-2.309963204416	0.439883068240	-5.913246016876
H	-1.870738248706	1.987250095529	-2.037677816176
H	-3.518965498856	1.557857272164	-6.449171588536

^{5}TS1$_{2c,H1}$

Fe	-0.197731504472	0.278118176493	-0.581990202197
S	0.711764660342	0.516962165361	1.841541964686
N	1.960371389387	0.132619082793	-0.934456137431
N	-2.066782630629	0.093300706896	0.646756883409
N	0.307983324189	-1.969094585030	-0.140392710871
N	-1.062734685787	-0.848672800714	-2.221273629519
C	2.515214701005	-1.094887908446	-0.948840124075
C	2.754283397476	1.220095436390	-1.024438565606
H	2.256504890735	2.182136827109	-0.970315909096
C	-1.203117035219	-2.184864916136	-2.071049410182
C	-2.108282803496	0.216489880450	1.988604650163
C	0.417200599771	-2.243631127354	1.316357473421
H	0.835352775722	-3.246352407671	1.484701859448
H	-0.594692551794	-2.242445796006	1.727593238361
C	-3.226725996674	-0.112235971771	-0.018563325407
H	-3.155774541294	-0.183875559573	-1.095863565560
C	-0.800369199702	-2.755333655926	-0.732530150472
H	-1.658438883326	-2.697471429034	-0.055040236750
H	-0.538193091555	-3.816480619946	-0.828218337074
C	1.269094718680	-1.223007574133	2.062675065436
H	2.311542047787	-1.227952604985	1.735697369904
H	1.265071177091	-1.448472556725	3.132566755986
C	-0.857795895096	0.553467553792	2.769994777821

H	-0.759408441577	-0.093896684811	3.645970713440
H	-0.940301675569	1.582713894771	3.129618512777
C	4.133250987157	1.119551488330	-1.158816161922
H	4.735466190340	2.018457804655	-1.226809174749
C	3.893191170350	-1.272018974315	-1.079490731298
H	4.309985924570	-2.273535191620	-1.084990910266
C	1.573049579085	-2.271739533413	-0.868602070471
H	1.313084482010	-2.559564443633	-1.892852679982
H	2.076983316146	-3.135766442416	-0.418925239259
C	-4.454168559802	-0.229090103243	0.616137377225
H	-5.350259112150	-0.394971329902	0.029048221134
C	-1.482421057885	-0.265608005764	-3.362156596824
H	-1.364324432832	0.809484039626	-3.421366995159
C	-3.309379747153	0.094692743011	2.696314297863
H	-3.301769333602	0.187469288466	3.777357375556
C	4.712168996827	-0.150860077699	-1.192860701483
H	5.786619833252	-0.266129526182	-1.292932828607
C	-1.751413508469	-2.977476736525	-3.076735355044
H	-1.837305693630	-4.048711290613	-2.930880217255
C	-2.189156555856	-2.373277511873	-4.254240501363
H	-2.625271120164	-2.971964130991	-5.047289522284
C	-4.495048221603	-0.133035279870	2.008197439621
H	-5.433128417291	-0.228226307606	2.545477597255
C	-2.058485310490	-0.991314347589	-4.397534032297
H	-2.396205128656	-0.481784274444	-5.292687355423
N	-0.446026064864	2.028322002179	-1.168056668131
O	-0.889967519217	3.086014664375	1.087847581559
S	-0.509892731918	3.427451827613	-0.303029646828
O	0.744620223619	4.190309471736	-0.494075248135
C	-1.841393993523	4.387277256099	-1.014870527064
C	-1.568106274015	5.647491251291	-1.547996560745

C	-3.147431068067	3.882208058656	-0.994616949258
H	-3.357995288297	2.908177026308	-0.565063916890
H	-0.553809929420	6.030759061525	-1.543838503210
C	-2.616558262147	6.402770646554	-2.077731513572
H	-2.405937135044	7.385653199078	-2.490139030758
C	-3.931218425425	5.918337607690	-2.081511596904
C	-4.177709155728	4.648570855859	-1.529036713573
C	-5.060758229652	6.730099801989	-2.666956276994
H	-5.192247012304	4.259051455376	-1.514309779425
H	-5.904092871672	6.794876503398	-1.970093422041
H	-4.738731364756	7.746412923297	-2.910982870576
H	-5.439296394143	6.267110435690	-3.586923095283
C	-0.888528376765	2.889336305494	-5.667617291739
C	-0.684266194397	3.448513363344	-4.453119316661
C	0.481105506451	3.105622811180	-3.623503009441
C	1.477477996916	2.217660697962	-4.238623282693
C	1.284301371718	1.651631155041	-5.451813122921
C	0.073168966849	1.924501912650	-6.281384577496
H	-1.760327906895	3.165838461304	-6.254929528592
H	-1.392587278471	4.171376572566	-4.059224195559
H	0.911191036723	3.951288250278	-3.070916248486
H	2.392996810592	2.021018511423	-3.689343253898
H	2.045470669090	1.003193679227	-5.877829330101
H	-0.439598581193	0.976667314433	-6.524581789733
H	0.051957981669	2.532068691997	-2.617642456683
H	0.383015468132	2.294293842203	-7.275005053751

$^{3}INT_{2c}$

Fe	-0.086694419891	-0.029590840873	-0.211982944837
S	-0.156470279397	-0.037561471154	2.131643576678
N	1.892437918128	0.237527889069	-0.067345832196
N	-2.100555336096	-0.469906486166	-0.121805092509

N	0.438929925703	-2.050613410620	-0.112621508497
N	0.083411978995	-0.439776261662	-2.172872830242
C	2.649947465924	-0.878443187275	-0.047323429031
C	2.490734332301	1.443494939442	0.029509863994
H	1.836079596993	2.300389813336	0.040212379062
C	0.049974812748	-1.759979382034	-2.485523790824
C	-2.725970020601	-0.492076078684	1.081416746597
C	-0.061773331154	-2.659774852118	1.158837429047
H	0.340050777586	-3.674068553794	1.270352309573
H	-1.147252618309	-2.743492070134	1.088666773715
C	-2.845224514092	-0.676376687457	-1.234088389241
H	-2.331310011325	-0.611400905776	-2.182478874825
C	-0.192443331221	-2.677052293103	-1.314679274071
H	-1.262851176506	-2.785806095395	-1.124485915359
H	0.212652059108	-3.677884877270	-1.493452019116
C	0.338489061531	-1.803678226046	2.349719749944
H	1.420583584035	-1.775294910526	2.502161895449
H	-0.114010213676	-2.174666052290	3.272542375721
C	-1.969609266389	-0.125718999310	2.332134779938
H	-2.201066906104	-0.802419534728	3.158780041670
H	-2.263226280205	0.886020250052	2.628841213369
C	3.868072443720	1.571966085886	0.143348629009
H	4.304978106335	2.561110987740	0.217724378073
C	4.036642502951	-0.820853083388	0.078660826420
H	4.612106713409	-1.740178562022	0.097011617374
C	1.935459397924	-2.187398892398	-0.217065124975
H	2.183403839757	-2.595502179819	-1.201025272478
H	2.288285610221	-2.920926009984	0.513287221138
C	-4.202202818539	-0.955001612454	-1.195422124245
H	-4.738974140658	-1.116189687360	-2.122835816810
C	0.337203236386	0.465379888111	-3.135677896694

H	0.382342021436	1.500867361490	-2.821058392588
C	-4.088311927086	-0.781437046433	1.189257394349
H	-4.547062561132	-0.804340755988	2.171684537713
C	4.656774186858	0.421619179083	0.170839822034
H	5.735446704631	0.490040111035	0.265991463688
C	0.238512465022	-2.201381703829	-3.789779770119
H	0.212656180008	-3.263241335073	-4.007848545406
C	0.473739525927	-1.261601259016	-4.795062697422
H	0.626105064476	-1.584879600908	-5.819473171127
C	-4.837740355731	-1.023486268036	0.044242607725
H	-5.896035566782	-1.251128121895	0.116739703125
C	0.531528350073	0.090406751740	-4.461176833707
H	0.725632656540	0.852079526416	-5.206814525993
N	-0.566308338685	1.777734555675	-0.533105866316
O	-2.197894508752	2.815067738711	1.212430983084
S	-0.890213273383	3.010035432887	0.550195440024
O	0.292785067443	3.141689011693	1.421558792715
C	-1.023380889583	4.450041873508	-0.502755690072
C	0.110012101828	4.928028187345	-1.170639841366
C	-2.255879177764	5.094015618292	-0.623460269211
H	-3.121654513229	4.715016519360	-0.092223672743
H	1.068066487415	4.429165693069	-1.069131190193
C	-0.005788679169	6.059365395713	-1.972437561175
H	0.871284357780	6.432605401961	-2.494176421296
C	-1.233895736552	6.729354811409	-2.114017518451
C	-2.350492278860	6.228986355050	-1.429945216071
C	-1.334692951086	7.972472471928	-2.963408108000
H	-3.308259361068	6.732743410071	-1.526001958561
H	-2.373542516811	8.198840494805	-3.220193851729
H	-0.927609011989	8.840519672112	-2.428943715454
H	-0.762802370136	7.867795823592	-3.891417935420

C	-3.879393673960	1.025554269179	-5.303532704008
C	-4.101246596685	1.667179820193	-4.116081341943
C	-3.270982097976	2.738256331347	-3.684932824844
C	-2.187554985315	3.157981159630	-4.503366902723
C	-1.922702705557	2.552139977262	-5.701755745393
C	-2.758263461030	1.416968184263	-6.225552158997
H	-4.528533469872	0.211960965187	-5.617995211301
H	-4.934542960315	1.361112803348	-3.487355015010
H	-3.479228469316	3.250532051704	-2.751061526699
H	-1.564259612956	3.984125019494	-4.168129783890
H	-1.095557889042	2.894639606917	-6.319025006302
H	-2.118342310101	0.539488576521	-6.439768858185
H	-1.300790337055	1.818991766821	-1.246766938386
H	-3.163408282050	1.675532512572	-7.223153645179

$^{5}INT_{2c}$

Fe	-0.441236559479	-0.288980584081	-0.376031963092
S	0.943093609523	0.424029825054	1.721324624059
N	1.583863544337	-0.843906543010	-1.198653194674
N	-2.075906994285	0.010333996581	1.178365584328
N	0.005691885208	-2.427719073776	0.529320167439
N	-1.673139422721	-1.741069746984	-1.577108747321
C	2.080225231845	-2.046417001904	-0.857854840414
C	2.390784811565	0.046377718349	-1.804781712703
H	1.946258175491	1.011184865992	-2.027679556075
C	-1.794895406927	-2.986886091938	-1.073814301626
C	-1.836005197723	0.511237009338	2.404964445711
C	0.315400674683	-2.335477555162	1.977959771978
H	0.673755986161	-3.305017348969	2.356244821119
H	-0.617123535126	-2.110816328492	2.501032376194
C	-3.352422783418	-0.262723161576	0.834687687724
H	-3.498290621988	-0.654487781159	-0.165386528740

C	-1.239493782049	-3.204632521814	0.317179978384
H	-1.977636854064	-2.856040691362	1.046724551021
H	-1.084207042479	-4.275921564917	0.502480795756
C	1.354297205951	-1.267941066594	2.317086676094
H	2.326607066254	-1.487821988131	1.866999001519
H	1.500173947380	-1.233239898255	3.400550767714
C	-0.434227010866	0.930404154461	2.807036940747
H	-0.216134684085	0.608370966434	3.829588054944
H	-0.389315883416	2.023651900686	2.793967172856
C	3.718380773957	-0.232404043856	-2.113590198461
H	4.329605095314	0.515617365678	-2.606698089381
C	3.407505935267	-2.396311509033	-1.117095118420
H	3.778580338642	-3.371704718368	-0.819035251782
C	1.117499230997	-3.041809859743	-0.246501293563
H	0.676329345222	-3.611545429888	-1.071685823284
H	1.662564554696	-3.766779213888	0.371408492393
C	-4.431339595349	-0.068159854986	1.686767981480
H	-5.435728927539	-0.305709024059	1.353970520045
C	-2.206205214126	-1.474135811511	-2.782049731417
H	-2.095190317540	-0.454841666080	-3.137105582985
C	-2.869528205156	0.718166581699	3.326974332805
H	-2.637708098979	1.111736167514	4.311483244050
C	4.236308574193	-1.479141035526	-1.760171764123
H	5.270706385317	-1.730558792301	-1.973300551974
C	-2.442848353983	-4.004133919366	-1.774349382898
H	-2.511253538644	-4.997703617314	-1.343592846820
C	-2.994599992828	-3.722679403067	-3.023969513494
H	-3.503534765679	-4.499219731101	-3.586346444013
C	-4.180958507578	0.426392341429	2.967772890566
H	-4.991824436123	0.583660361658	3.672252807787
C	-2.876989480883	-2.431587571442	-3.538054146406

H	-3.290652108611	-2.166410505784	-4.504800737187
N	-0.701515214462	1.329217667954	-1.604127844852
O	-0.573345288376	2.902508437634	0.339359706116
S	-0.549329363659	2.867338589107	-1.143133171988
O	0.611549643454	3.539559857470	-1.791475020207
C	-2.007720953668	3.742916401976	-1.733128146955
C	-1.921142895765	5.116157092815	-1.982290604537
C	-3.226411221057	3.075672312937	-1.875357805053
H	-3.285429381185	2.009468833800	-1.684185799029
H	-0.973118953823	5.631236218008	-1.867158156209
C	-3.059951722499	5.814791528188	-2.382353969600
H	-2.989204367734	6.882588103154	-2.573496773538
C	-4.293804602686	5.164673309007	-2.539297808153
C	-4.356519609690	3.788208253044	-2.276555911779
C	-5.514902321972	5.921486331679	-3.004395209898
H	-5.302612594364	3.264442709806	-2.388689806666
H	-6.433106538699	5.490642297425	-2.592500373363
H	-5.466947668066	6.975487497456	-2.712949155387
H	-5.602184009821	5.888806224945	-4.098486473906
C	0.491022933767	6.071965321141	-6.533358183980
C	0.271106458019	5.488418306431	-5.312715121088
C	1.338400347642	4.836464300321	-4.654828225108
C	2.636846456461	4.757566650312	-5.206295112465
C	2.884086438017	5.332821210048	-6.425753791419
C	1.819050296878	6.028423495298	-7.163809309693
H	-0.307678411686	6.581223687512	-7.063914622840
H	-0.705429230119	5.520211835358	-4.842311417962
H	1.147372341101	4.376939159576	-3.684940012195
H	3.421035117357	4.245340973356	-4.660385418336
H	3.871042091922	5.290809752051	-6.876105014678
H	1.733582297310	5.601130936071	-8.182031206774

H	-0.503826977889	1.252825924270	-2.602121296037
H	2.148173854936	7.059145182414	-7.402142308281

^{3}TS2$_{2c,H1}$

Fe	-0.330365596724	-0.025713987436	-0.379915069490
S	0.460706145145	0.291307789941	2.052990917471
N	1.589373090618	-0.001570454496	-0.971971981699
N	-2.192988644184	-0.159376114323	0.512621308884
N	0.156190963041	-2.157575117469	0.004444926156
N	-1.047804365579	-1.187744062496	-2.267951506937
C	2.262215210337	-1.173298804306	-0.945179253493
C	2.266672716248	1.128771923727	-1.267207150828
H	1.697327712431	2.046611252727	-1.217052763537
C	-1.136608815721	-2.516857588391	-2.066590073540
C	-2.363619757814	-0.076452003532	1.853087432141
C	0.182318886584	-2.455073484214	1.461079230344
H	0.584824289095	-3.463737951108	1.632456839328
H	-0.850803548753	-2.457966389774	1.817067918829
C	-3.289170613341	-0.369920460032	-0.253697319098
H	-3.116693318115	-0.419675788706	-1.320437137134
C	-0.891704899321	-2.983998906253	-0.650829620170
H	-1.813688224150	-2.878254368341	-0.070780291043
H	-0.618373455133	-4.046707255332	-0.637994971764
C	0.999248815763	-1.452170846158	2.267723500039
H	2.054503095241	-1.452801291273	1.979071762724
H	0.952434508841	-1.710527744680	3.329269477870
C	-1.216640384329	0.287885608321	2.772974150609
H	-1.225751494803	-0.339741278644	3.668845784674
H	-1.368795419195	1.321643085008	3.096720047755
C	3.623278900535	1.129733346527	-1.568801684909
H	4.120552003277	2.066290020199	-1.796123016690
C	3.626473968576	-1.243588269655	-1.230287343393

H	4.132471962463	-2.202751663435	-1.193328935018
C	1.478663580720	-2.424322569099	-0.637751352407
H	1.307479037659	-2.954385399258	-1.580337575424
H	2.075495545448	-3.101066324141	-0.015598982760
C	-4.566779146578	-0.531650294639	0.261016261489
H	-5.399817349980	-0.698179832661	-0.412606335956
C	-1.286004537705	-0.709547794960	-3.499304470120
H	-1.204092696077	0.365384498120	-3.617510892853
C	-3.622064149451	-0.253278069581	2.440091491931
H	-3.713127794521	-0.193816971330	3.519604356866
C	4.317661084120	-0.079966845470	-1.555081149169
H	5.378729950035	-0.115611118738	-1.780529312239
C	-1.464351759376	-3.404783678211	-3.091012660087
H	-1.513500785763	-4.470009269077	-2.890061024689
C	-1.721466488188	-2.899603415066	-4.365082900362
H	-1.980542724724	-3.568742383593	-5.179484314682
C	-4.737304685578	-0.485237016013	1.644652123737
H	-5.716437787118	-0.620874295245	2.092749657094
C	-1.630317957874	-1.524125458448	-4.575195277320
H	-1.817444538802	-1.088162096600	-5.550138058801
N	-0.847414670442	1.920013365548	-1.119462413568
O	-1.642786483148	2.924290686999	1.146679325798
S	-0.762580115874	3.161259850347	-0.021727226654
O	0.669116341066	3.410125125070	0.249596582475
C	-1.415787435526	4.618096372295	-0.849068462473
C	-0.541791619383	5.631889897039	-1.245848447074
C	-2.792933468050	4.735562205547	-1.071404632765
H	-3.475741223684	3.960131583366	-0.737543927334
H	0.520552366805	5.537090428988	-1.048121055482
C	-1.053153763125	6.764437470065	-1.883895696852
H	-0.372131953715	7.553836719862	-2.190201339763

C	-2.425554018262	6.900980518450	-2.131521862608
C	-3.284357477138	5.868911373673	-1.714843455003
C	-2.978671155180	8.133713448971	-2.804633662532
H	-4.353437393829	5.958932908525	-1.888984870370
H	-2.186534543754	8.720183058000	-3.279046365673
H	-3.718565318896	7.870957552848	-3.568598581302
H	-3.483394782773	8.782015701979	-2.076989968233
C	0.910321485682	2.520655588555	-4.558447477338
C	0.705481528732	1.971733125022	-5.804810460272
C	-0.564995249778	2.078246164544	-6.402693763553
C	-1.647116508336	2.713889634752	-5.762072380945
C	-1.458741854178	3.269064291197	-4.516312409884
C	-0.174650919833	3.168574866432	-3.837774646888
H	1.887519290971	2.470119778353	-4.088859131837
H	1.511897757300	1.478747356284	-6.336123997766
H	-0.714937554714	1.658725679960	-7.393506286368
H	-2.607477025574	2.777278206014	-6.261423058990
H	-2.270457179643	3.781208927691	-4.008591812217
H	-0.331588091157	2.648898642083	-2.798909888065
H	-1.845689029960	1.819863337832	-1.333783362832
H	0.133564538119	4.137521271320	-3.404149025960

$^{3}PC_{2c}$

Fe	-0.314917223811	-0.025251450386	-0.483744876845
S	0.464831853114	0.308890106691	2.011493629805
N	1.737307634745	-0.008174607152	-0.918499245139
N	-2.245130451332	-0.044058038107	0.552406324356
N	0.042552252883	-2.033219570871	-0.160813048949
N	-1.106364723941	-0.925229861887	-2.370204921989
C	2.288808440905	-1.240254485237	-0.940464091669
C	2.543400184032	1.065503963985	-1.050383006698
H	2.070420777966	2.036806925287	-0.978418786427

C	-1.310048761519	-2.252473546970	-2.212821805905
C	-2.385023398725	0.101179443962	1.886044975043
C	0.069945063564	-2.387896851013	1.294383820446
H	0.417432608871	-3.422913301632	1.409428137851
H	-0.959510796662	-2.351496953098	1.656690885338
C	-3.365548147114	-0.169337342259	-0.197991241009
H	-3.214435316534	-0.281920096985	-1.265356149288
C	-1.063863925634	-2.790911580331	-0.823956606449
H	-1.964136118320	-2.668009565947	-0.215666092400
H	-0.831738396469	-3.861928324574	-0.853926575679
C	0.943712131800	-1.462861172376	2.131002499095
H	1.993557927809	-1.496191283537	1.827422384682
H	0.899621018600	-1.773786414136	3.178483177715
C	-1.184195057782	0.306931999675	2.791117370018
H	-1.187930825205	-0.432254257082	3.598381213360
H	-1.276152802185	1.289272989779	3.261511342828
C	3.915777195423	0.949617806845	-1.236072302483
H	4.521112597424	1.842959132169	-1.340025766527
C	3.659014854525	-1.435123630011	-1.109635012226
H	4.064945898271	-2.440984078865	-1.114271701571
C	1.336892629850	-2.399164909066	-0.832656117426
H	1.106557559287	-2.740257699233	-1.846670902317
H	1.803017161046	-3.245338350155	-0.316985068725
C	-4.646655420025	-0.166026904825	0.332454651432
H	-5.502817194893	-0.268706134515	-0.324600406588
C	-1.318598980209	-0.380768253043	-3.582093622957
H	-1.129189356296	0.682516827970	-3.685538650804
C	-3.647860917021	0.099294787877	2.491751487753
H	-3.718796197018	0.211161310601	3.568910701075
C	4.484236212775	-0.324224318694	-1.268849295367
H	5.553502396357	-0.450770986958	-1.404795220854

C	-1.745125387697	-3.065291827101	-3.257247386029
H	-1.889840927461	-4.127821062506	-3.093823421785
C	-1.983343400747	-2.489522348106	-4.505221221503
H	-2.324878663857	-3.100856351538	-5.334347689576
C	-4.791378377495	-0.034832375006	1.714499124534
H	-5.774047465189	-0.034004108533	2.175130408466
C	-1.761334373995	-1.123424190607	-4.672655505021
H	-1.917442892327	-0.634941063206	-5.627336043477
N	-0.788427479639	1.943029929759	-1.086099260246
O	-1.161931281383	2.955509071706	1.260228270258
S	-0.577958271380	3.353207222591	-0.019933270342
O	0.842438474496	3.689761837223	-0.136108329859
C	-1.573814116547	4.631601341403	-0.750126603013
C	-0.964893482939	5.589083274602	-1.566738639581
C	-2.948261368110	4.658301755948	-0.485502410286
H	-3.405930429252	3.920683771041	0.165325209432
H	0.104179757030	5.563245863549	-1.745729253117
C	-1.758257095137	6.585419863903	-2.131883659700
H	-1.293626420834	7.336544998668	-2.764061075906
C	-3.140979091545	6.636793643712	-1.895534941977
C	-3.718948273405	5.660268092622	-1.066994025283
C	-3.986524331804	7.733496078924	-2.492319825316
H	-4.786662881663	5.688275905002	-0.869701844805
H	-4.992231766637	7.374909807653	-2.732822658570
H	-4.097409606416	8.562417735947	-1.781224948285
H	-3.533203580060	8.138541540546	-3.401717092904
C	1.700995012243	2.020153910749	-4.928535244845
C	1.155571011712	1.365416153148	-6.035890773512
C	-0.101168103724	1.741660112419	-6.522483956029
C	-0.816125172006	2.768122833500	-5.898065052760
C	-0.270244817932	3.426282133908	-4.790489139682

C	0.989916793463	3.054316332351	-4.308233439445
H	2.680597279046	1.735246873926	-4.554654129977
H	1.709931556371	0.568081121194	-6.523316161661
H	-0.519866214040	1.238413197063	-7.389925024079
H	-1.790792939240	3.061503596360	-6.278309804186
H	-0.817297336812	4.235120748257	-4.313890029837
H	-0.316309838786	2.173612873665	-1.968869223331
H	-1.788883962169	1.888548303471	-1.300823307550
H	1.428101077314	3.584242075899	-3.466055703722

$^{5}PC_{2c}$

Fe	-0.315415802875	-0.138605032163	-0.516564013456
S	0.554232005998	0.559185306308	1.860401638032
N	1.812961387088	-0.069315316275	-1.033696186477
N	-2.189329448699	-0.270375393800	0.698737841572
N	0.398134990734	-2.203989014043	0.130066447820
N	-1.014804673518	-1.477352107396	-2.156034528221
C	2.502092361845	-1.212147939057	-0.842414656839
C	2.480704524874	1.049800004246	-1.383699363990
H	1.889469290493	1.952597267918	-1.484616099282
C	-0.975173357458	-2.792242302022	-1.839195386338
C	-2.244263122336	0.001622719568	2.019856369100
C	0.467118697525	-2.282742236197	1.611659781844
H	0.951988452326	-3.219830300279	1.921836766790
H	-0.558418418135	-2.310855149368	1.986808843902
C	-3.311958757292	-0.709747859565	0.082731089090
H	-3.226339585140	-0.915285247474	-0.978095674930
C	-0.618127224427	-3.140960020352	-0.408298861467
H	-1.515124591479	-3.058552904638	0.213867415873
H	-0.278963997671	-4.182887655696	-0.347342871863
C	1.215324979514	-1.115861560375	2.254952253166
H	2.261692575387	-1.077710145188	1.939192686374

H	1.211951409120	-1.239119631833	3.341537247269
C	-1.044356773678	0.581737674183	2.741396987045
H	-0.917498545553	0.103489282696	3.716621270921
H	-1.234759657391	1.643501671441	2.923971455832
C	3.856632436824	1.067363709378	-1.575076192171
H	4.350613860072	1.990620130368	-1.856477052983
C	3.887811472277	-1.265786322880	-1.002061156347
H	4.414566877791	-2.198678487111	-0.830112804053
C	1.715675835940	-2.462084596505	-0.513056196462
H	1.536335527658	-2.990906444076	-1.455338891265
H	2.319222785041	-3.135885313252	0.106838589663
C	-4.515445769118	-0.904273322435	0.743650156668
H	-5.381575438005	-1.258148573753	0.196155822283
C	-1.357554152930	-1.124285482145	-3.409319326869
H	-1.370864016607	-0.061893516751	-3.626615557837
C	-3.420830553342	-0.187584107066	2.753307490288
H	-3.426528630160	0.030884769076	3.816179010508
C	4.573537297338	-0.114258985977	-1.380373347016
H	5.650828161820	-0.136372949752	-1.510710704143
C	-1.280742936577	-3.780615747650	-2.773798867197
H	-1.228349719045	-4.826161635044	-2.489274564592
C	-1.644314419015	-3.406589517261	-4.067007325077
H	-1.884284315082	-4.161817395908	-4.808559599341
C	-4.568157497948	-0.643807344152	2.114019396754
H	-5.486165481206	-0.792948290725	2.673457567285
C	-1.682677862132	-2.051406407165	-4.393347232316
H	-1.952109617818	-1.713514721143	-5.387667270903
N	-1.077541834609	1.887507693024	-1.439605764488
O	-1.257023563145	3.040679257405	0.833592287380
S	-0.814446914645	3.356433288558	-0.526267806161
O	0.581020874843	3.706989902662	-0.805429249704

C	-1.900907418574	4.583606204974	-1.218730869921
C	-1.492147747269	5.303124474375	-2.346598357699
C	-3.144715509835	4.804790055276	-0.619728660532
H	-3.439233711327	4.245308896663	0.261143365671
H	-0.522705967718	5.125627259700	-2.799813185552
C	-2.356566067845	6.255300497395	-2.878751757689
H	-2.048416611287	6.815798201022	-3.756803044765
C	-3.613617590069	6.503255711891	-2.302598077972
C	-3.990753372720	5.764484647618	-1.170474842175
C	-4.525495172337	7.559908711577	-2.874498270924
H	-4.958409575659	5.943494554400	-0.710592619624
H	-4.255429957092	8.551302710280	-2.488140363739
H	-4.448477805096	7.604873083272	-3.965457755604
H	-5.569771200111	7.375298632723	-2.605991998023
C	-0.915706079943	3.649488006043	-6.183202389058
C	-0.066196376831	4.717840095408	-5.881020026128
C	1.099508306095	4.498435448354	-5.139671897129
C	1.415957952721	3.209240860175	-4.700483425590
C	0.568292090614	2.138388734766	-5.006558119628
C	-0.597971751162	2.359292420772	-5.748213595891
H	-1.819271751151	3.820321381642	-6.761999211330
H	-0.309255697992	5.719205867383	-6.226251739045
H	1.762483690492	5.328410101120	-4.910623443868
H	2.326468856112	3.036281772520	-4.133555537846
H	0.829161773023	1.132309763426	-4.686964677285
H	-0.708537853822	2.033338623531	-2.385824212526
H	-2.087756072924	1.742102417616	-1.525648417725
H	-1.250967505763	1.527066165723	-5.998174732075
